# Sustainable Synthesis of 1,2,3‐Triazoles using Cyrene as a Biodegradable Solvent in Click Chemistry

**DOI:** 10.1002/cssc.202402538

**Published:** 2025-01-31

**Authors:** Andrea Citarella, Alessandro Fiori, Alessandra Silvani, Daniele Passarella, Valerio Fasano

**Affiliations:** ^1^ Dipartimento di Chimica, Università degli Studi di Milano Via Golgi 19 20133 Milano Italy

**Keywords:** Cyrene, sustainability, biodegradability, green chemistry, click chemistry

## Abstract

The first successful synthesis of 1,2,3‐triazoles using Cyrene^TM^ as a biodegradable and non‐toxic solvent in click chemistry has been developed. In contrast to previous methods, this sustainable approach allows product isolation by simple precipitation in water, eliminating the need for organic solvent extractions and column chromatography purifications, thus minimizing waste consumption while reducing operational costs. The protocol, performed also at gram scale, has broad applicability and versatility, as shown with complex substrates like biologically active coumarins or triazole‐linked bifunctional molecules. Finally, this protocol is also amenable to a three‐component reaction involving organic halides, terminal acetylenes and sodium azide, thus avoiding the isolation of organic azides, difficult‐to‐handle species known for their environmental sensitivity.

## Graphical abstract

Green triazoles: The first successful synthesis of 1,2,3‐triazoles using Cyrene as a biodegradable and non‐toxic solvent in click chemistry has been developed. This sustainable approach allows product isolation by simple precipitation in water, eliminating the need for organic solvent extractions and chromatographic purifications, thus minimizing waste consumption while reducing operational costs.

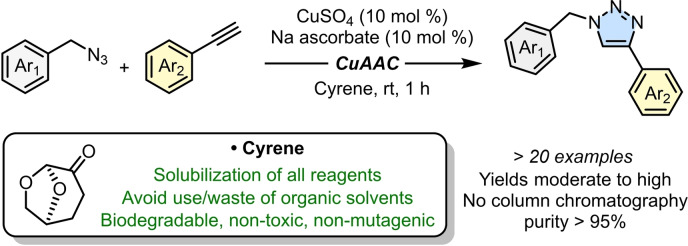

## Introduction

**Scheme 1:**
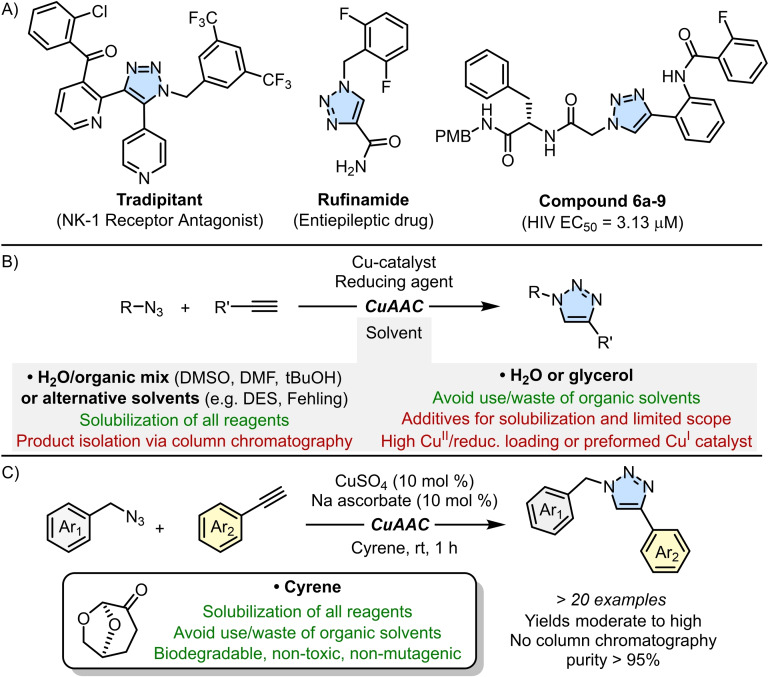
A) Examples of bioactive 1,2,3‐triazoles. B) CuAAC reaction performed in conventional system solvents. C) CuAAC reaction performed in Cyrene (this work).

1,2,3‐triazoles exhibit a wide range of biologically active properties, making them valuable compounds in medicinal chemistry.[^1^, ^2^] Their structural features enable triazoles to effectively mimic various functional groups (*e. g*. amides), which accounts for their extensive use as bioisosteres in the design and synthesis of new bioactive molecules.^[3]^ It is therefore not surprising that triazoles serve as scaffolds in the development of antimicrobial,^[4]^ antiepileptic,^[5]^ antiviral,[^6^, ^7^, ^8^, ^9^, ^10^] and anticancer drugs,[^11^, ^12^, ^13^] many of which have been approved by the FDA (Scheme 1A). Additionally, triazoles are widely used as linkers in hybrid molecules and conjugates, including Proteolysis‐targeting Chimeras (PROTACs), bifunctional molecules designed to degrade specific proteins.^[14]^ Consequently, there has been a significant interest in developing efficient and rapid methods for synthesizing 1,2,3‐triazoles. Among these methods, the copper‐catalyzed azide‐alkyne cycloaddition (CuAAC) reaction is considered the most effective (Scheme 1B).[^15^, ^16^] This reaction, often referred to as click chemistry, offers several advantages, including high 1,4‐regioselectivity, tolerance to a wide variety of functional groups, mild reaction conditions, simplicity, and high yields.[^17^, ^18^, ^19^, ^20^, ^21^] In the CuAAC reaction, solvents play a crucial role by solubilizing not only the coupling partners (azides and alkynes) but also the Cu^I^‐catalyst which is typically formed *in situ* mixing a Cu^II^ salt and a reducing agent.[^22^, ^23^, ^24^]

As a result, water is commonly used as a co‐solvent alongside an organic solvent such as dimethyl sulfoxide (DMSO), *N,N*‐dimethylformamide (DMF), or tetrahydrofuran (THF). However, DMSO and DMF are recognized as reprotoxic (H360) and should be replaced when possible, while THF is highly flammable and can form explosive peroxides when exposed to air or light.^[25]^ Alternatives like deep eutectic solvents[^26^, ^27^] or Fehling solutions^[28]^ have been successful in reducing the environmental impact, but as with organic solvent mixtures, product isolation typically requires extraction followed by column chromatography, which increases both cost and waste. Using only water (or glycerol) as the solvent can eliminate organic solvents, but the substrate scope may be limited due to solubility issues, requiring the use of surfactants or nanocatalysts.[^29^, ^30^, ^31^, ^32^, ^33^, ^34^, ^35^] Moreover, the rapid disproportionation of Cu^I^ in water necessitates higher catalyst loading or pre‐synthesized Cu^I^ catalysts. While other approaches, such as supercritical fluids or microwave‐ or mechanically‐assisted syntheses,[^36^, ^37^, ^38^] have been proposed, there is still significant room for improving the sustainability of 1,2,3‐triazole synthesis, particularly at the industrial scale, where solvents make up 80–90 % of the total non‐aqueous material and the majority of the waste generated.^[39]^ Recently, dihydrolevoglucosenone (branded as Cyrene™) has emerged as an environmentally friendly alternative to potentially hazardous solvents like DMF and DMSO.[^40^, ^41^, ^42^] Derived from cellulose, Cyrene is a biocompatible solvent that is stable at high temperatures (b.p.=227 °C) and can be safely used in microwave‐assisted reactions.^[43]^ It is non‐toxic, non‐mutagenic, and biodegradable, making it a sustainable choice for reducing environmental impact. Its high miscibility with water also allows for easy removal from reaction mixtures via an aqueous work‐up. Cyrene has already found use in various organic synthesis protocols, including amide bond formation, urea synthesis, S_N_Ar reactions, and Pd‐catalyzed reactions.[^44^, ^45^, ^46^, ^47^, ^48^, ^49^, ^50^] However, to our knowledge, Cyrene has not yet been employed as a solvent for synthesizing triazoles, with the exception of a single unsuccessful attempt.^[51]^ In this work, we present a mild and sustainable method for synthesizing 1,2,3‐triazoles using Cyrene as the sole solvent (Scheme 1C). Unlike recent advances in triazole synthesis that focus on novel catalysts and ligands,[^52^, ^53^] our approach is practical and widely applicable since it does not require complex preparations (all reagents are commercially available), with most of the synthesized triazoles obtained by simple precipitation in water, eliminating the need for purification by extractions with an organic solvent and column chromatography.

## Results and Discussion

Our investigation started by studying, as a model system, the CuAAC reaction between benzyl azide **1** (2.0 mmol) and phenylacetylene **2** (2.2 mmol), as shown in Table 1. CuSO_4_ and sodium ascorbate (both 10 mol %) were used as precursors of the Cu^I^‐catalyst, as often employed in click chemistry. When DMSO or DMF were used as co‐solvents in a 1 : 1 v/v mixture with H_2_O (entries 1 and 2), the desired product **3** was obtained in high yields after 1 hour at room temperature, although an aqueous work‐up followed by several extractions with ethyl acetate was necessary to remove these polar solvents. A better purification was achieved using a THF/H_2_O mixture (entry 3). In this case, evaporating the solvents under reduced pressure, followed by addition of water to the reaction mixture, led to the precipitation of pure product **3** in 77 % yield. While successful, the partial reduction in isolated yield and the need of prolonged heating under reduced pressure to remove water made the purification somehow improvable. Using Cyrene as the solvent led instead to several experimental differences. Unlike other solvents, Cyrene provided complete solubility for both organic and inorganic compounds (only CuSO_4_ was still partly insoluble), thus avoiding exposing potentially sensitive functional groups to the co‐solvent water. Additionally, being Cyrene highly viscose, a faster stirring rate was required to maintain uniformity throughout the solution.^[54]^ An initial attempt in Cyrene was carried out in a round‐bottom flask, under ambient conditions, at the concentration of 0.2 M (entry 4). Monitoring the reaction by HPLC‐MS, completion of the process was revealed after 4 hours, with product **3** isolated in 69 % yield after an aqueous work‐up followed by several washes of the organic phase with water to effectively remove all traces of Cyrene. Repeating the reaction in a vial at a 1 M concentration (entry 5), reaction completion was achieved in 1 hour (as judged by HPLC‐MS analysis) despite the lower solubility of CuSO_4_. Product **3** was easily isolated in 90 % yield by pouring the reaction mixture into ice‐water and collecting the resulting precipitate. In contrast to entry 4, no extraction with an organic solvent was necessary, with any residual Cyrene present in the precipitate effectively removed by washing with water. It has to be noted that this more sustainable protocol not only minimizes the environmental impact caused by wasted and toxic organic solvents but also lowers the risk associated with hazardous volatiles, such as aromatic or halogenated hydrocarbons. Furthermore, HPLC analysis revealed that product **3** was obtained in a purity exceeding 95 %, eliminating the need for column chromatography, which would otherwise contribute to additional waste of solvents and silica. Finally, reducing the loading of the catalyst precursors down to 1 mol % (entry 6) showed only a modest decrease in efficiency, a feature usually difficult to achieve performing the reaction exclusively in water (*vide supra*).

**Table 1:**
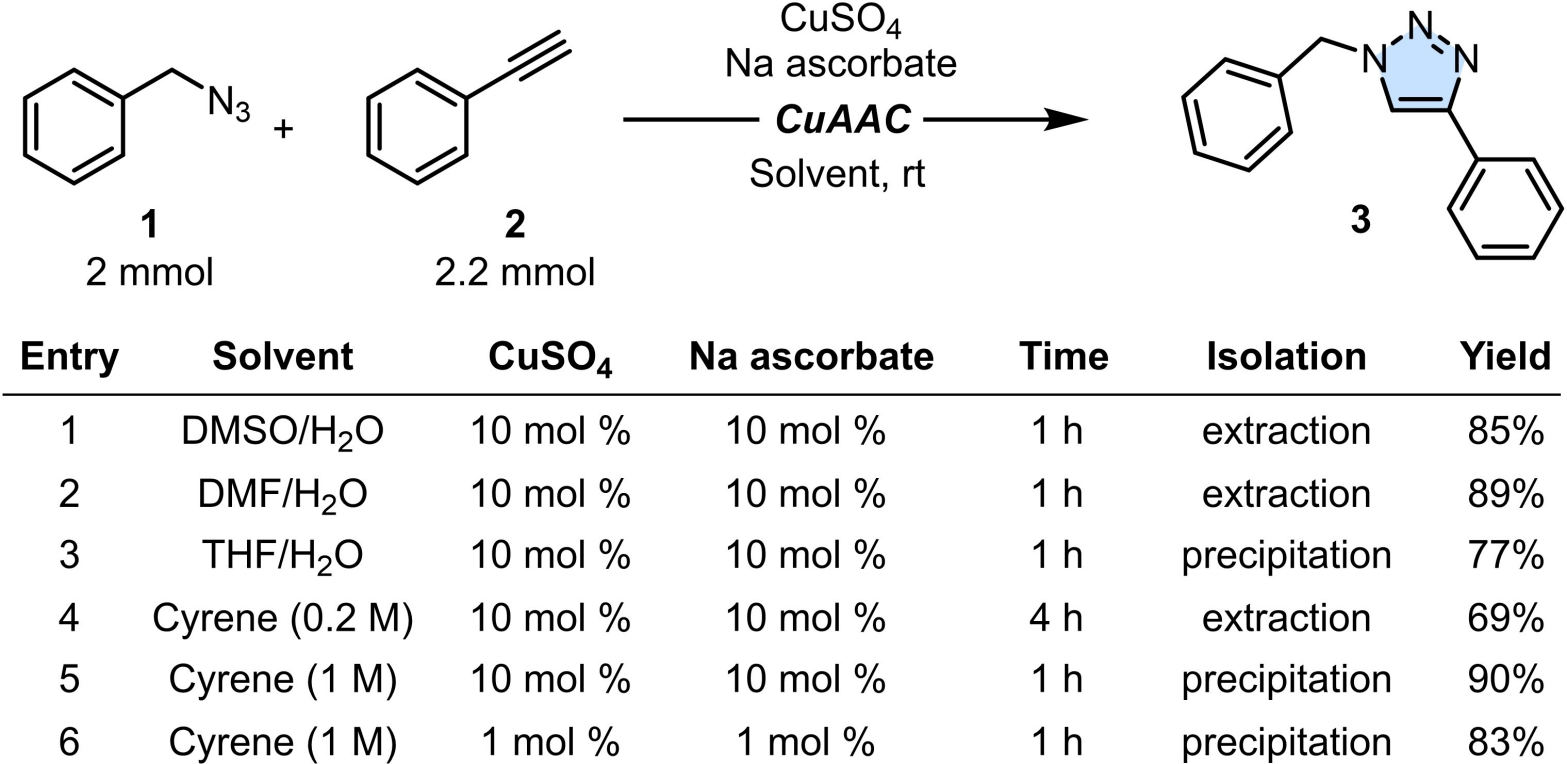
Optimization of the model reaction between azide **1** and alkyne **2**.

**Scheme 2:**
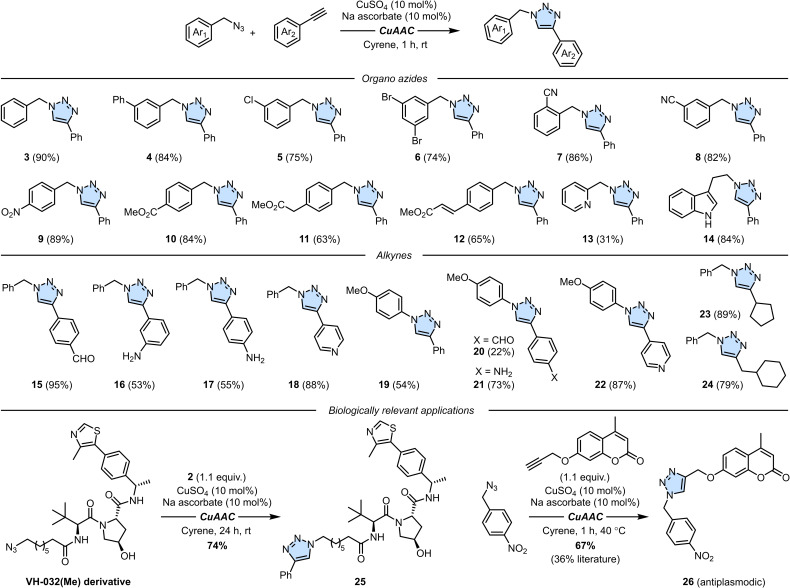
Substrate scope for the CuAAC reaction between several substituted organo azides and alkynes.

With the optimized conditions in hand, the scope of the reaction was explored using organic azides and alkynes commercially available or easily synthesized following established protocol.^[55]^ First, decorations on the organic azides were evaluated by using a slight excess (1.1 equiv) of phenylacetylene **2** with variously substituted benzyl azides (Scheme 2). The reactions, monitored by HPLC or TLC, reached completion within 1 hour at room temperature in all cases, and, after precipitation in ice‐water and filtration, the desired triazoles could be isolated in high yields and high purity (>95 %). The reaction proved to be effective with starting materials bearing various functional groups on the aromatic ring, including biphenyl (**4**), chlorine (**5**), bromine (**6**), cyano (**7**‐**8**), nitro (**9**). Sensitive functional groups such as esters remained intact during the reaction, allowing for the successful synthesis of compounds **10**–**12** in good yields. Replacing the benzene ring with a pyridine or an indole was also successful (**13**–**14**), although a lower yield (31 %) was observed for the former. Notably, **14**, obtained using the azido derivative of tryptamine and featuring a carbon chain longer than benzyl azides, showcase potential for application of triazoles as linkers in bifunctional molecules (*vide infra*).

The scope of the alkynes was then investigated using benzyl azide **1** or 4‐methoxyphenyl azide as starting materials, along with variously substituted alkynes. **1** reacted efficiently with 4‐ethynylbenzaldehyde, yielding **15** in 95 % yield. Alkynes bearing electron‐donating groups, such as amino groups in the *meta‐* and *para*‐positions, also proved compatibility, as shown for products **16**–**17**. Additionally, 4‐ethynylpyridine successfully reacted with **1**, although, in this specific case, pure product **18** did not precipitate from the reaction mixture, thus requiring an additional extraction with ethyl acetate. Substituted 4‐ethynylbenzenes also reacted with 4‐methoxyphenyl azide, furnishing product **19**–**21**. It has to be noted that precipitation of **20** did not afford a clean product, therefore an additional trituration in diethyl ether allowed a high purity at the expense of the yield. Finally, the reaction could be performed on the electron‐poor 4‐ethynylpyridine as well as on aliphatic alkynes, yielding products **22** and **23**–**24**, respectively. With the versatility of CuAAC reaction in Cyrene proven, some biologically relevant applications were tested too. The synthesis of triazole *via* click chemistry is among the most widely employed strategies for synthesizing PROTACs, heterobifunctional molecules with two active domains connected by a linker: one binding an E3 ubiquitin ligase (activating proteasomes), and the other targeting a protein for degradation.^[56]^ Using an azide derivative of VH‐032(Me) – an E3 ligase recruiter – and **2** as a model alkyne, after 24 hours, desired product **25** could be obtained in 74 % yield (raised temperature should be avoided to prevent degradation of the starting material). Also in this case, precipitation, filtration, and washing with water allowed to obtain **25** in high purity without any trace of Cyrene, thus demonstrating its potential for application in the synthesis of PROTACs. A second application of the click chemistry in Cyrene focused on the synthesis of triazole **26**, a coumarin derivative that showed *in vitro* antiplasmodial activity against a chloroquine‐sensitive strain of *Plasmodium falciparum*.^[57]^ Using a coumarin having a pendant propargyl group as the alkyne partner and 4‐nitrobenzyl azide, the CuAAC reaction, performed at 40 °C in a 0.3 M solution of Cyrene (to enhance solubility, as found upon optimisation), gave full conversion in 1 hour, as confirmed by HPLC analysis of the crude mixture. Trituration of the crude with ice‐cold water allowed the isolation of **26** by filtration in high purity and 67 % yield, notably exceeding the 36 % yield previously reported for the same reaction performed in *t*BuOH : H_2_O.^[57]^

**Scheme 3:**
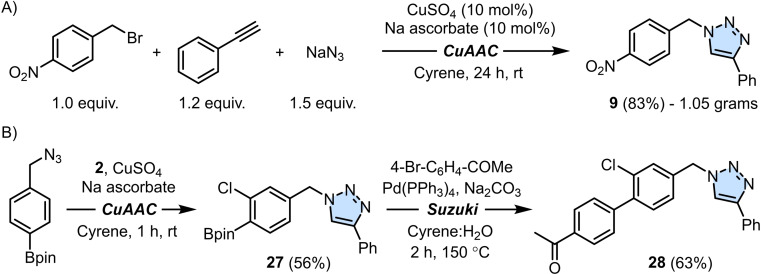
A) One‐pot three‐component CuAAC reaction in Cyrene. B) Tandem CuAAC reaction – Suzuki‐Miyaura cross‐coupling using Cyrene as the organic solvent.

Our investigation focused then on attempting a simple three‐component CuAAC reaction that would avoid the use of preformed organic azides, thus addressing the instability of the latter and the challenges associated with handling them. Starting from 4‐nitrobenzyl bromide, phenylacetylene **2** and sodium azide, desired product **9** could be obtained in 83 % yield through a one‐pot procedure in 24 hours under ambient conditions (Scheme 3A). Interestingly, purification of **9** by precipitation from the crude reaction mixture with ice‐cold water was not affected by the presence of NaN_3_ despite being partly soluble in Cyrene. This reaction could be scaled up to 1 gram of **9**, an important point from a safety point of view. Furthermore, by using stoichiometric amounts of NaN_3_ the inherent danger of this inorganic compound was notably minimized. Moreover, the procedure did not involve any concentration steps using a rotary evaporator, thereby avoiding the risk of explosion. Any unreacted azide was safely diluted in water during the washing step, following product filtration. These features notably improve the safety profile of the process, making Cyrene an attractive solvent for CuAAC reactions beyond its environmental benefits. The latter were measured during the synthesis of **3** at 2 gram‐scale and expressed as E‐factor (see Supporting Information).^[58]^ Finally, given the compatibility of Cyrene in Suzuki‐Miyaura cross‐coupling reactions,^[47]^ a one‐pot telescoping approach was attempted by performing first a CuAAC transformation, followed by a Pd‐catalyzed cross‐coupling reaction (Scheme 3B). This was successfully demonstrated for product **28** obtained in 63 % yield by treating triazole **27** with 4‐bromo acetophenone in the presence of Pd(PPh_3_)_4_ and Na_2_CO_3_ in a Cyrene : H_2_O mixture. This last application not only expands the scope of the CuAAC reaction in Cyrene to boronic esters, but also shows how Cyrene can be used as a replacement for traditional organic solvents across multiple synthetic steps.

## Conclusions

We developed a sustainable protocol for CuAAC reactions in Cyrene, enabling the synthesis of a diverse library of 1,2,3‐triazoles, including biologically relevant examples. This biodegradable solvent serves as an effective alternative to toxic solvents like DMF and DMSO. Moreover, after the reaction completion, the addition of ice‐water caused the precipitation of the desired triazoles in good yields and high purity. This avoids the need for extraction and column chromatography, significantly reducing costs and waste. Furthermore, the protocol does not require presynthetized organic azides, thus improving the safety of the process. Finally, this protocol is well‐suited for large‐scale production and one‐pot telescoping reactions, expanding its potential for eco‐friendly synthetic applications.

## Experimental Section

A mixture of the appropriate azide (1.0 equiv), the appropriate alkyne (1.1 equiv), CuSO4 ⋅ 5H2O (0.1 equiv) sodium ascorbate (0.1 equiv) in Cyrene (2 mL) was left stirring at room temperature for 1 h. The reaction mixture was poured into ice‐water and the precipitate was collected by filtration and washed with water to afford the pure product.

## Conflict of Interests

The authors declare no conflict of interest.

1

## Supporting information

As a service to our authors and readers, this journal provides supporting information supplied by the authors. Such materials are peer reviewed and may be re‐organized for online delivery, but are not copy‐edited or typeset. Technical support issues arising from supporting information (other than missing files) should be addressed to the authors.

Supporting Information

## Data Availability

The data that support the findings of this study are available in the supplementary material of this article.
