# Supplementary material for: Sustainable Synthesis of 1,2,3‐Triazoles using Cyrene as a Biodegradable Solvent in Click Chemistry
Source: ChemSusChem. 2025 Jan 31;18(10):e202402538. doi: 10.1002/cssc.202402538 (PMC12094146; doi:10.1002/cssc.202402538)
Supplement: Supplementary file 1 — Supporting Information [file CSSC-18-e202402538-s001.pdf]

ChemSusChem

Supporting Information

Sustainable Synthesis of 1,2,3-Triazoles using Cyrene as a Biodegradable Solvent in Click Chemistry

Andrea Citarella,* Alessandro Fiori, Alessandra Silvani, Daniele Passarella, and Valerio Fasano*

Supplementary materials for:

Sustainable synthesis of 1,2,3–triazoles using Cyrene as a biodegradable solvent in click chemistry

Andrea Citarella,[a]* Alessandro Fiori,[a] Alessandra Silvani,[a] Daniele Passarella,[a] and Valerio Fasano[a]*

[a] Dr. A. Citarella, A. Fiori, Prof. A. Silvani, Prof. D. Passarella, Prof. V. Fasano
Dipartimento di Chimica, Università degli Studi di Milano, Via Golgi 19, 20133, Milano (Italy)
E-mail: andrea.citarella@unimi.it; valerio.fasano@unimi.it

Table of contents **Pag.**

1.	Materials and Methods	1
2.	General Procedure	1
3.	Spectral and Characterization Data	1 – 6
4.	^{1}H- and ^{13}C-NMR Spectra for all the Compounds	7 – 32
5.	HPLC traces for the representative compounds	33 – 43
6.	E-factor calculation	44 – 45
7.	References	46

1. Materials and Methods

Unless otherwise stated, reagents and solvents were purchased from Merck (Milan, Italy), Fluorochem (Hadfield, United Kingdom) or BLDPharm (Reinbek, Germany) and used without further purification. All reactions were carried out in oven-dried glass-ware, using dry solvents, and monitored by TLC on silica gel (Merck precoated 60 F_{254} plates), with detection by UV light (254 nm) or by permanganate or by HPLC. HPLC was performed on Agilent 1100 Series System using a Gemini 5 μM C18 110 Å LC Column 150 × 3 mm and with a gradient of H_2O/ACN (+ 0.1% HCOOH) ranging from 5% ACN up to 100% ACN in 30 min (flux of 1.0 mL/min and sample injection of 20 μL), choosing 220 nm as the wavelength for the detection of compounds. Products were purified by flash column chromatography, using silica gel Merck 60 (230–400 mesh) as the stationary phase. Purity of the final compounds was assured to be >95% as assessed by NMR and HPLC. ^{1}H NMR and ^{13}C NMR spectra were recorded at 298 K on a Brüker Avance Spectrometer (400 MHz or 600 MHz), using commercially available deuterated solvents (DMSO-d_6, Chloroform-*d*). Only for compounds **18** and **22**, ^{1}H NMR spectra was recorded at 373 K. Chemical shifts are reported in parts per million (δ_{ppm}), compared to TMS as an internal standard. Coupling constants (*J*) are given in hertz (Hz) and are quoted to the nearest 0.5 Hz. Peak multiplicities are described in the following way: s, singlet; bs, broad singlet; d, doublet; m, multiplet; br, broad. Mass spectra were recorded on a Thermo Fisher LCQ Fleet Ion Trap Mass Spectrometer.

2. General Procedure for the synthesis of 1,2,3-triazoles in Cyrene

A mixture of the appropriate azide (1.0 equiv), the appropriate alkyne (1.1 equiv), $CuSO_4 \cdot 5H_2O$ (0.1 equiv) sodium ascorbate (0.1 equiv) in Cyrene (2 mL) was left stirring at room temperature for 1 h. The reaction mixture was poured into ice-water and the precipitate was collected by filtration and washed with water to afford the pure product.

Note: an aqueous work-up was necessary for compound **18**. The crude reaction mixture was diluted with ethyl acetate and the organic phase was washed five times with brine, dried with anhydrous Na_2SO_4, filtered and concentrated under *vacuum*.

3. Spectral and Characterization Data

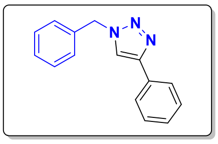

1-Benzyl-4-phenyl-1*H*-1,2,3-triazole (3).[1] By following the General procedure, starting from benzyl azide (266 mg, 2.0 mmol, 1.0 equiv), phenylacetylene (224 mg, 2.2 mmol, 1.1 equiv), $CuSO_4 \cdot 5H_2O$ (50 mg, 0.2 mmol, 0.1 equiv) and sodium ascorbate (40 mg, 0.2 mmol, 0.1 equiv) in Cyrene (2 mL), compound **3** was obtained with 90% yield (425 mg) as a yellow solid. ^{1}H NMR (400 MHz, Chloroform-*d*): δ 7.84 – 7.75 (m, 2H, Ar H), 7.66 (s, 1H, Triazole H), 7.44 – 7.35 (m, 5H, Ar H), 7.31 (m, 3H, Ar H), 5.58 (s, 2H, CH_2). ^{13}C NMR (100 MHz, Chloroform-*d*): δ 148.1, 134.7, 130.5, 129.1, 128.9, 128.8, 128.2, 128.0, 125.7, 119.7, 54.2. HRMS (ESI), *m/z* [M+H]$^+$: calculated for $C_{15}H_{14}N_3^+$ 236.1182; found 236.1199.

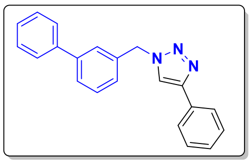

1-([1,1'-Biphenyl]-3-ylmethyl)-4-phenyl-1*H*-1,2,3-triazole (4).[2] By following the General procedure, starting from 3-(azidomethyl)-1,1'-biphenyl (293 mg, 1.4 mmol, 1.0 equiv), phenylacetylene (157 mg, 1.54 mmol, 1.1 equiv), $CuSO_4 \cdot 5H_2O$ (37 mg, 0.15 mmol, 0.1 equiv) and sodium ascorbate (30 mg, 0.15 mmol, 0.1 equiv) in Cyrene (2 mL), compound **4** was obtained with 84% yield (364 mg) as a yellow solid. ^{1}H NMR (400 MHz, Chloroform-*d*): δ 7.81 (d, *J* = 7.6 Hz, 2H, Ar H), 7.71 (s, 1H, Triazole H), 7.63 – 7.52 (m, 4H, Ar H), 7.50 – 7.27 (m, 8H, Ar H), 5.64 (s, 2H, CH_2). ^{13}C NMR (100 MHz, Chloroform-*d*): δ 148.3, 142.3, 140.3, 135.3, 129.7, 129.0, 128.9, 128.2, 127.8, 127.6, 127.2, 126.9, 125.8, 119.7, 54.3. HRMS (ESI), *m/z* [M+H]$^+$: calculated for $C_{21}H_{18}N_3^+$ 312.1495; found 312.1490.

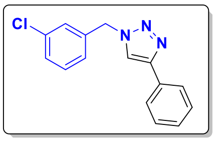

1-(3-chlorobenzyl)-4-phenyl-1*H*-1,2,3-triazole (5).[3] By following the General procedure, starting from 1-(azidomethyl)-3-chlorobenzene (334 mg, 2.0 mmol, 1.0 equiv), phenylacetylene (224 mg, 2.2 mmol, 1.1 equiv), $CuSO_4 \cdot 5H_2O$ (50 mg, 0.2 mmol, 0.1 equiv) and sodium ascorbate (40 mg, 0.2 mmol, 0.1 equiv) in Cyrene (2 mL), compound **5** was obtained with 75% yield (406 mg) as a white solid. ^{1}H NMR (400 MHz, Chloroform-*d*): δ 7.78 (d, *J* = 7.4 Hz, 2H, Ar H), 7.70 (s, 1H, Triazole H), 7.37 (t, *J* = 7.5 Hz, 2H, Ar H), 7.32 – 7.23 (m, 4H,

Ar H), 7.13 (d, *J* = 6.9 Hz, 1H, Ar H), 5.49 (s, 2H, CH_2). ^{13}C NMR (100 MHz, Chloroform-*d*): δ 148.4, 136.8, 135.0, 130.5, 130.4, 129.0, 128.9, 128.3, 128.1, 126.1, 125.8, 119.7, 53.5. HRMS (ESI), *m/z* $[M+H]^+$: calculated for $C_{15}H_{13}Cl_2N_3^+$ 270.0793; found 270.0729.

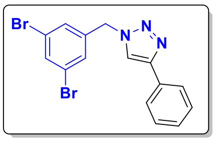

1-(3,5-dibromobenzyl)-4-phenyl-1*H*-1,2,3-triazole (6). By following the General procedure, starting from 1-(azidomethyl)-3,5-dibromobenzene (269 mg, 0.93 mmol, 1.0 equiv), phenylacetylene (104 mg, 1.02 mmol, 1.1 equiv), $CuSO_4 \cdot 5H_2O$ (22 mg, 0.09 mmol, 0.1 equiv) and sodium ascorbate (18 mmol, 0.09 mmol, 0.1 equiv) in Cyrene (2 mL), compound **6** was obtained with 74% yield (272 mg) as a yellow solid. ^1H NMR (400 MHz, Chloroform-*d*): δ 7.84 (s, 1H, Ar H), 7.82 (s, 1H, Ar H), 7.73 (s, 1H, Triazole H), 7.67 (s, 1H, Ar H), 7.46 – 7.33 (m, 5H, Ar H), 5.52 (s, 2H, CH_2). ^{13}C NMR (100 MHz, Chloroform-*d*): δ 148.8, 138.5, 134.7, 130.3, 129.8, 129.0, 128.6, 125.9, 123.8, 119.7, 52.9. HRMS (ESI), *m/z* $[M+H]^+$: calculated for $C_{15}H_{12}Br_2N_3^+$ 393.9372; found 393.9351.

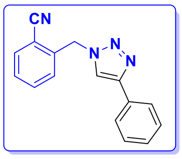

2-((4-phenyl-1*H*-1,2,3-triazol-1-yl)methyl)benzonitrile (7).[4] By following the General procedure, starting from 2-(azidomethyl)benzonitrile (219 mg, 1.38 mmol, 1.0 equiv), phenylacetylene (155 mg, 1.52 mmol, 1.1 equiv), $CuSO_4 \cdot 5H_2O$ (32 mg, 0.13 mmol, 0.1 equiv) and sodium ascorbate (26 mmol, 0.13 mmol, 0.1 equiv) in Cyrene (2 mL), compound **7** was obtained with 86% yield (318 mg) as a yellow solid. ^1H NMR (400 MHz, Chloroform-*d*): δ 7.90 (s, 1H, Triazole H), 7.83 (d, *J* = 7.4 Hz, 2H, Ar H), 7.74 (d, *J* = 7.7 Hz, 1H, Ar H), 7.61 (t, *J* = 7.5 Hz, 1H, Ar H), 7.48 (t, *J* = 7.6 Hz, 1H, Ar H), 7.42 (t, *J* = 7.4 Hz, 3H, Ar H), 7.33 (t, *J* = 7.4 Hz, 1H, Ar H), 5.81 (s, 2H, CH_2). ^{13}C NMR (100 MHz, Chloroform-*d*): δ 148.6, 138.3, 133.8, 133.2, 130.3, 129.7, 129.5, 129.0, 128.5, 125.9, 120.2, 117.2, 111.9, 51.9. HRMS (ESI), *m/z* $[M+H]^+$: calculated for $C_{16}H_{13}N_4^+$ 261.1135; found 261.1149.

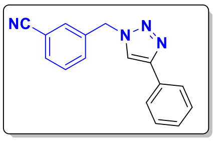

3-((4-Phenyl-1*H*-1,2,3-triazol-1-yl)methyl)benzonitrile (8). By following the General procedure, starting from 3-(azidomethyl)benzonitrile (292 mg, 1.85 mmol, 1.0 equiv), phenylacetylene (208 mg, 2.04 mmol, 1.1 equiv), $CuSO_4 \cdot 5H_2O$ (45 mg, 0.18 mmol, 0.1 equiv) and sodium ascorbate (36 mmol, 0.18 mmol, 0.1 equiv) in Cyrene (2 mL), compound **8** was obtained with 82% yield (395 mg) as a yellow solid. ^1H NMR (400 MHz, Chloroform-*d*): δ 7.81 (d, *J* = 7.6 Hz, 2H, Ar H), 7.74 (s, 1H, Triazole H), 7.66 (d, *J* = 6.7 Hz, 1H, Ar H), 7.60 (s, 1H, Ar H), 7.51 (d, *J* = 7.3 Hz, 2H, Ar H), 7.42 (t, *J* = 7.4 Hz, 2H, Ar H), 7.34 (t, *J* = 7.2 Hz, 1H, Ar H), 5.62 (s, 2H, CH_2). ^{13}C NMR (100 MHz, DMSO-d_6): δ 146.7, 137.4, 132.9, 132.0, 131.7, 130.5, 130.1, 128.9, 127.9, 125.2, 121.8, 118.4, 111.7, 52.0. HRMS (ESI), *m/z* $[M+H]^+$: calculated for $C_{16}H_{13}N_4^+$ 261.1135; found 261.1156.

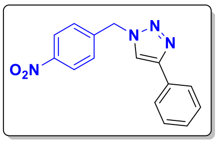

1-(4-Nitrobenzyl)-4-phenyl-1*H*-1,2,3-triazole (9).[5] By following the General procedure, starting from 1-(azidomethyl)-4-nitrobenzene (258 mg, 1.45 mmol, 1.0 equiv), phenylacetylene (163 mg, 1.6 mmol, 1.1 equiv), $CuSO_4 \cdot 5H_2O$ (36 mg, 0.14 mmol, 0.1 equiv) and sodium ascorbate (28 mg, 0.14 mmol, 0.1 equiv) in Cyrene (2 mL), compound **9** was obtained with 89% yield (363 mg) as a yellow solid. ^1H NMR (400 MHz, Chloroform-*d*): δ 8.24 (d, J = 8.5 Hz, 2H, Ar H), 7.81 (d, J = 7.6 Hz, 2H, Ar H), 7.75 (s, 1H, Triazole H), 7.50 – 7.39 (m, 4H, Ar H), 7.34 (t, J = 7.4 Hz, 1H, Ar H), 5.70 (s, 2H, CH_2). ^{13}C NMR (100 MHz, Chloroform-*d*): δ 148.7, 148.1, 141.9, 130.2, 129.0, 128.6, 127.85, 125.81, 124.4, 119.9, 53.2. HRMS (ESI), *m/z* $[M+H]^+$: calculated for $C_{15}H_{12}N_4NaO_2^+$ 303.0852; found 303.0869.

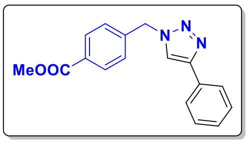

Methyl 4-((4-phenyl-1*H*-1,2,3-triazol-1-yl)methyl)benzoate (10).[6] By following the General procedure, starting from methyl 4-(azidomethyl)benzoate (277 mg, 1.45 mmol, 1.0 equiv), phenylacetylene (163 mg, 1.6 mmol, 1.1 equiv), $CuSO_4 \cdot 5H_2O$ (36 mg, 0.14 mmol, 0.1 equiv) and sodium ascorbate (28 mg, 0.14 mmol, 0.1 equiv) in Cyrene (2 mL), compound **10** was obtained with 84% yield (357 mg) as a yellow solid. ^1H NMR (400 MHz, Chloroform-*d*): δ 8.05 (d, *J* = 8.3 Hz, 2H, Ar H), 7.80 (d, *J* = 7.9 Hz, 2H, Ar H), 7.70 (s, 1H, Triazole H), 7.38 (dt, *J* = 25.7, 7.3 Hz, 5H, Ar H), 5.64 (s, 2H, CH_2), 3.92 (s, 3H, CH_3). ^{13}C NMR (100 MHz, Chloroform-*d*): δ 166.5, 148.6, 139.7, 130.7, 130.5, 129.7, 129.0, 128.4, 127.9, 125.8, 119.7, 53.8, 52.4. HRMS (ESI), *m/z* $[M+H]^+$: calculated for $C_{17}H_{15}N_3NaO_2^+$ 316.1056; found 316.1080.

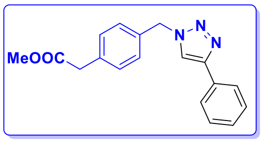

Methyl 2-(4-((4-phenyl-1*H*-1,2,3-triazol-1-yl)methyl)phenyl)acetate (11). By following the General procedure, starting from methyl 2-(4-(azidomethyl)phenyl)acetate (279 mg, 1.36 mmol, 1.0 equiv), phenylacetylene (153 mg, 1.5 mmol, 1.1 equiv), $CuSO_4 \cdot 5H_2O$ (35 mg, 0.14 mmol, 0.1 equiv) and sodium ascorbate (28 mg, 0.14 mmol, 0.1 equiv) in Cyrene (2 mL), compound **11** was

obtained with 63% yield (263 mg) as a white solid. ^{1}H NMR (400 MHz, Chloroform-*d*): δ 7.80 (d, *J* = 7.2 Hz, 2H, Ar H), 7.67 (s, 1H, Triazole H), 7.40 (t, *J* = 7.5 Hz, 2H, Ar H), 7.31 (dd, *J* = 14.8, 6.7 Hz, 5H, Ar H), 5.56 (s, 2H, CH_2), 3.69 (s, 3H, CH_3), 3.64 (s, 2H, CH_2). ^{13}C NMR (100 MHz, Chloroform-*d*): δ 171.7, 134.8, 133.7, 130.2, 129.8, 128.9, 128.4, 128.3, 125.8, 119.7, 54.5, 53.9, 52.2, 40.8. HRMS (ESI), *m/z* $[M+H]^+$: calculated for $C_{18}H_{18}N_3O_2^+$ 308.1394; found 308.1367.

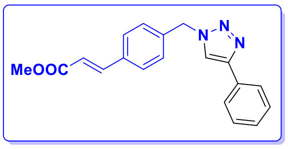

Methyl (*E*)-3-(4-((4-phenyl-1*H*-1,2,3-triazol-1-yl)methyl)phenyl)acrylate (12).[7] By following the General procedure, starting from methyl (*E*)-3-(4-(azidomethyl)phenyl)acrylate (313 mg, 1.44 mmol, 1.0 equiv), phenylacetylene (161 mg, 1.58 mmol, 1.1 equiv), $CuSO_4 \cdot 5H_2O$ (35 mg, 0.14 mmol, 0.1 equiv) and sodium ascorbate (28 mg, 0.14 mmol, 0.1 equiv) in Cyrene (2 mL), compound **12** was obtained with 65% yield (297 mg) as a white solid. ^{1}H NMR (400 MHz, Chloroform-*d*): δ 7.80 (d, *J* = 7.6 Hz, 2H, Ar H), 7.67 (d, *J* = 16 Hz, 2H, CH=CH), 7.54 (s, 2H, Triazole H), 7.42 (s, 2H, Ar H), 7.32 (d, *J* = 8.2 Hz, 3H, Ar H), 6.44 (d, *J* = 16 Hz, 1H, CH=CH), 5.59 (s, 2H, CH_2), 3.81 (s, 3H, CH_3). ^{13}C NMR (100 MHz, Chloroform-*d*): δ 167.3, 148.5, 143.8, 136.8, 135.0, 130.4, 129.0, 128.8, 128.6, 128.4, 125.8, 119.7, 118.9, 53.9, 51.9. HRMS (ESI), *m/z* $[M+H]^+$: calculated for $C_{19}H_{18}N_3O_2^+$ 320.1394; found 320.1423.

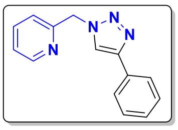

2-((4-phenyl-1*H*-1,2,3-triazol-1-yl)methyl)pyridine (13).[8] By following the General procedure, starting from 2-(azidomethyl)pyridine (166 mg, 1.21 mmol, 1.0 equiv), phenylacetylene (136 mg, 1.33 mmol, 1.1 equiv), $CuSO_4 \cdot 5H_2O$ (30 mg, 0.12 mmol, 0.1 equiv) and sodium ascorbate (24 mg, 0.12 mmol, 0.1 equiv) in Cyrene (2 mL), compound **13** was obtained with 31% yield (90 mg) as a yellow solid. ^{1}H NMR (400 MHz, Chloroform-*d*): δ 8.62 (s, 1H, Ar H), 7.94 (s, 1H, Triazole H), 7.83 (d, *J* = 8.5 Hz, 2H, Ar H), 7.69 (t, *J* = 8.5 Hz, 1H, Ar H), 7.41 (t, *J* = 7.5 Hz, 2H, Ar H), 7.35 – 7.30 (m, 1H, Ar H), 7.30 – 7.20 (m, 2H, Ar H), 5.70 (s, 2H, CH_2). ^{13}C NMR (100 MHz, Chloroform-*d*): δ 154.5, 149.8, 148.2, 137.4, 130.6, 128.9, 128.2, 125.7, 123.5, 122.5, 120.3, 55.7. HRMS (ESI), *m/z* $[M+H]^+$: calculated for $C_{15}H_{14}N_3^+$ 236.1182; found 236.1199.

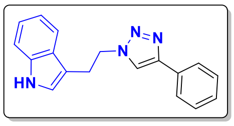

3-(2-(4-phenyl-1H-1,2,3-triazol-1-yl)ethyl)-1H-indole (14). By following the General procedure, starting from 2-(2-azidoethyl)-1*H*-indole (280 mg, 1.5 mmol, 1.0 equiv), phenylacetylene (169 mg, 1.65 mmol, 1.1 equiv), $CuSO_4 \cdot 5H_2O$ (37 mg, 0.15 mmol, 0.1 equiv) and sodium ascorbate (30 mg, 0.15 mmol, 0.1 equiv) in Cyrene (2 mL), compound **14** was obtained with 84% yield (357 mg) as a yellow solid. ^{1}H NMR (400 MHz, Chloroform-*d*): δ 8.12 (s, 1H, NH), 7.74 (d, *J* = 7.3 Hz, 2H, Ar H), 7.59 (d, *J* = 7.9 Hz, 1H, Ar H), 7.44 (s, 1H, Triazole H), 7.39 (dt, *J* = 7.6, 3.7 Hz, 3H, Ar H), 7.32 (q, *J* = 7.3, 6.2 Hz, 1H, Ar H), 7.23 (t, *J* = 7.4 Hz, 1H, Ar H), 7.16 (t, *J* = 7.3 Hz, 1H, Ar H), 6.85 (d, *J* = 2.4 Hz, 1H, Ar H), 4.70 (t, *J* = 6.9 Hz, 2H, CH_2), 3.42 (t, *J* = 6.9 Hz, 2H, CH_2). ^{13}C NMR (100 MHz, Chloroform-*d*): δ 148.0, 147.5, 136.5, 130.8, 128.9, 128.2, 126.9, 125.8, 122.9, 122.5, 120.3, 119.8, 118.4, 111.6, 50.9, 26.7. HRMS (ESI), *m/z* $[M+H]^+$: calculated for $C_{18}H_{17}N_4^+$ 298.1448; found 298.1419.

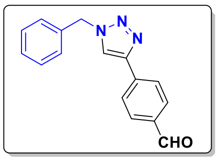

4-(1-Benzyl-1*H*-1,2,3-triazol-4-yl)benzaldehyde (15).[9] By following the General procedure, starting from benzyl azide (266 mg, 2.0 mmol, 1.0 equiv), 4-ethynylbenzaldehyde (286 mg, 2.2 mmol, 1.1 equiv), $CuSO_4 \cdot 5H_2O$ (100 mg, 0.4 mmol, 0.2 equiv) and sodium ascorbate (79 mmol, 0.4 mmol, 0.2 equiv) in Cyrene (2 mL), compound **15** was obtained with 95% yield (500 mg) as an orange solid. ^{1}H NMR (400 MHz, DMSO-*d*$_6$): δ 10.01 (s, 1H, CHO), 8.84 (s, 1H, Triazole H), 8.08 (d, *J* = 8.4 Hz, 2H, Ar H), 7.98 (d, *J* = 8.4 Hz, 2H, Ar H), 7.46 – 7.30 (m, 5H, Ar H), 5.68 (s, 2H, CH_2). ^{13}C NMR (100 MHz, DMSO-*d*$_6$): δ 192.5, 145.6, 136.3, 135.8, 135.4, 130.3, 128.9, 128.3, 128.0, 125.6, 123.2, 53.2. HRMS (ESI), *m/z* $[M+H]^+$: calculated for $C_{16}H_{14}N_3O^+$ 264.1131; found 264.1149.

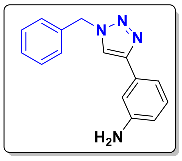

3-(1-Benzyl-1*H*-1,2,3-triazol-4-yl)aniline (16).[10] By following the General procedure, starting from benzyl azide (266 mg, 2.0 mmol, 1.0 equiv), 3-ethynylaniline (258 mg, 2.2 mmol, 1.1 equiv), $CuSO_4 \cdot 5H_2O$ (50 mg, 0.2 mmol, 0.1 equiv) and sodium ascorbate (40 mmol, 0.2 mmol, 0.1 equiv) in Cyrene (2 mL), compound **16** was obtained with 53% yield (267 mg) as a green solid. ^{1}H NMR (400 MHz, DMSO-*d*$_6$): δ 8.45 (s, 1H, Triazole H), 7.43 – 7.30 (m, 5H, Ar H), 7.09 (s, 1H, Ar H), 7.05 (t, *J* = 7.8 Hz, 1H, Ar H), 6.92 (d, *J* = 7.8 Hz, 1H, Ar H), 6.51 (d, *J* = 9.0 Hz, 1H, Ar H), 5.61 (s, 2H, CH_2), 5.09 (bs, 2H, NH_2). ^{13}C NMR (100 MHz, DMSO-*d*$_6$): δ 148.9, 147.5, 136.1, 131.1, 129.4, 128.9, 128.2, 128.0, 121.2, 113.9, 113.3, 110.7. HRMS (ESI), *m/z* $[M+H]^+$: calculated for $C_{15}H_{15}N_4^+$ 251.1291; found 251.1348.

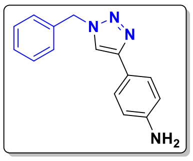

4-(1-Benzyl-1*H*-1,2,3-triazol-4-yl)aniline (17).[11] By following the General procedure, starting from benzyl azide (266 mg, 2.0 mmol, 1.0 equiv), 4-ethynylaniline (258 mg, 2.2 mmol, 1.1 equiv), $CuSO_4 \cdot 5H_2O$ (50 mg, 0.2 mmol, 0.1 equiv) and sodium ascorbate (40 mmol, 0.2 mmol, 0.1 equiv) in Cyrene (2 mL), compound **17** was obtained with 55% yield (278 mg) as a brown solid. ^{1}H NMR (400 MHz, DMSO-d_6): δ 8.31 (s, 1H, Triazole H), 7.49 (d, *J* = 8.2 Hz, 2H, Ar H), 7.42 – 7.25 (m, 5H, Ar H), 6.59 (d, *J* = 8.2 Hz, 2H, Ar H), 5.58 (s, 2H, CH_2), 5.16 (bs, 2H, NH_2). ^{13}C NMR (100 MHz, DMSO-d_6): δ 147.6, 136.1, 129.5, 128.7, 128.0, 127.8, 126.0, 119.4, 118.5, 114.2, 52.9. HRMS (ESI), *m/z* $[M+H]^+$: calculated for $C_{15}H_{15}N_4^+$ 251.1291; found 251.1327.

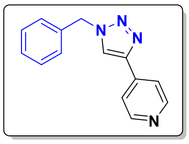

4-(1-Benzyl-1*H*-1,2,3-triazol-4-yl)pyridine (18).[12] By following the General procedure, starting from benzyl azide (266 mg, 2.0 mmol, 1.0 equiv), 4-ethynylpyridine (227 mg, 2.2 mmol, 1.1 equiv), $CuSO_4 \cdot 5H_2O$ (50 mg, 0.2 mmol, 0.1 equiv) and sodium ascorbate (40 mg, 0.2 mmol, 0.1 equiv) in Cyrene (2 mL), compound **18** was obtained with 88% yield (418 mg) as a white solid after an aqueous work-up. ^{1}H NMR (400 MHz, DMSO-d_6): δ 8.73 (s, 1H, Triazole H), 8.64 (bs, 2H, Pyr H), 7.82 (bs, 2H, Pyr H), 7.46 – 7.27 (m, 5H, Ph H), 5.67 (s, 2H, CH_2). ^{13}C NMR (100 MHz, DMSO-d_6): δ 150.2, 144.4, 137.6, 135.7, 128.8, 128.3, 128.0, 123.6, 120.2, 53.2. HRMS (ESI), *m/z* $[M+H]^+$: calculated for $C_{14}H_{13}N_4^+$ 237.1135; found 237.1112.

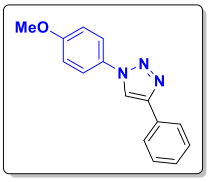

1-(4-Methoxyphenyl)-4-phenyl-1*H*-1,2,3-triazole (19).[13] By following the General procedure, starting from 1-azido-4-methoxybenzene (298 mg, 2.0 mmol, 1.0 equiv), phenylacetylene (224 mg, 2.2 mmol, 1.1 equiv), $CuSO_4 \cdot 5H_2O$ (50 mg, 0.2 mmol, 0.1 equiv) and sodium ascorbate (40 mg, 0.2 mmol, 0.1 equiv) in Cyrene (2 mL), compound **19** was obtained with 54% yield (270 mg) as a yellow solid. ^{1}H NMR (400 MHz, DMSO-d_6): δ 9.20 (s, 1H, Triazole H), 7.94 (d, *J* = 7.3 Hz, 2H, Ar H), 7.86 (d, *J* = 9.0 Hz, 2H), 7.50 (t, *J* = 7.4 Hz, 2H, Ar H), 7.38 (t, *J* = 7.4 Hz, 1H, Ar H), 7.18 (d, *J* = 9.0 Hz, 2H, Ar H), 3.85 (s, 3H, OCH_3). ^{13}C NMR (100 MHz, DMSO-d_6): δ 159.3, 147.1, 130.4, 130.1, 129.0, 128.2, 125.3, 121.7, 119.6, 114.9, 55.6. HRMS (ESI), *m/z* $[M+H]^+$: calculated for $C_{15}H_{14}N_3O^+$ 252.1131; found 252.1147.

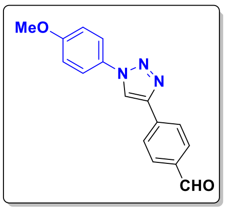

4-(1-(4-Methoxyphenyl)-1*H*-1,2,3-triazol-4-yl)benzaldehyde (20). By following the General procedure, starting from 1-azido-4-methoxybenzene (75 mg, 0.5 mmol, 1.0 equiv), 4-ethynylbenzaldehyde (56 mg, 0.55 mmol, 1.1 equiv), $CuSO_4 \cdot 5H_2O$ (12 mg, 0.05 mmol, 0.1 equiv) and sodium ascorbate (10 mg, 0.05 mmol, 0.1 equiv) in Cyrene (1 mL), compound **20** was obtained with 22% yield (28 mg) as a brown solid. ^{1}H NMR (400 MHz, Chloroform-*d*): δ 10.05 (s, 1H, CHO), 8.23 (s, 1H, Triazole H), 8.09 (d, *J* = 7.5 Hz, 2H, Ar H), 7.98 (d, *J* = 7.5 Hz, 2H, Ar H), 7.69 (d, *J* = 8.2 Hz, 2H, Ar H), 7.06 (d, *J* = 8.2 Hz, 2H, Ar H), 3.89 (s, 3H, CH_3). ^{13}C NMR (100 MHz, Chloroform-*d*): δ 191.8, 160.3, 147.1, 136.3, 136.1, 130.6, 130.4, 126.3, 122.4, 119.1, 115.1, 55.8. HRMS (ESI), *m/z* $[M+H]^+$: calculated for $C_{16}H_{14}N_3O_2^+$ 280.1081; found 280.1007.

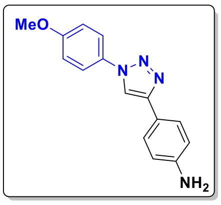

4-(1-(4-methoxyphenyl)-1*H*-1,2,3-triazol-4-yl)aniline (21).[14] By following the General procedure, starting from 1-azido-4-methoxybenzene (37 mg, 0.25 mmol, 1.0 equiv), 4-ethynylaniline (33 mg, 0.28 mmol, 1.1 equiv), $CuSO_4 \cdot 5H_2O$ (7 mg, 0.03 mmol, 0.1 equiv) and sodium ascorbate (5 mg, 0.03 mmol, 0.1 equiv) in Cyrene (1 mL), compound **21** was obtained with 73% yield (49 mg) as an orange solid. ^{1}H NMR (400 MHz, DMSO-d_6): δ 8.87 (s, 1H, Triazole H), 7.82 (d, *J* = 8.8 Hz, 2H, Ar H), 7.59 (d, *J* = 7.4 Hz, 2H, Ar H), 7.15 (d, *J* = 8.8 Hz, 2H, Ar H), 6.62 (d, *J* = 7.4 Hz, 2H, Ar H), 3.83 (s, 3H, CH_3), NH_2 exchanges. ^{13}C NMR (100 MHz, DMSO-d_6): δ 159.1, 148.0, 133.5, 130.2, 126.2, 121.4, 118.1, 117.3, 114.8, 114.2, 55.5. HRMS (ESI), *m/z* $[M+H]^+$: calculated for $C_{15}H_{14}N_4NaO^+$ 289.1060; found 289.1099.

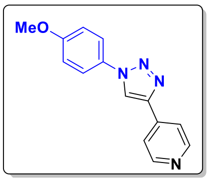

4-(1-(4-Methoxyphenyl)-1*H*-1,2,3-triazol-4-yl)pyridine (22).[15] By following the General procedure, starting from 1-azido-4-methoxybenzene (37 mg, 0.25 mmol, 1.0 equiv), 4-ethynylpyridine (29 mg, 0.28 mmol, 1.1 equiv), $CuSO_4 \cdot 5H_2O$ (7 mg, 0.03 mmol, 0.1 equiv) and sodium ascorbate (5 mg, 0.03 mmol, 0.1 equiv) in Cyrene (1 mL), compound **22** was obtained with 87% yield (55 mg) as a grey solid. ^{1}H NMR (400 MHz, DMSO-d_6): δ 9.18 (bs, 3H, Triazole H, Pyr H), 8.02 (s, 2H, Pyr H), 7.83 (d, *J* = 8.7 Hz, 2H, Ar H), 7.28 – 7.05 (m, 2H, Ar H), 3.88 (s, 3H, CH_3). ^{13}C NMR (150 MHz, DMSO-d_6): δ 159.5, 150.8, 145.8, 129.8, 121.9, 121.4, 115.0, 55.6. HRMS (ESI), *m/z* $[M+H]^+$: calculated for $C_{14}H_{13}N_4O^+$ 253.1084; found 253.1146.

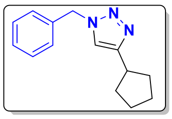

1-benzyl-4-cyclopentyl-1*H*-1,2,3-triazole (23). By following the General procedure, starting from benzyl azide (266 mg, 2.0 mmol, 1.0 equiv), ethynylcyclopentane (207 mg, 2.2 mmol, 1.1 equiv), $CuSO_4 \cdot 5H_2O$ (50 mg, 0.2 mmol, 0.1 equiv) and sodium ascorbate (40 mmol, 0.2 mmol, 0.1 equiv) in Cyrene (2 mL), compound **23** was obtained with 89% yield (407 mg) as a yellow solid. ^{1}H NMR (400 MHz, DMSO-*d*$_6$): δ 7.90 (s, 1H, Triazole H), 7.41 – 7.24 (m, 5H, Ar H), 5.51 (s, 2H, CH_2), 3.12 – 3.01 (m, 1H, CH), 2.03 – 1.84 (m, 2H, Aliphatic H), 1.73 – 1.52 (m, 6H, Aliphatic H). ^{13}C NMR (100 MHz, DMSO-*d*$_6$): δ 151.5, 136.3, 128.7, 128.0, 127.8, 121.0, 52.6, 36.2, 32.8, 24.6. HRMS (ESI), *m/z* $[M+H]^+$: calculated for $C_{14}H_{18}N_3^+$ 228.1495; found 228.1574.

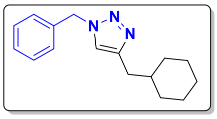

1-benzyl-4-(cyclohexylmethyl)-1*H*-1,2,3-triazole (24). By following the General procedure, starting from benzyl azide (266 mg, 2.0 mmol, 1.0 equiv), prop-2-yn-1-ylcyclohexane (269 mg, 2.2 mmol, 1.1 equiv), $CuSO_4 \cdot 5H_2O$ (50 mg, 0.2 mmol, 0.1 equiv) and sodium ascorbate (40 mmol, 0.2 mmol, 0.1 equiv) in Cyrene (2 mL), compound **24** was obtained with 79% yield (405 mg) as a yellow solid. ^{1}H NMR (400 MHz, DMSO-*d*$_6$): δ 7.87 (s, 1H, Triazole H), 7.40 – 7.30 (m, 3H, Ar H), 7.30 – 7.25 (m, 2H, Ar H), 5.53 (s, 2H, CH_2N), 2.48 (d, J = 7.0 Hz, 2H, CH_2), 1.70 – 1.57 (m, 5H, Aliphatic H), 1.29 – 1.08 (m, 4H, Aliphatic H), 0.95 – 0.85 (m, 2H, Aliphatic H). ^{13}C NMR (100 MHz, DMSO-*d*$_6$): δ 146.3, 136.8, 129.2, 128.5, 128.2, 123.0, 53.1, 38.0, 33.2, 32.9, 26.4, 26.1. HRMS (ESI), *m/z* $[M+H]^+$: calculated for $C_{16}H_{22}N_3^+$ 256.1808; found 256.1896.

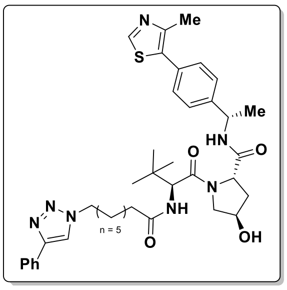

(2*S*,4*R*)-1-((*S*)-3,3-dimethyl-2-(8-(4-phenyl-1*H*-1,2,3-triazol-1-yl)octanamido)butanoyl)-4-hydroxy-*N*-((*S*)-1-(4-(4-methylthiazol-5-yl)phenyl)ethyl)pyrrolidine-2-carboxamide (25). By following the General procedure, starting from VHL-azide (324 mg, 0.5 mmol, 1.0 equiv), phenylacetylene (56 mg, 0.55 mmol, 1.1 equiv), $CuSO_4 \cdot 5H_2O$ (13 mg, 0.05 mmol, 0.4 equiv) and sodium ascorbate (10 mg, 0.05 mmol, 0.4 equiv) in Cyrene (4 mL), compound **25** was obtained with 74% yield (277 mg) as a white solid, after 24 h of reaction time. ^{1}H NMR (400 MHz, DMSO-*d*$_6$): δ 9.04 (s, 1H, Thiazole H), 8.57 (s, 1H, Triazole H), 8.35 (d, J = 7.8 Hz, 1H, N – H), 7.83 (d, J = 7.6 Hz, 2H, Ar H), 7.76 (d, J = 9.3 Hz, 1H, NH), 7.50 – 7.26 (m, 7H, Ar H), 5.08 (d, J = 3.5 Hz, 1H, Proline H), 5.04 (s, 1H, OH), 4.91 (p, J = 6.9 Hz, 1H, C*H* – Me), 4.75 (t, J = 4.8 Hz, 1H, Proline H), 4.51 (d, J = 9.3 Hz, 1H, C*H* – *t*Bu), 4.40 (dt, J = 11.3, 7.5 Hz, 2H, CH_2 triazole), 4.31 – 4.23 (m, 1H, Proline H), 4.10 (d, J = 7.4 Hz, 1H, Proline H), 3.80 (ddd, J = 7.1, 5.2, 1.5 Hz, 1H, Proline H), 3.60 (d, J = 4.1 Hz, 2H, CH_2 linker), 2.70 (ddd, J = 16.6, 11.3, 8.8 Hz, 1H, Proline H), 2.45 (s, 3H, Thiazole CH_3), 2.29 – 2.18 (m, 2H, CH_2 linker), 2.10 (dt, J = 11.4, 5.6 Hz, 2H, CH_2 linker), 2.06 – 1.95 (m, 2H, CH_2 linker), 1.92 – 1.76 (m, 2H, CH_2 linker), 1.47 (dq, J = 14.9, 7.9, 7.5 Hz, 2H, CH_2 linker), 1.37 (d, J = 6.9 Hz, 3H, CH – *Me*), , 0.92 (s, 9H, –*t*Bu). ^{13}C NMR (100 MHz, DMSO-*d*$_6$): δ 171.9, 170.5, 169.5, 146.2, 144.6, 144.3, 130.8, 129.7, 128.8 (2C), 127.7, 126.3, 125.0 (2C), 121.2, 100.4, 72.7, 68.7, 66.9, 58.5, 56.3, 56.2, 49.4, 47.6, 37.7, 35.1, 34.7, 30.7, 29.5, 29.2, 28.4, 28.0, 26.4, 25.7, 25.2, 22.3. HRMS (ESI), *m/z* $[M+H]^+$: calculated for $C_{39}H_{51}N_7NaO_4S^+$ 736.3615; found 736.3699. HPLC rt: 18.3 min. $[\alpha]_D$= − 67,7°

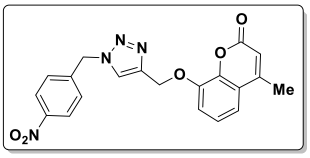

4-Methyl-8-((1-(4-nitrobenzyl)-1*H*-1,2,3-triazol-4-yl)methoxy)-2*H*-chromen-2-one (26).[16] By following the General procedure, starting from 1-(azidomethyl)-4-nitrobenzene (254 mg, 1.42 mmol, 1.15 equiv), 4-methyl-8-(prop-2-yn-1-yloxy)-2*H*-chromen-2-one (276 mg, 1.29 mmol, 1.0 equiv), $CuSO_4 \cdot 5H_2O$ (32 mg, 0.13 mmol, 0.1 equiv) and sodium ascorbate (25 mg, 0.13 mmol, 0.1 equiv) in Cyrene (4 mL) at 40 °C, compound **26** was obtained with 67% yield (340 mg) as a white solid. ^{1}H NMR (400 MHz, DMSO-*d*$_6$): δ 8.40 (s, 1H, Triazole H), 8.23 (d, J = 8.8 Hz, 2H, Ar H), 7.69 (d, J = 8.7 Hz, 1H, Ar H), 7.54 (d, J = 8.8 Hz, 2H, Ar H), 7.14 (d, J = 2.3 Hz, 1H, Ar H), 7.03 (dd, J = 8.7, 2.3 Hz, 1H, Ar H), 6.22 (s, 1H, Ar H), 5.81 (s, 2H, CH_2O), 5.29 (s, 2H, CH_2N), 2.39 (s, 3H, CH_3). ^{13}C NMR (100 MHz, DMSO-*d*$_6$): δ 160.7, 159.8, 154.4, 153.1, 147.0, 143.1, 142.3, 128.8, 126.3, 125.2, 123.7, 113.2, 112.4, 111.1, 101.4, 61.4, 51.7, 17.9. HRMS (ESI), *m/z* $[M+H]^+$: calculated for $C_{20}H_{17}N_4O_5^+$ 393.1193; found 393.1201.

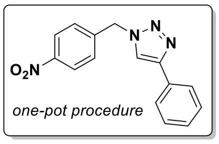

1-(4-Nitrobenzyl)-4-phenyl-1*H*-1,2,3-triazole (9). A mixture of 4-nitrobenzyl bromide (972 mg, 4.5 mmol, 1.0 equiv), phenylacetylene (552 mg, 5.4 mmol, 1.2 equiv), sodium azide (438 mg, 6.75 mmol, 1.5 equiv), $CuSO_4 \cdot 5H_2O$ (111 mg, 0.45 mmol, 0.1 equiv) and sodium ascorbate (90 mg, 0.45 mmol, 0.1 equiv) in Cyrene (7 mL) was left stirring at room temperature for 24 h. After complete conversion of the starting material by TLC analysis,

the reaction mixture was poured into ice-water and the precipitate was collected by filtration and washed with water to afford the pure **9** with 83% yield (1.05 g) as a brown solid. *Spectral and analytical characterizations are in agreement with the ones reported for* ***9.***

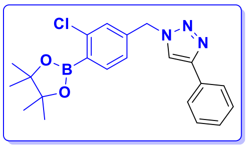

1-(3-chloro-4-(4,4,5,5-tetramethyl-1,3,2-dioxaborolan-2-yl)benzyl)-4-phenyl-1*H*-1,2,3-triazole (27). By following the General procedure, starting from 2-(4-(azidomethyl)-2-chlorophenyl)-4,4,5,5-tetramethyl-1,3,2-dioxaborolane (461 mg, 1.57 mmol, 1.0 equiv), phenylacetylene (177 mg, 1.73 mmol, 1.1 equiv), $CuSO_4 \cdot 5H_2O$ (40 mg, 0.16 mmol, 0.1 equiv) and sodium ascorbate (32 mg, 0.16 mmol, 0.1 equiv) in Cyrene (2 mL), compound **27** was obtained with 56% yield (348 mg) as a whitish solid. ^{1}H NMR (600 MHz, Chloroform-*d*): δ 7.80 (d, *J* = 7.1 Hz, 2H, Ar H), 7.70 (d, *J* = 7.6 Hz, 1H, Ar H), 7.65 (s, 1H, Triazole H), 7.41 (t, *J* = 7.6 Hz, 2H, Ar H), 7.33 (t, *J* = 7.1 Hz, 1H, Ar H), 7.30 (d, *J* = 1.3 Hz, 1H, Ar H), 7.16 (dd, *J* = 7.6, 1.3 Hz, 1H, Ar H), 5.54 (s, 2H, CH_2), 1.36 (s, 12H, CH_3). ^{13}C NMR (150 MHz, Chloroform-*d*): δ 148.6, 140.6, 138.3, 137.4, 130.3, 129.0, 128.9, 128.5, 125.9, 125.5, 119.7, 84.5, 53.7, 24.9, 1 quaternary carbon missing. HRMS (ESI), *m/z* [M+H]$^+$: calculated for $C_{21}H_{24}BClN_3O_2^+$ 396.1645; found 396.1617.

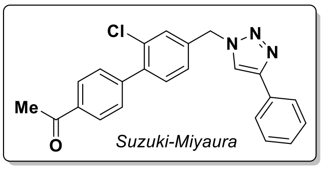

1-(2'-chloro-4'-((4-phenyl-1*H*-1,2,3-triazol-1-yl)methyl)-[1,1'-biphenyl]-4-yl)ethan-1-one (28). A mixture of **27** (348 mg, 0.88 mmol. 1.0 equiv), 4-bromoacetophenone (201 mg, 1.01 mmol, 1.15 equiv), $Pd(PPh_3)_4$ (104 mg, 0.09 mmol, 0.1 equiv) and Na_2CO_3 (187 mg, 1.76 mmol, 2.0 equiv) in Cyrene:water (4 mL, 3:1, *v/v*) was left stirring at 130 °C for 24 h. The reaction was filtered through a short pad of celite, then the filtrate was extracted with EtOAc (3 x 15 mL). The organic layer was washed with brine (5 x 15 mL), dried with Na_2SO_4 and concentrated under reduced pressure. The crude was purified with column chromatography (*n*-hexane:EtOAc, 7:3 *v/v* as eluent) to afford the pure **28** (214 mg, 63%) as a yellow amorphous solid. ^{1}H NMR (400 MHz, Chloroform-*d*): δ 8.02 (d, *J* = 8.1 Hz, 2H, Ar H), 7.87 – 7.76 (m, 3H, Ar H), 7.51 (d, *J* = 8.2 Hz, 2H, Ar H), 7.45 (s, 1H, Triazole H), 7.41 (t, *J* = 7.5 Hz, 2H, Ar H), 7.34 (d, *J* = 7.8 Hz, 2H, Ar H), 7.28 (m, 1H, Ar H), 5.59 (s, 2H, CH_2), 2.63 (s, 3H, CH_3). ^{13}C NMR (100 MHz, Chloroform-*d*): δ 197.8, 148.5, 143.3, 139.9, 136.5, 136.2, 133.1, 131.9, 130.3, 129.7, 129.7, 129.0, 128.5, 128.3, 126.6, 125.9, 119.9, 53.3, 25.0. HRMS (ESI), *m/z* [M+H]$^+$: calculated for $C_{23}H_{19}ClN_3O^+$ 388.1211; found 388.1298.

^{1}H- and ^{13}C-NMR Spectra for all the Compounds

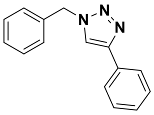

^{1}H NMR, 400 MHz, CDCl$_3$

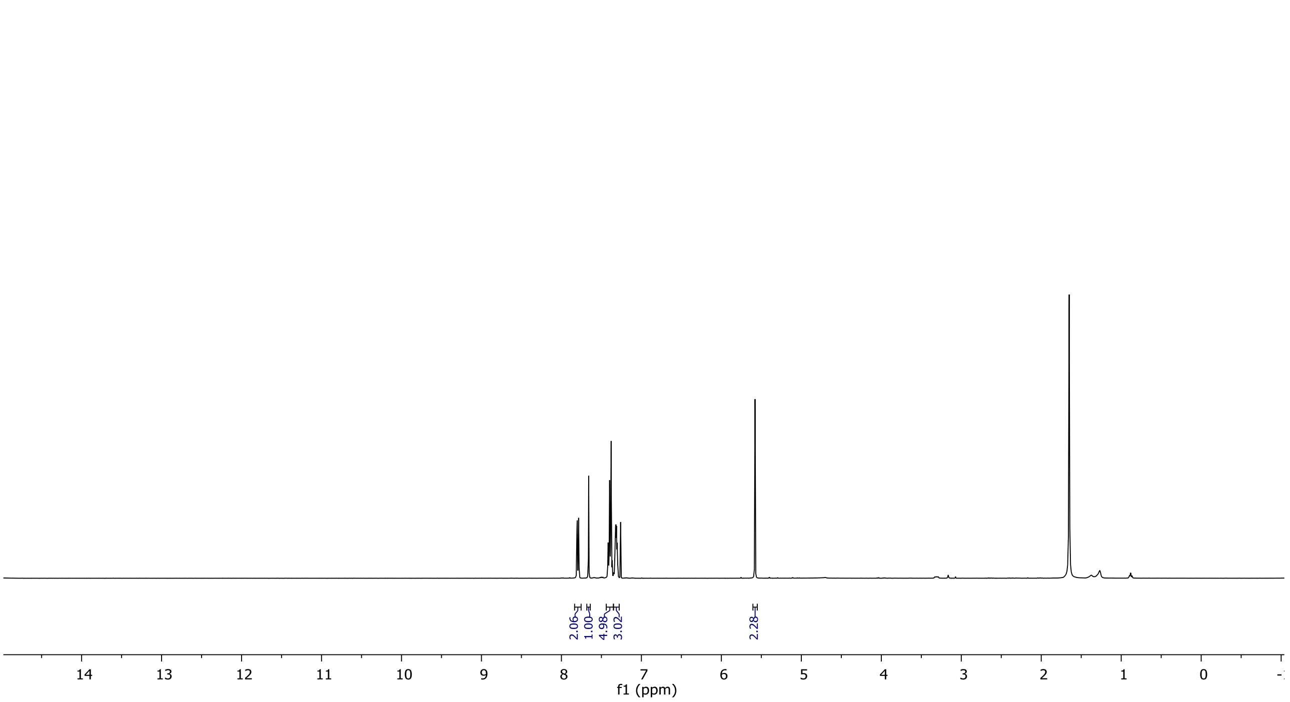

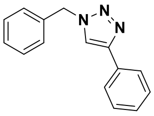

^{13}C NMR, 100 MHz, CDCl$_3$

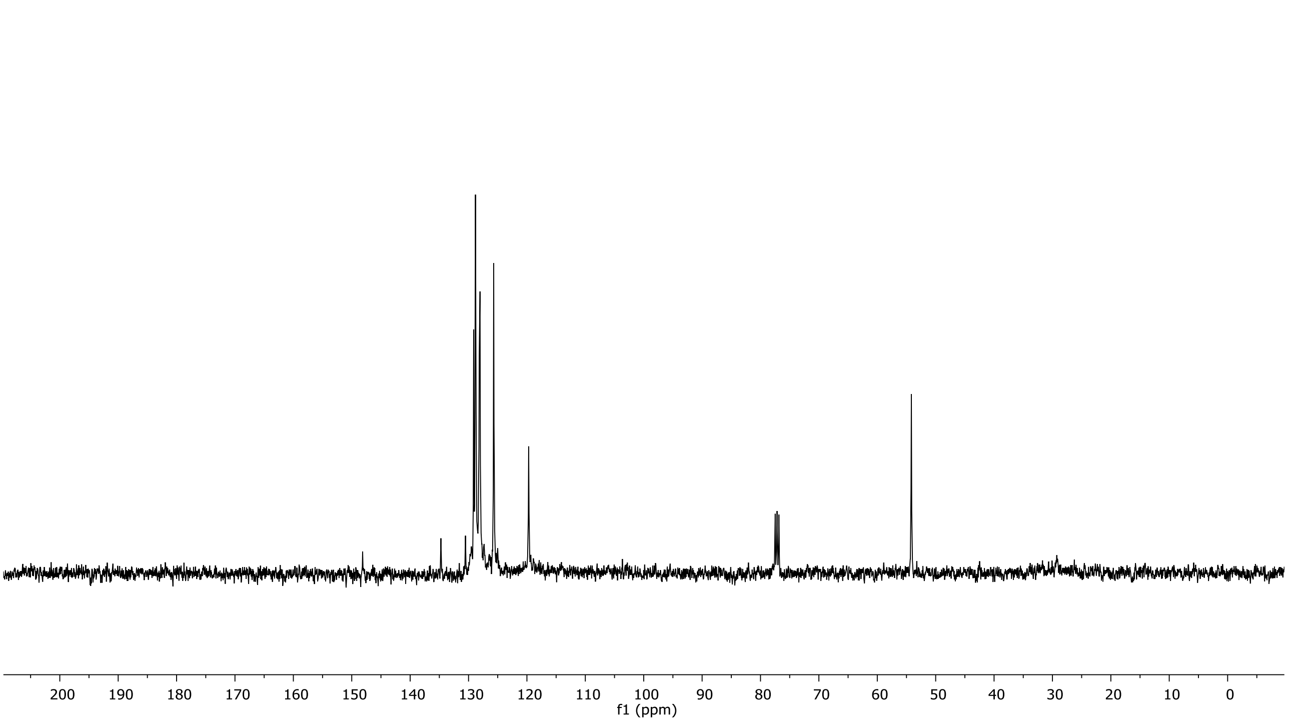

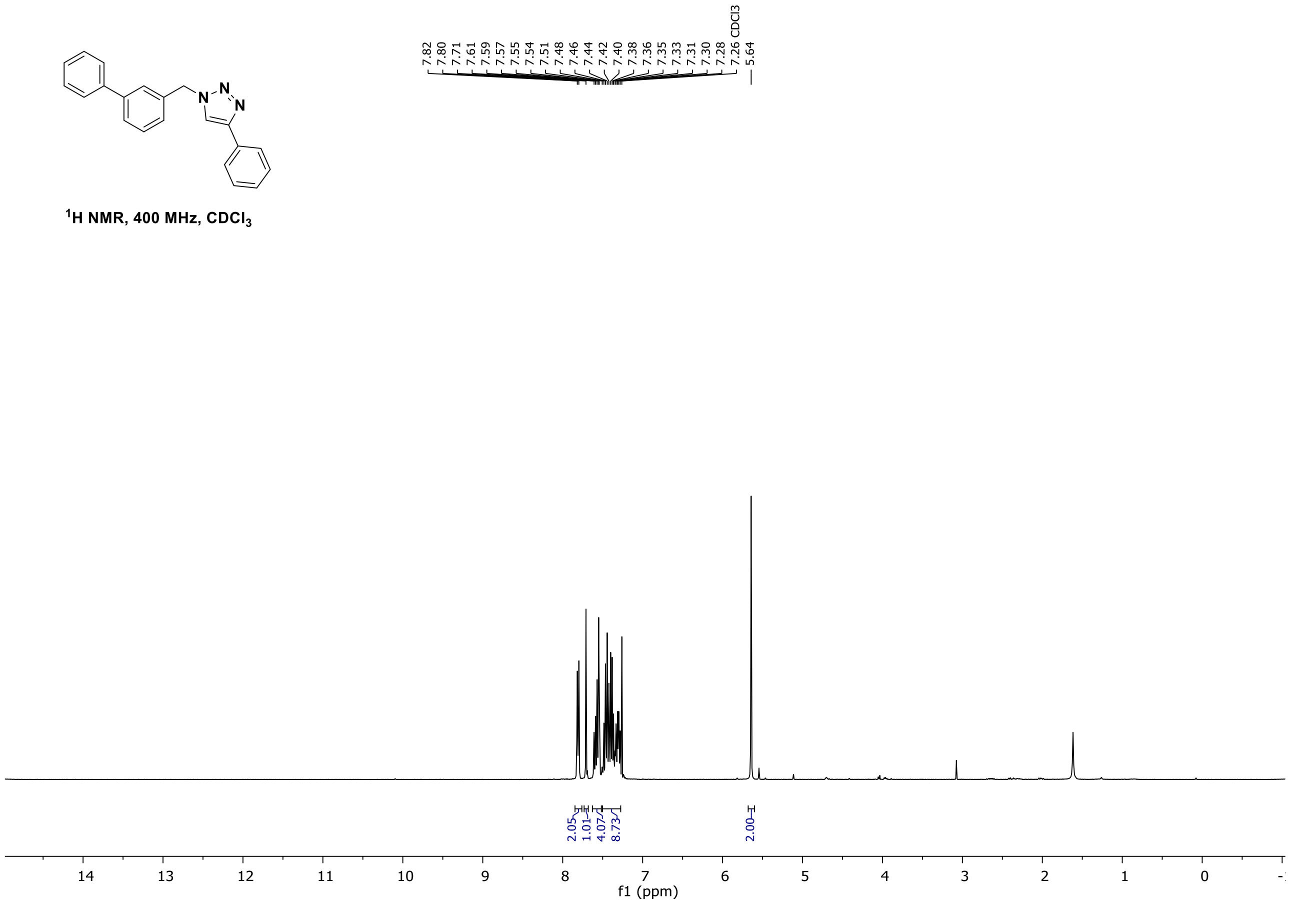

^{1}H NMR, 400 MHz, $CDCl_3$

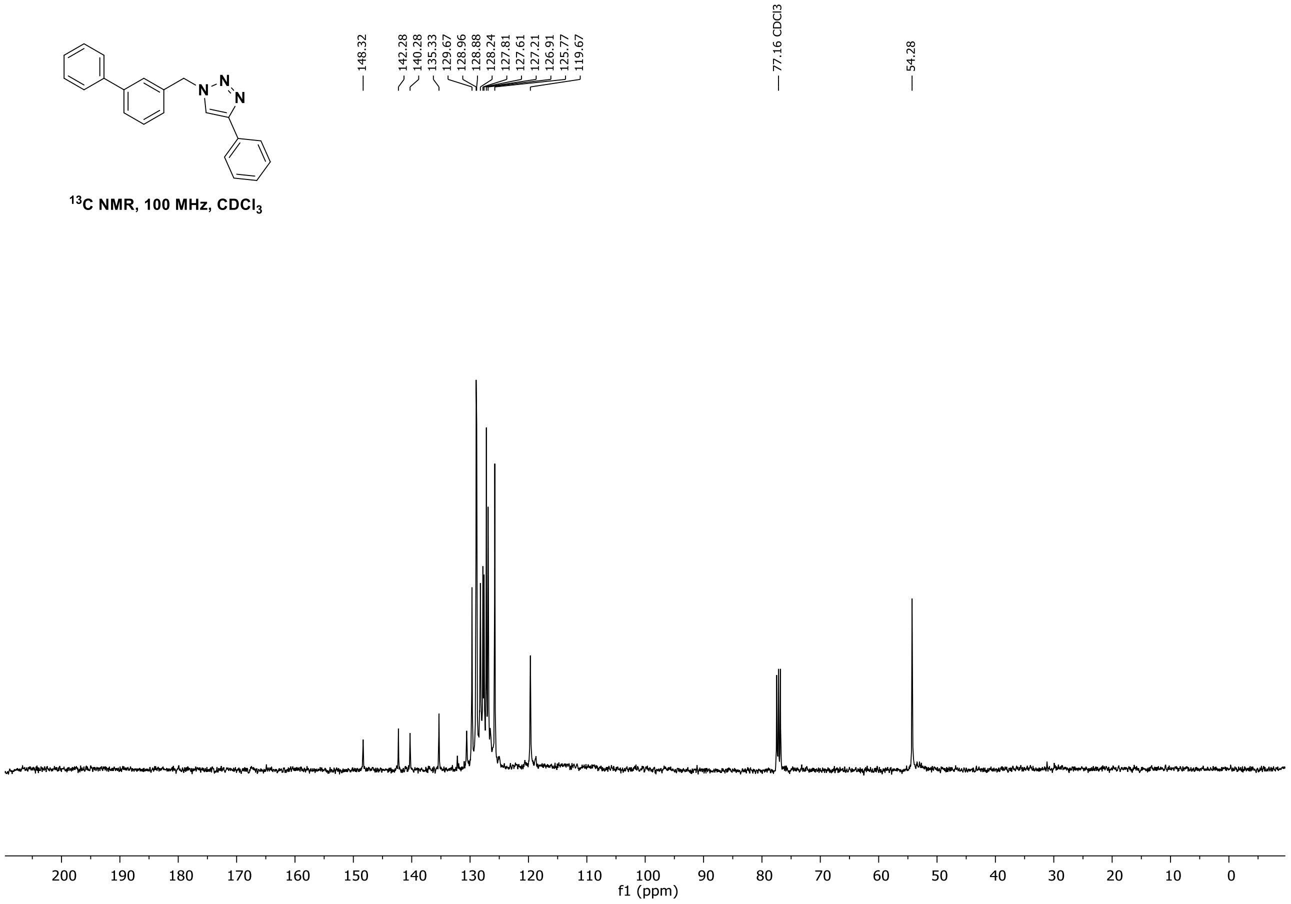

^{13}C NMR, 100 MHz, $CDCl_3$

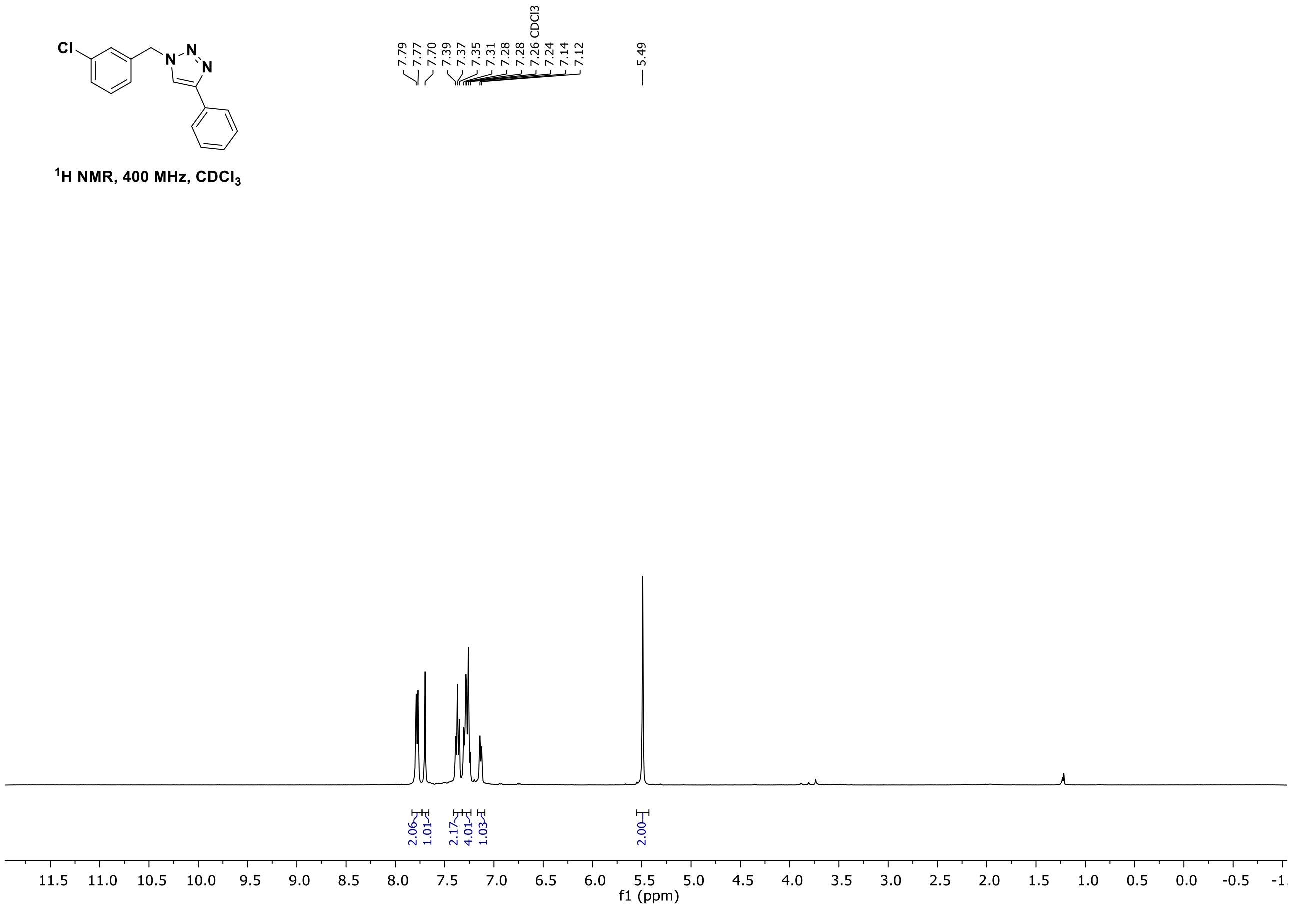

^{1}H NMR, 400 MHz, $CDCl_3$

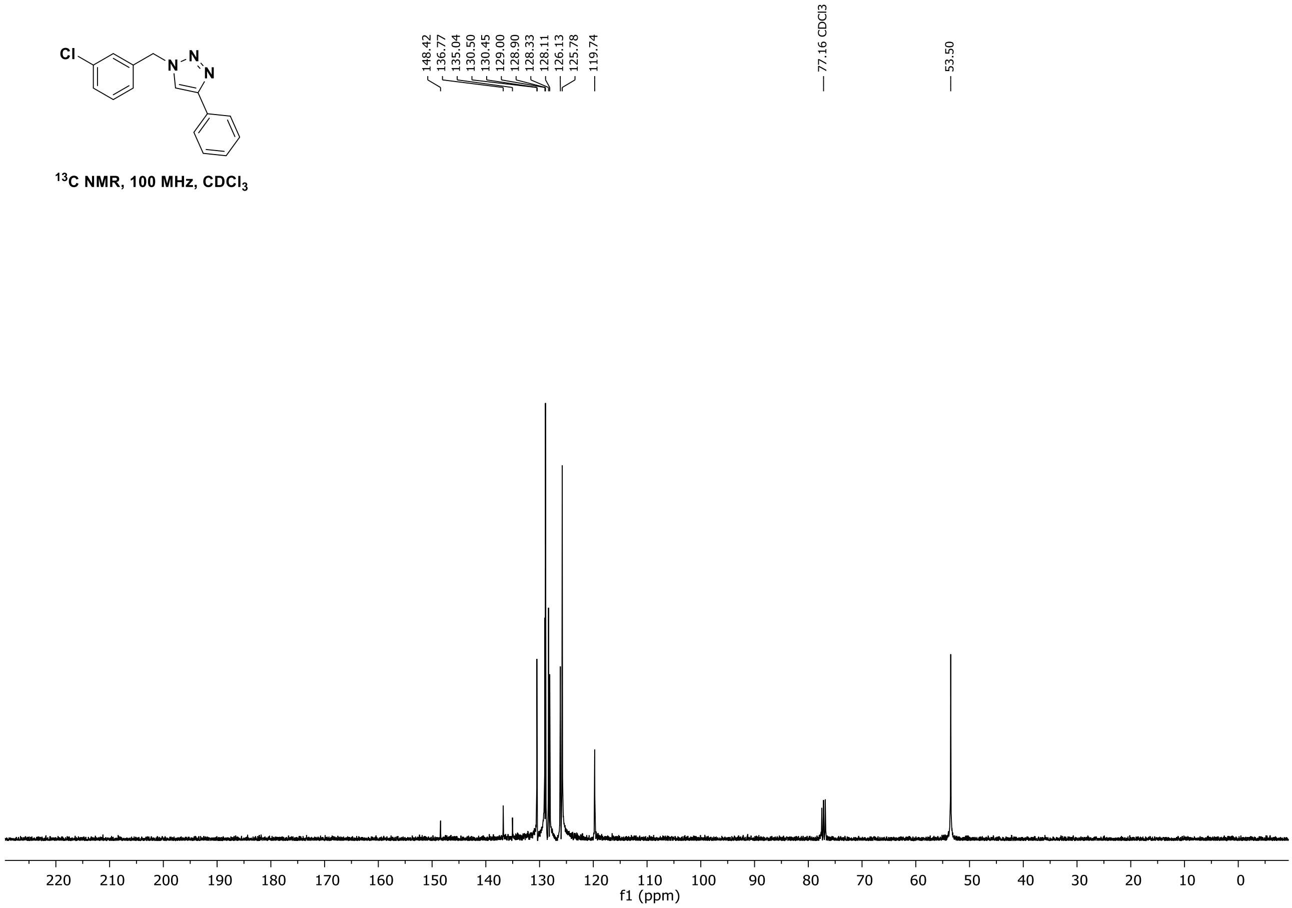

^{13}C NMR, 100 MHz, $CDCl_3$

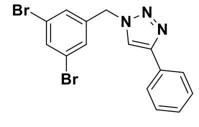

^{1}H NMR, 400 MHz, $CDCl_3$

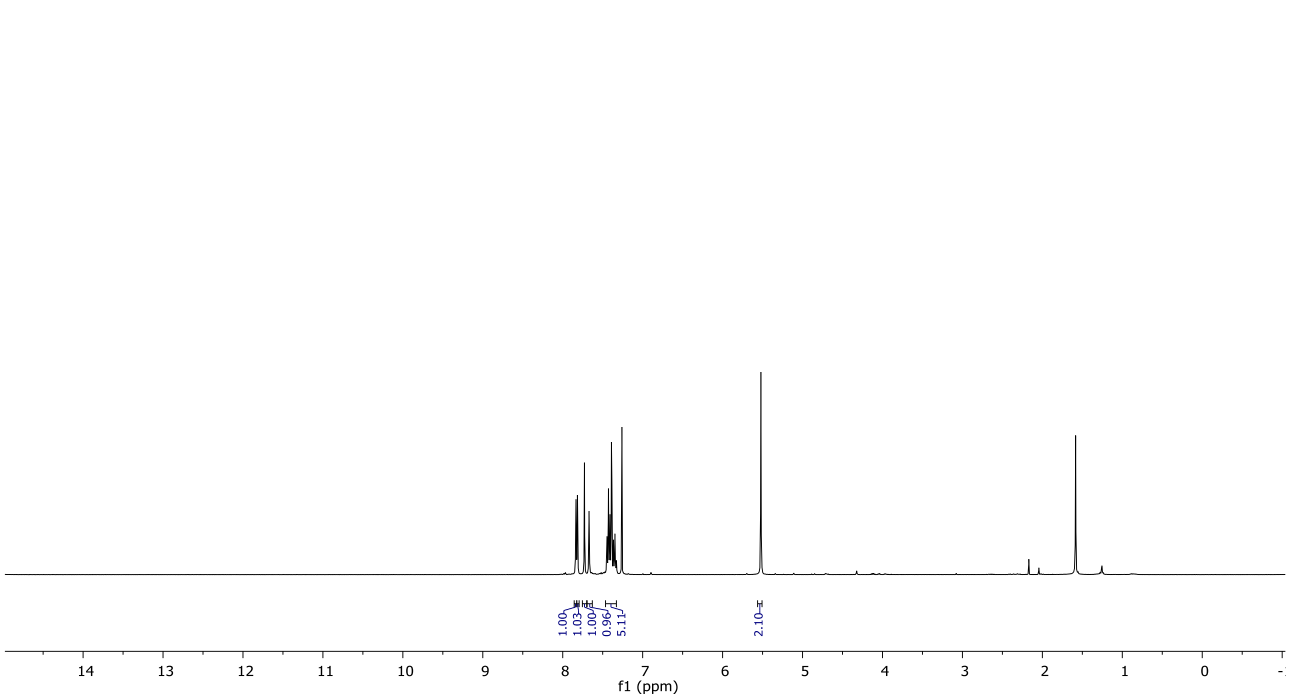

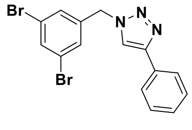

^{13}C NMR, 100 MHz, $CDCl_3$

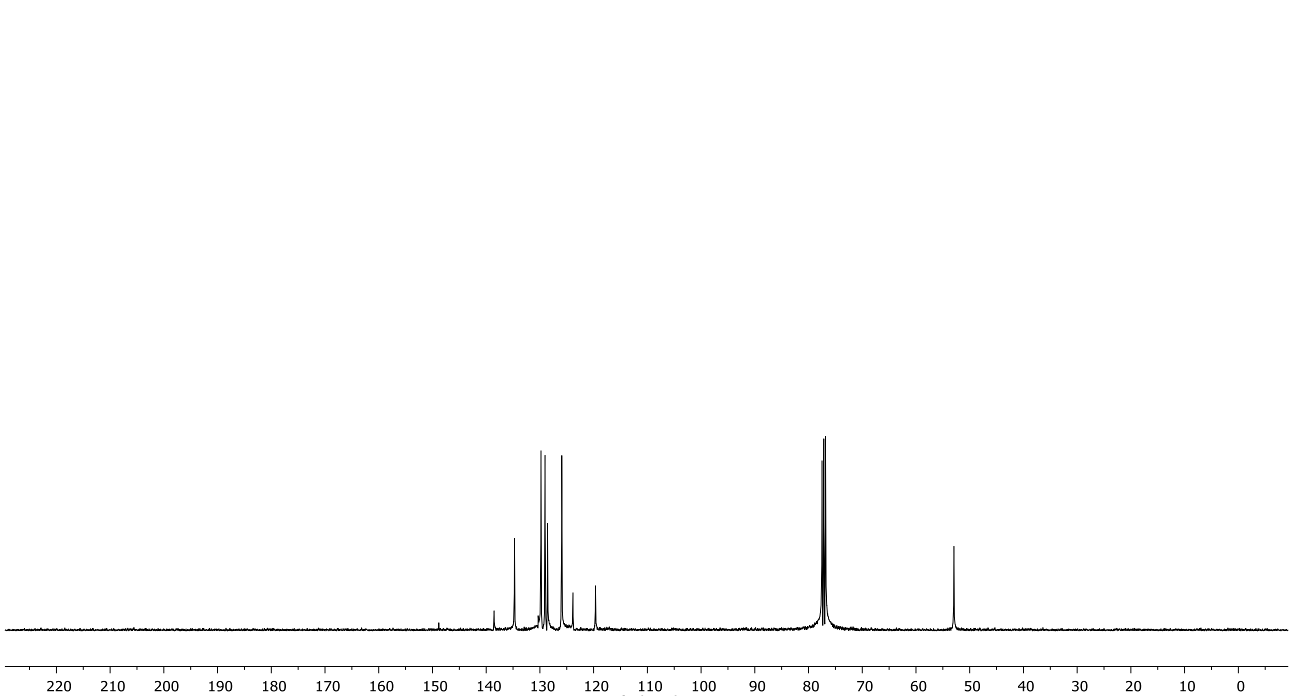

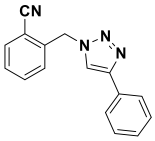

^1H NMR, 400 MHz, $CDCl_3$

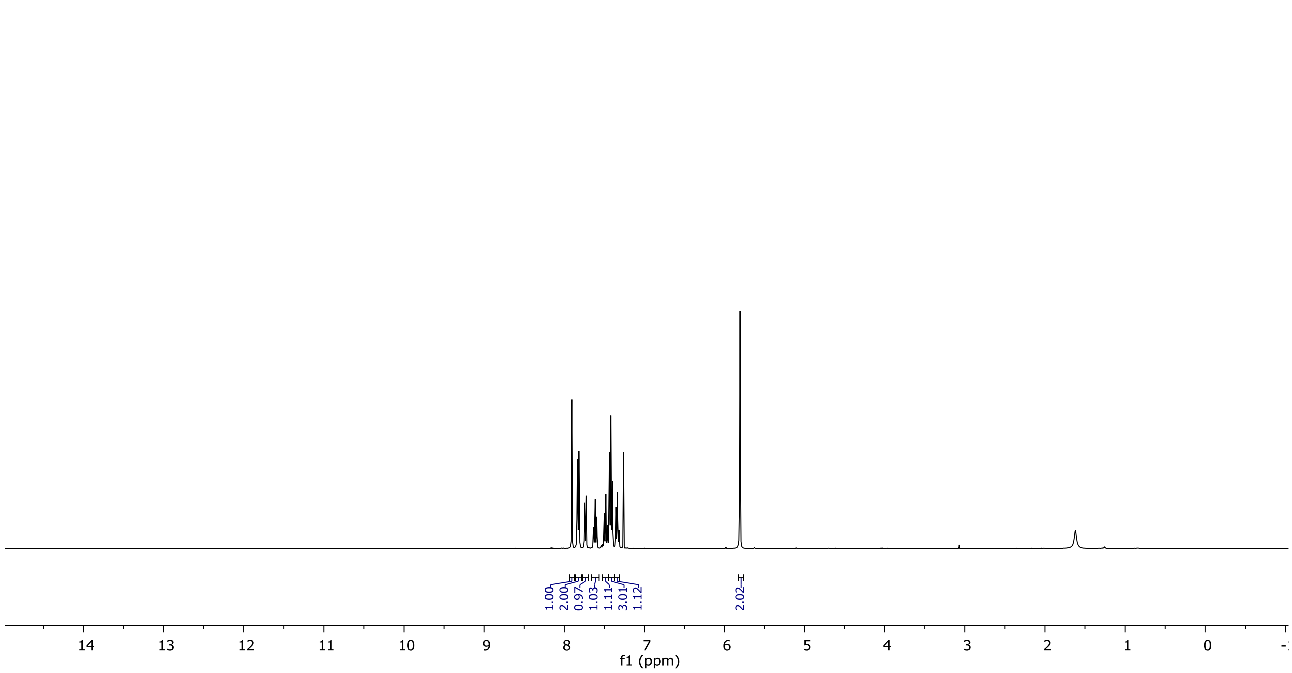

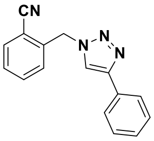

^{13}C NMR, 100 MHz, $CDCl_3$

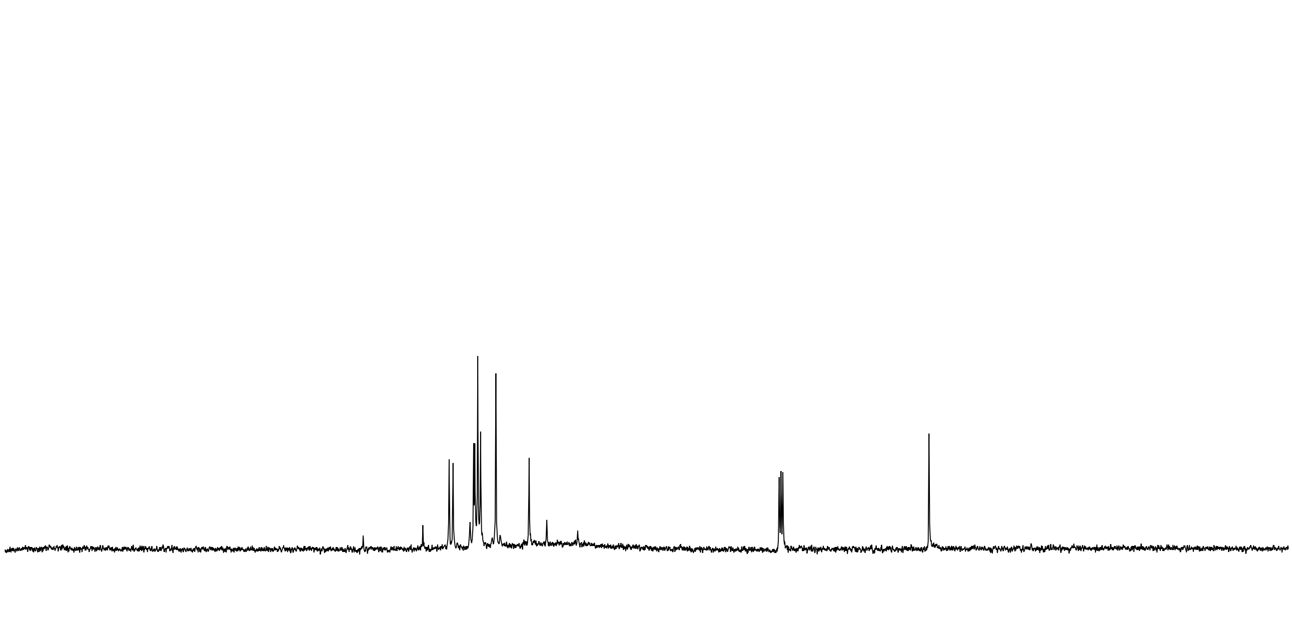

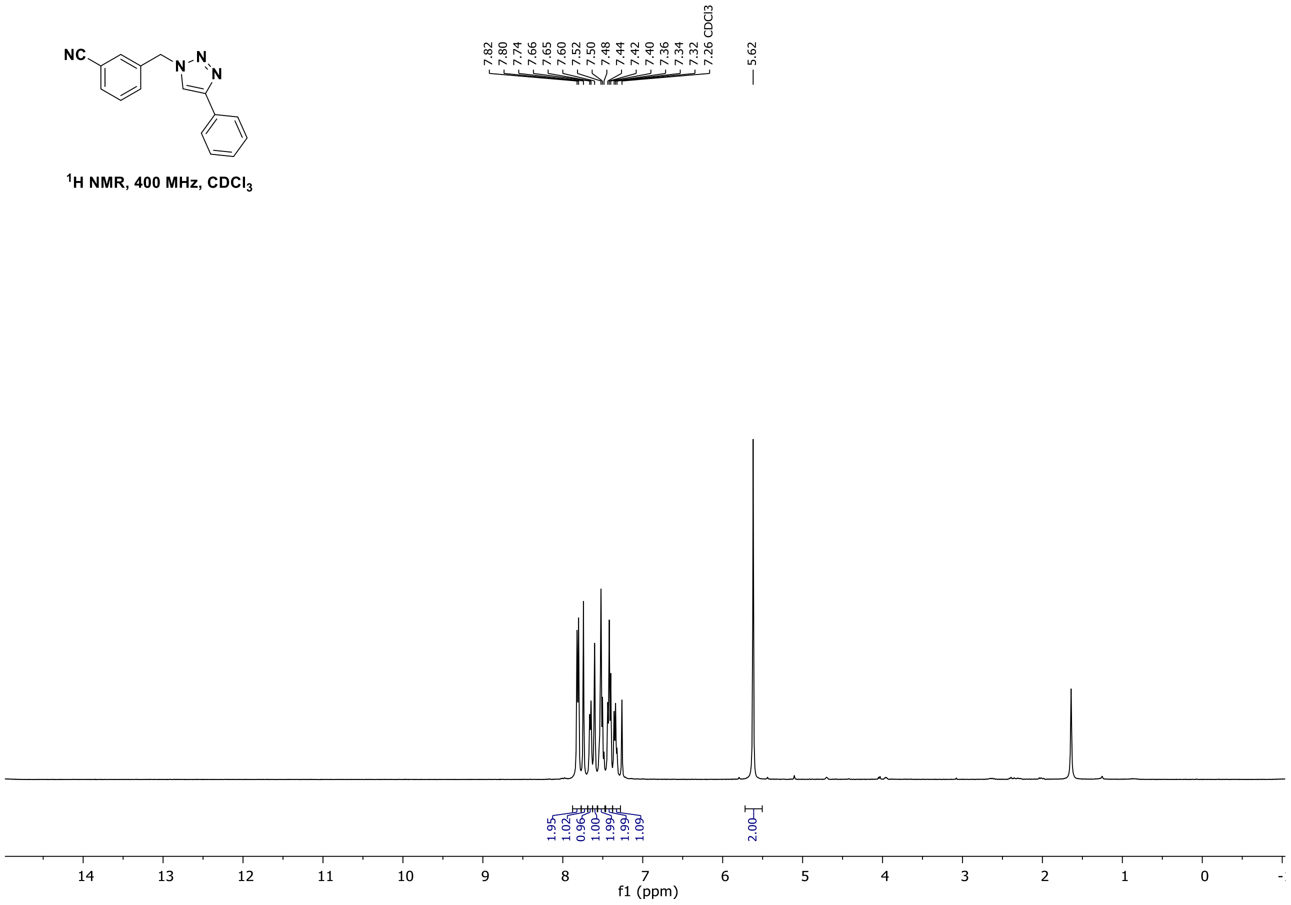

^{1}H NMR, 400 MHz, $CDCl_3$

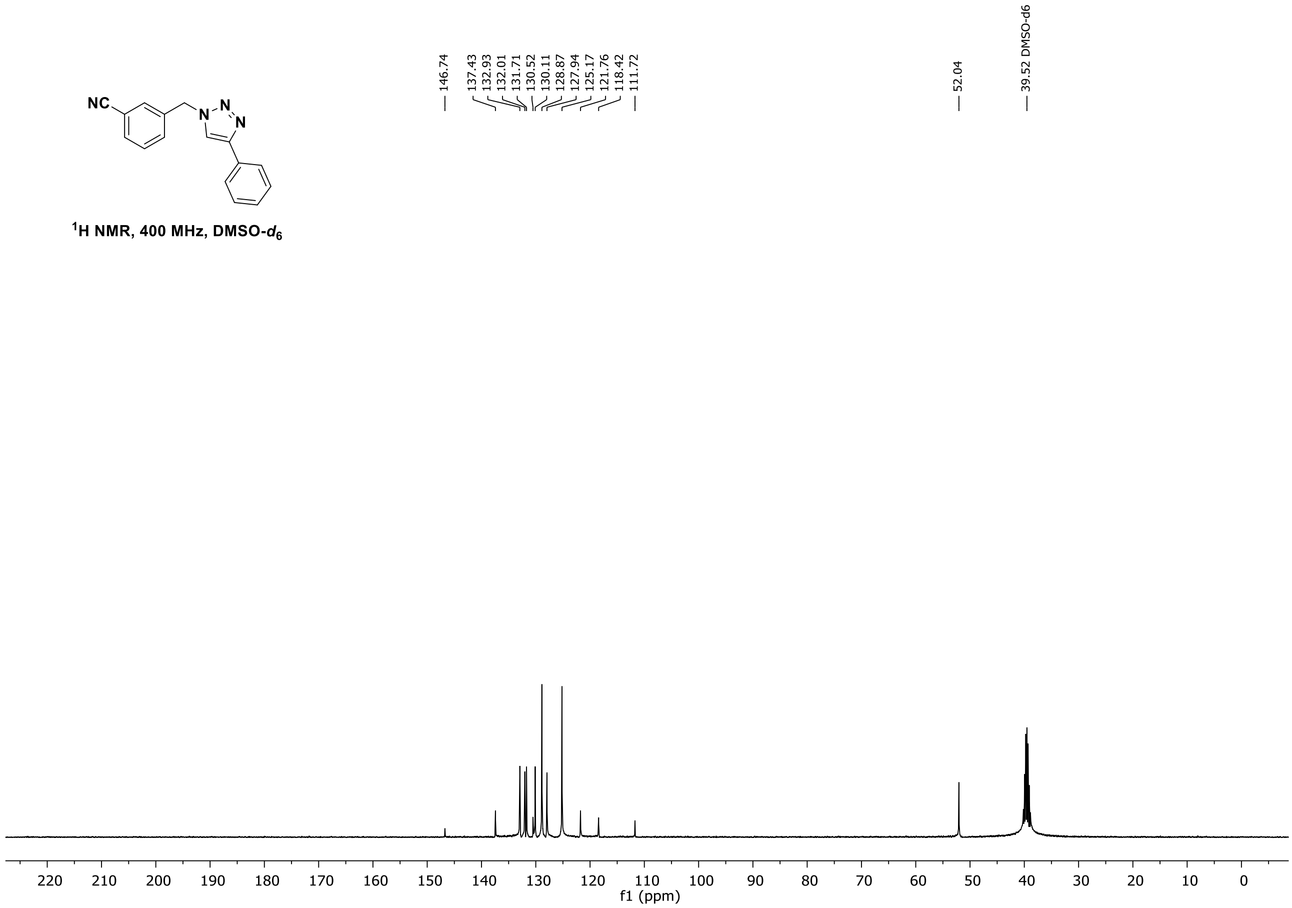

^{1}H NMR, 400 MHz, DMSO-d_6

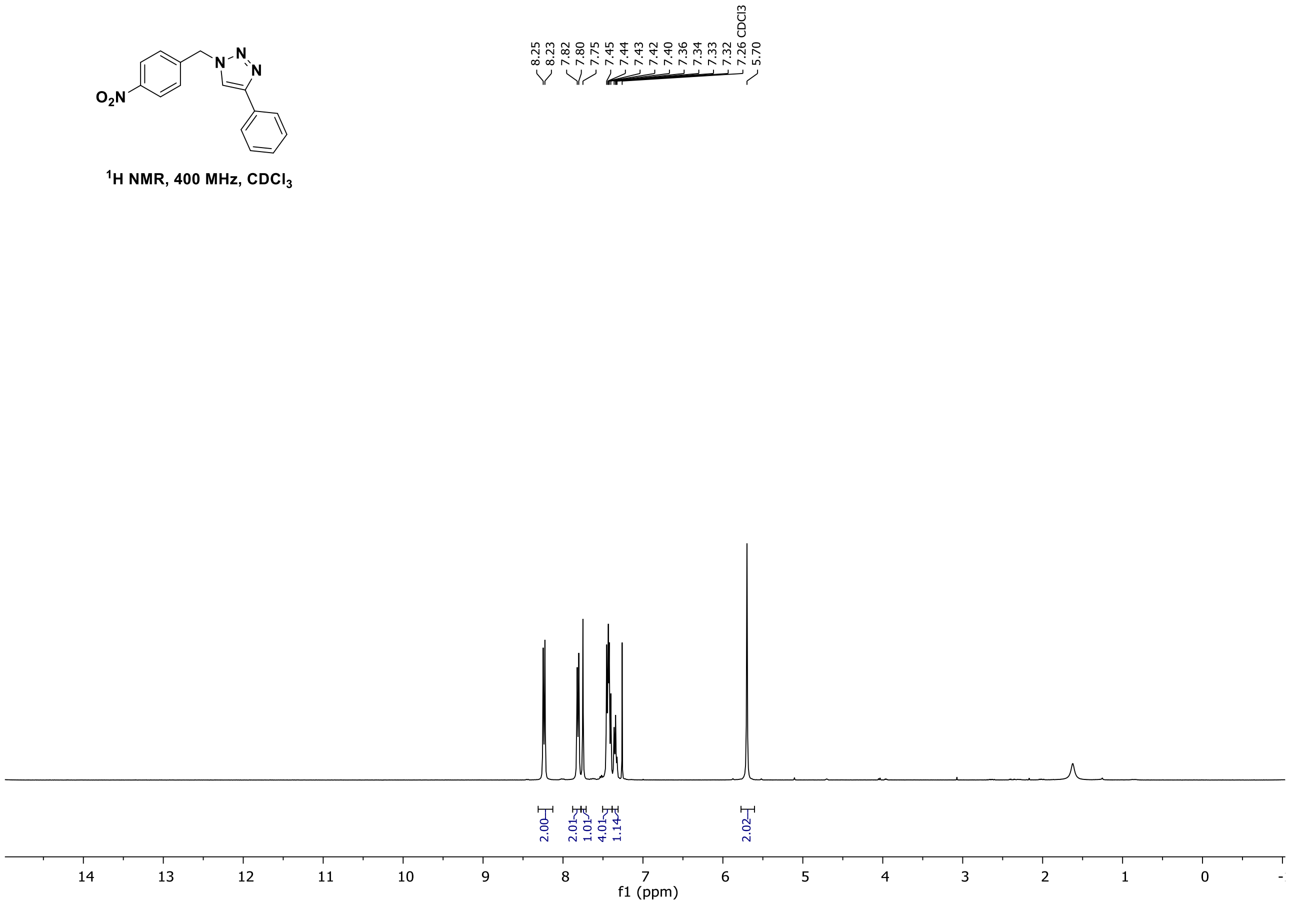

^1H NMR, 400 MHz, $CDCl_3$

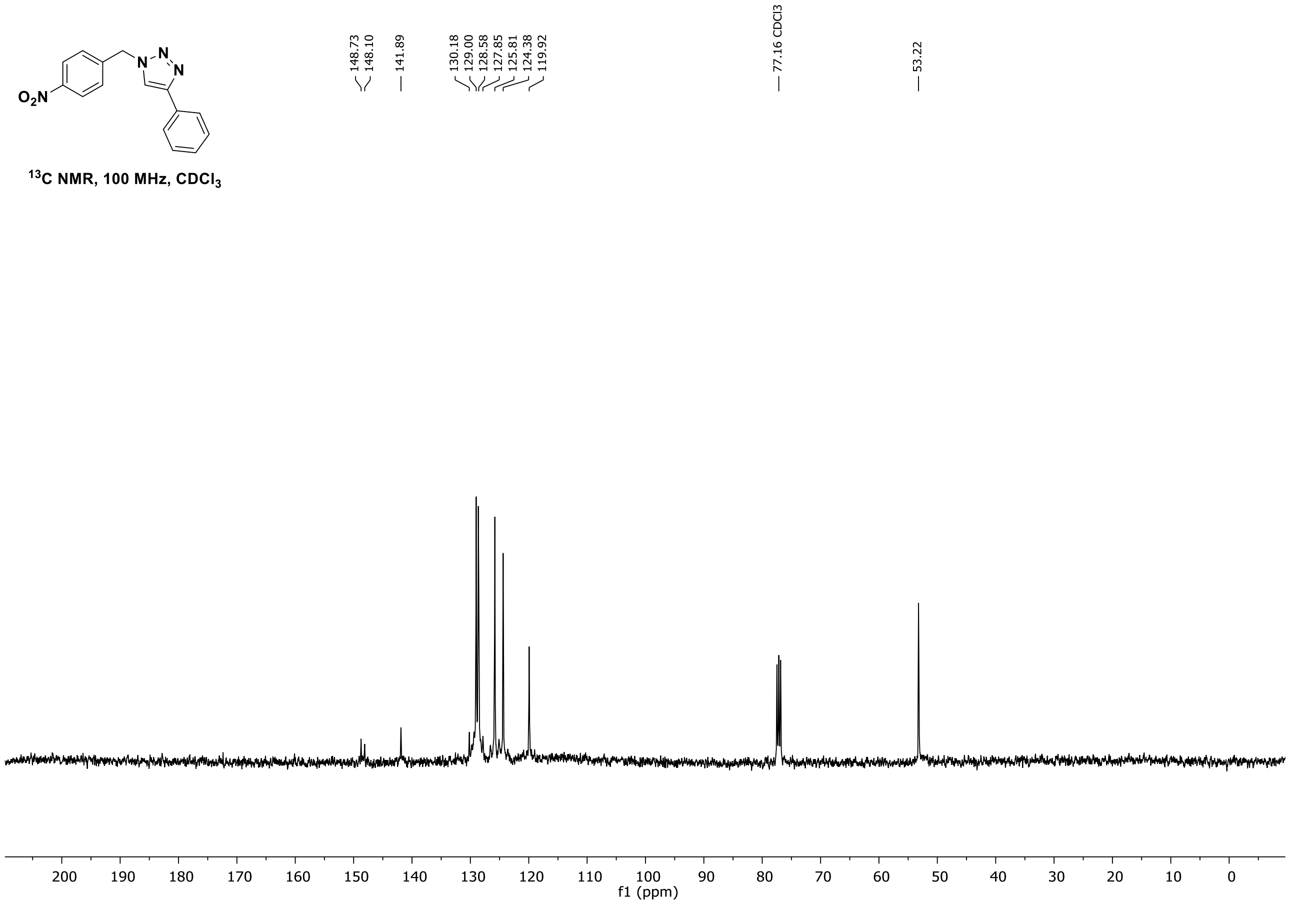

^{13}C NMR, 100 MHz, $CDCl_3$

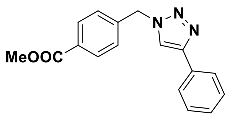

^{1}H NMR, 400 MHz, $CDCl_3$

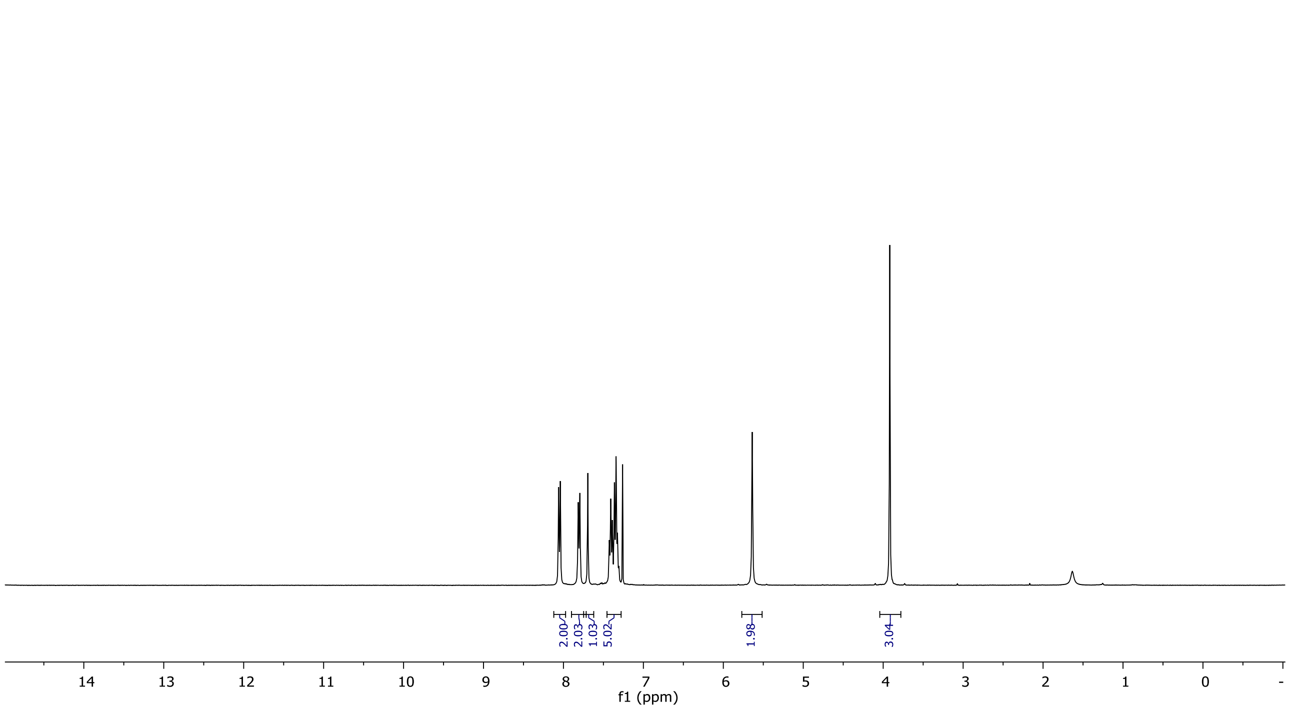

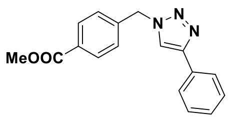

^{13}C NMR, 100 MHz, $CDCl_3$

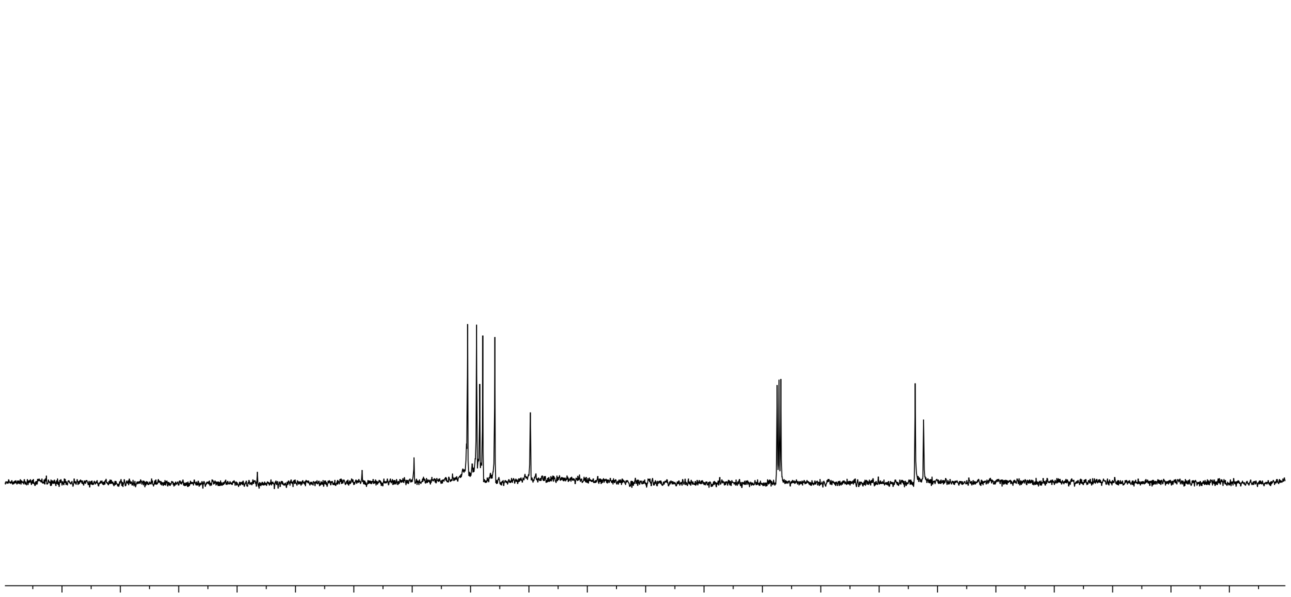

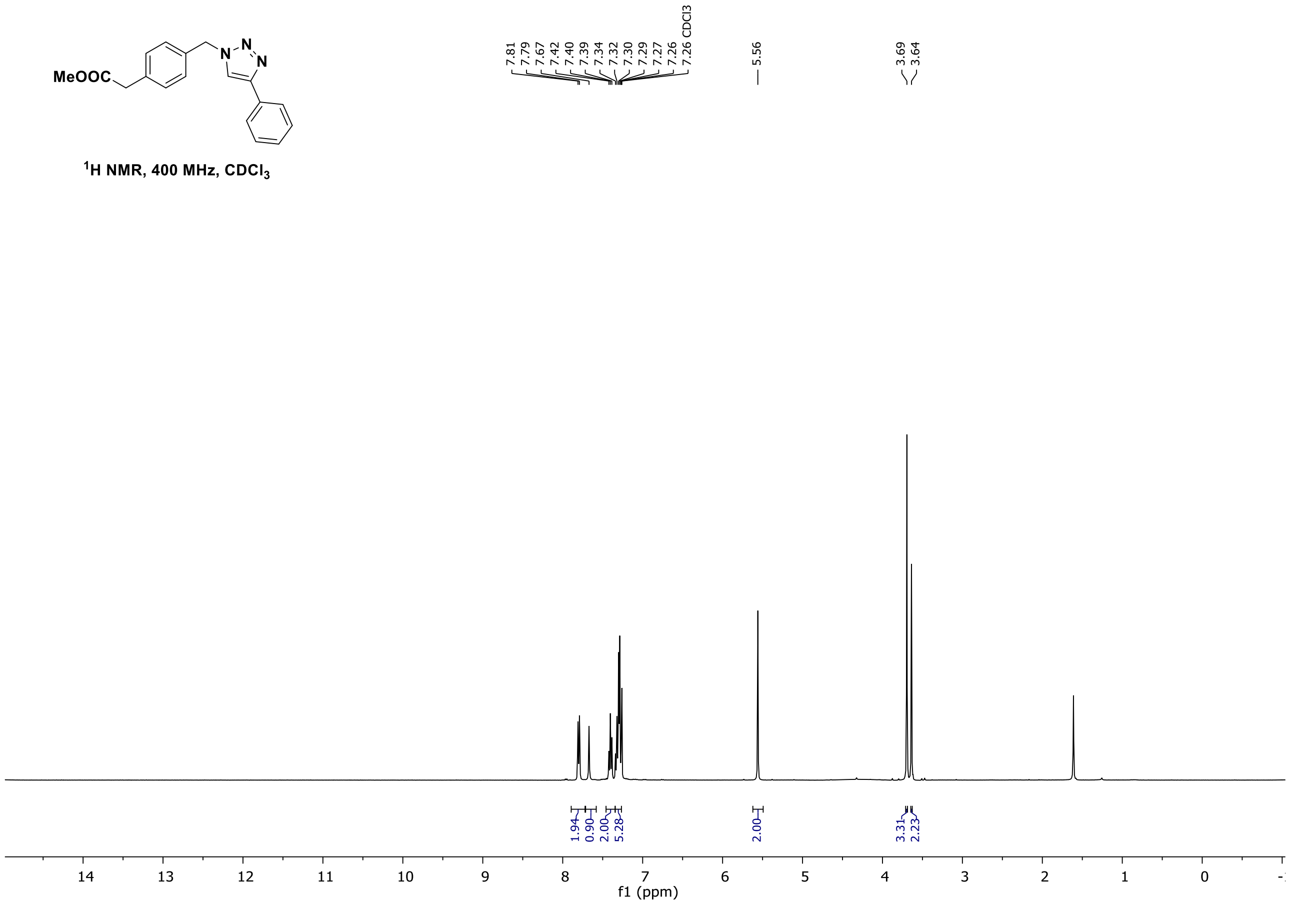

^{1}H NMR, 400 MHz, $CDCl_3$

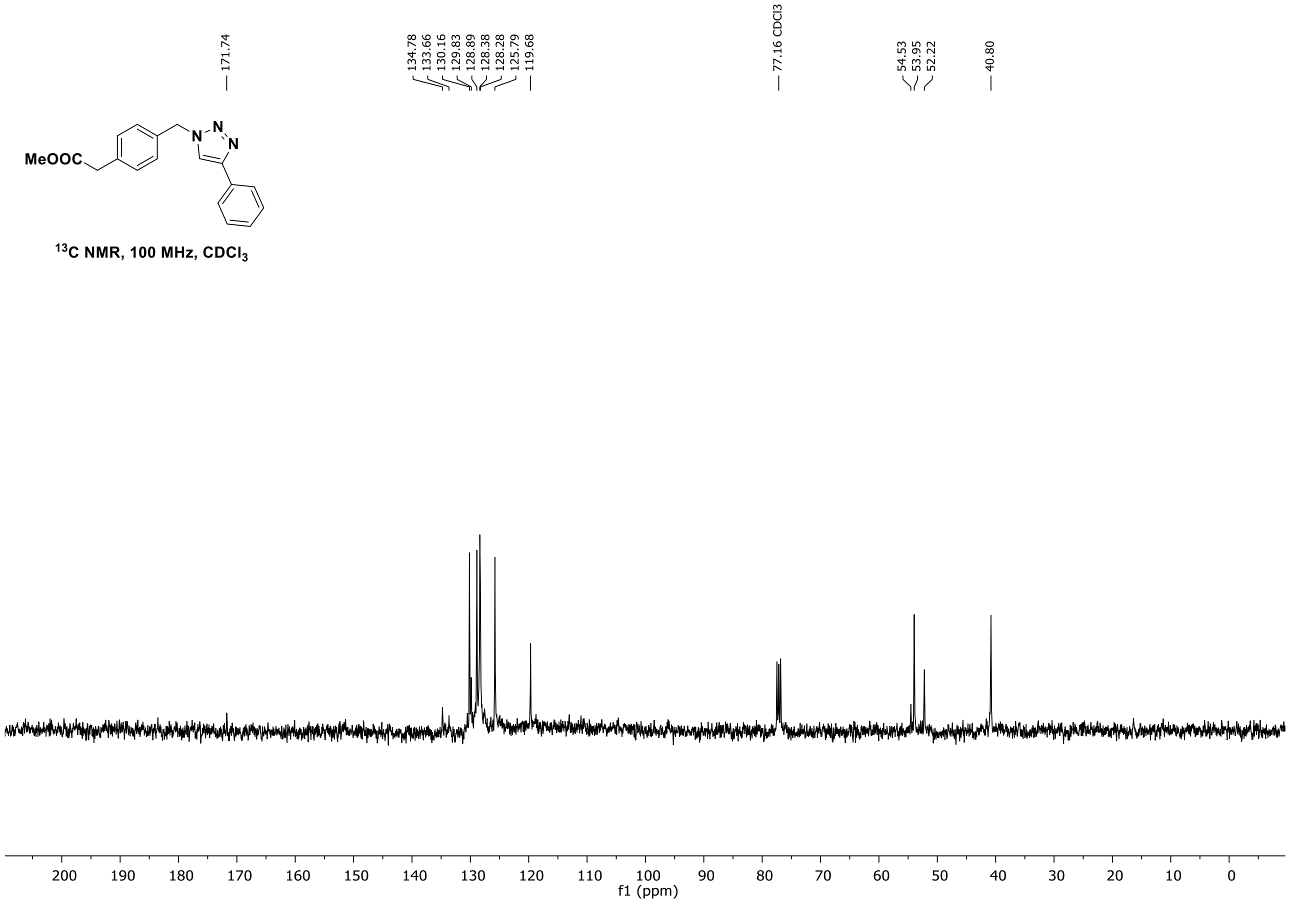

^{13}C NMR, 100 MHz, $CDCl_3$

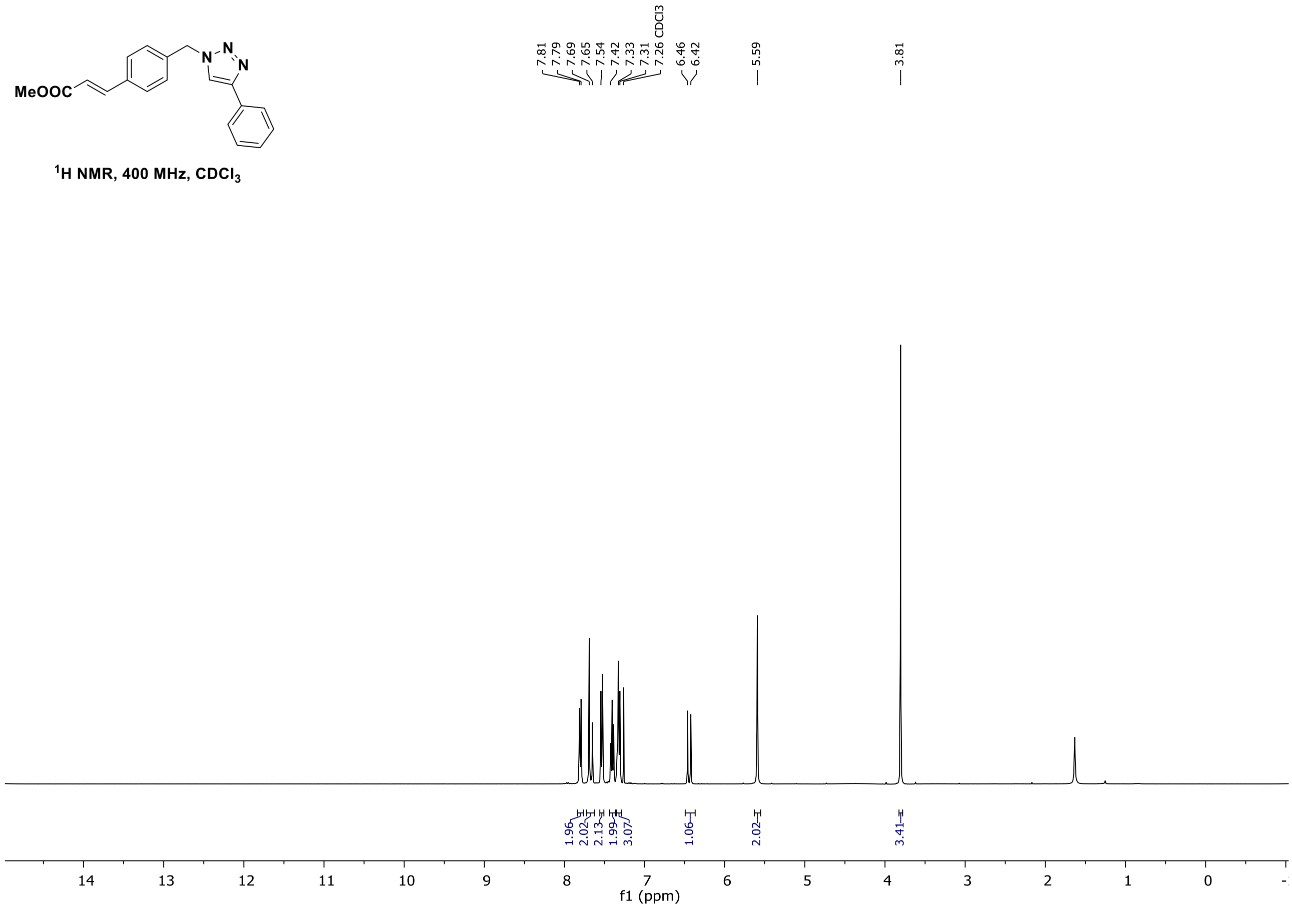

^{1}H NMR, 400 MHz, CDCl$_3$

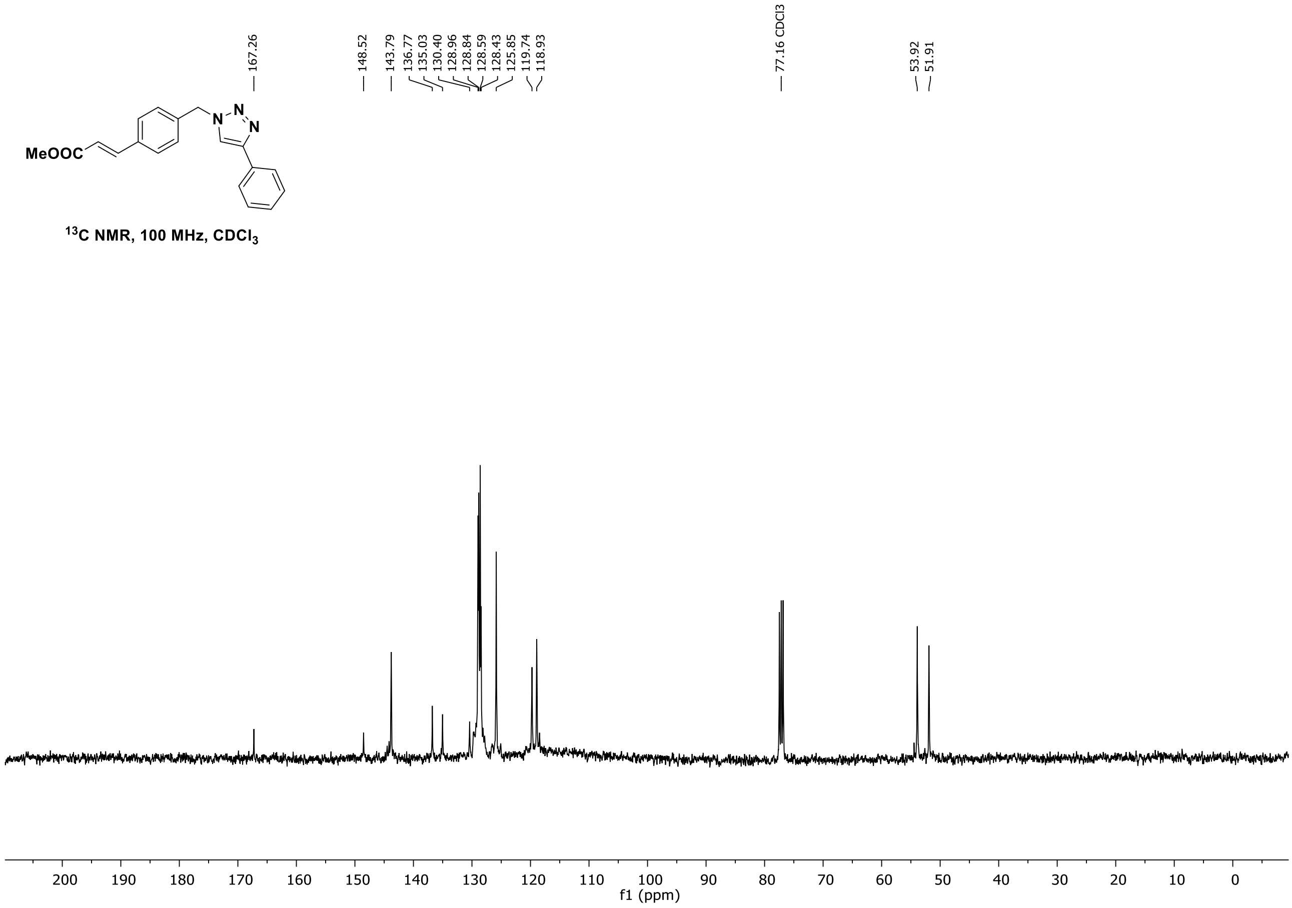

^{13}C NMR, 100 MHz, CDCl$_3$

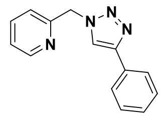

^{1}H NMR, 400 MHz, $CDCl_3$

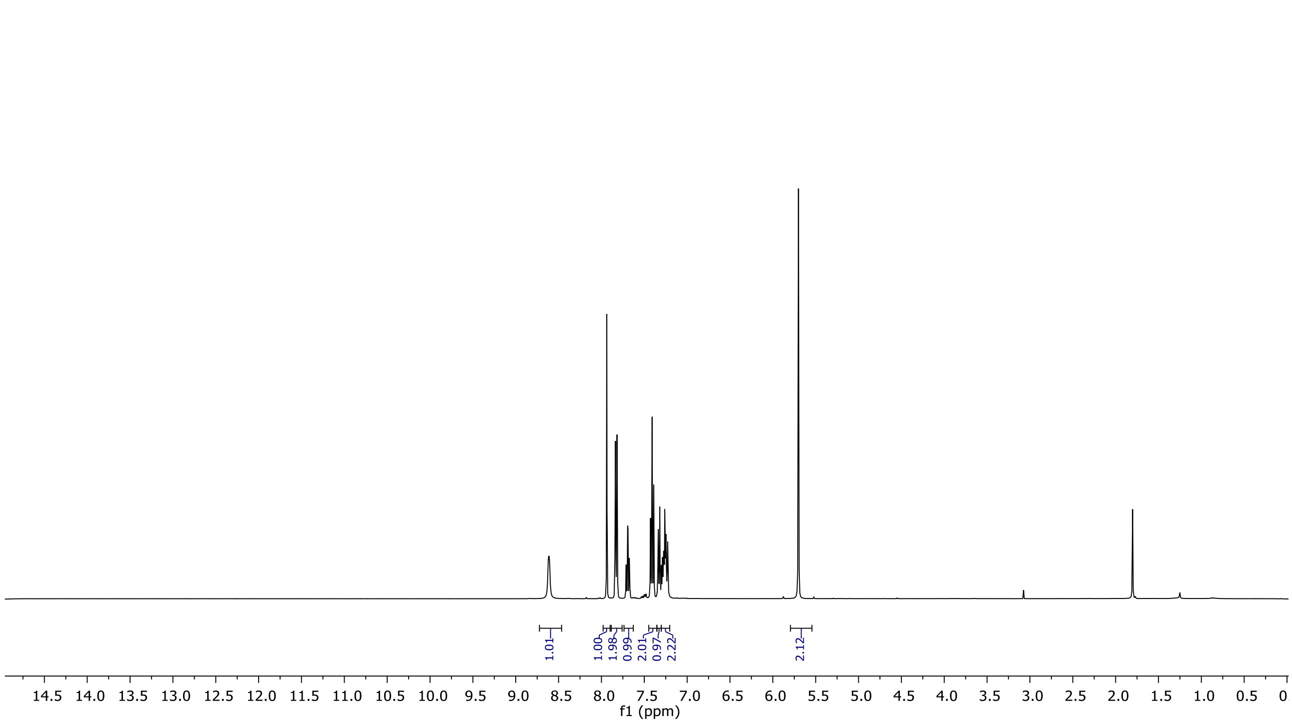

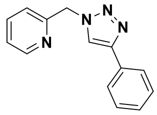

^{13}C NMR, 100 MHz, $CDCl_3$

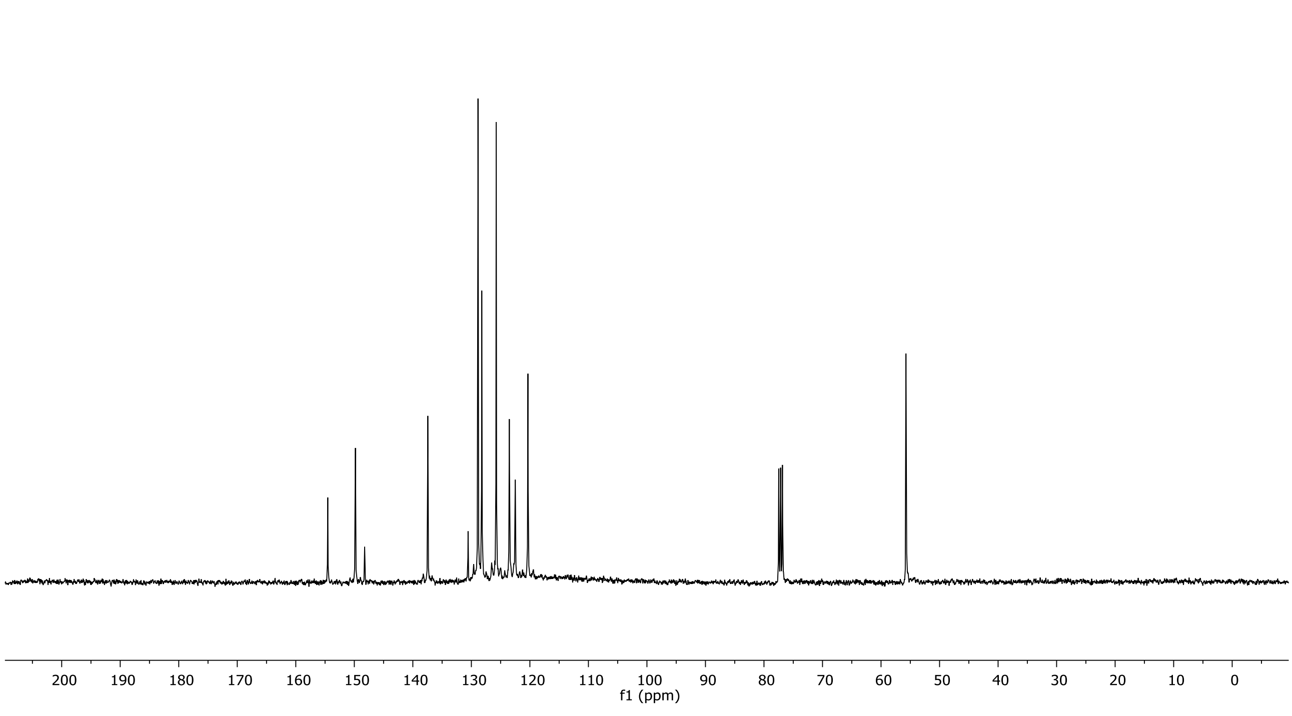

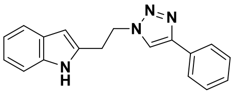

^{1}H NMR, 400 MHz, $CDCl_3$

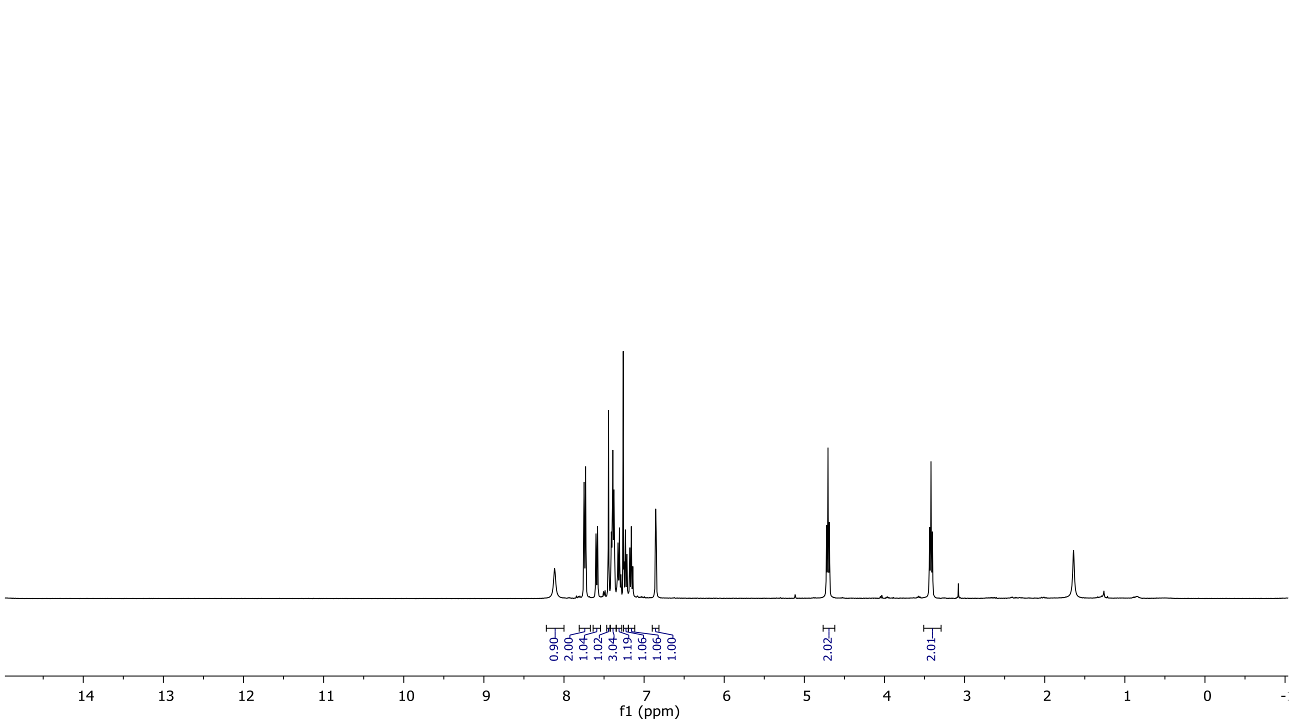

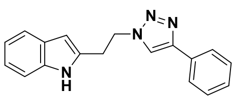

^{13}C NMR, 100 MHz, $CDCl_3$

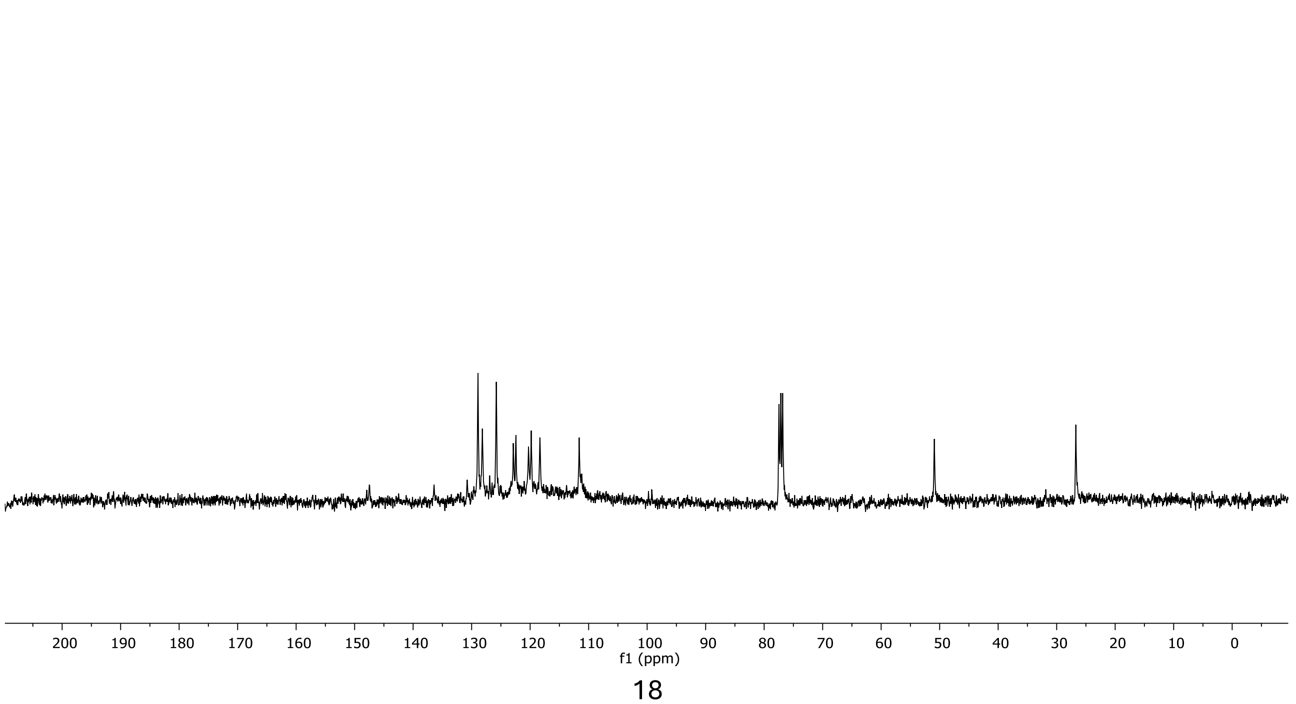

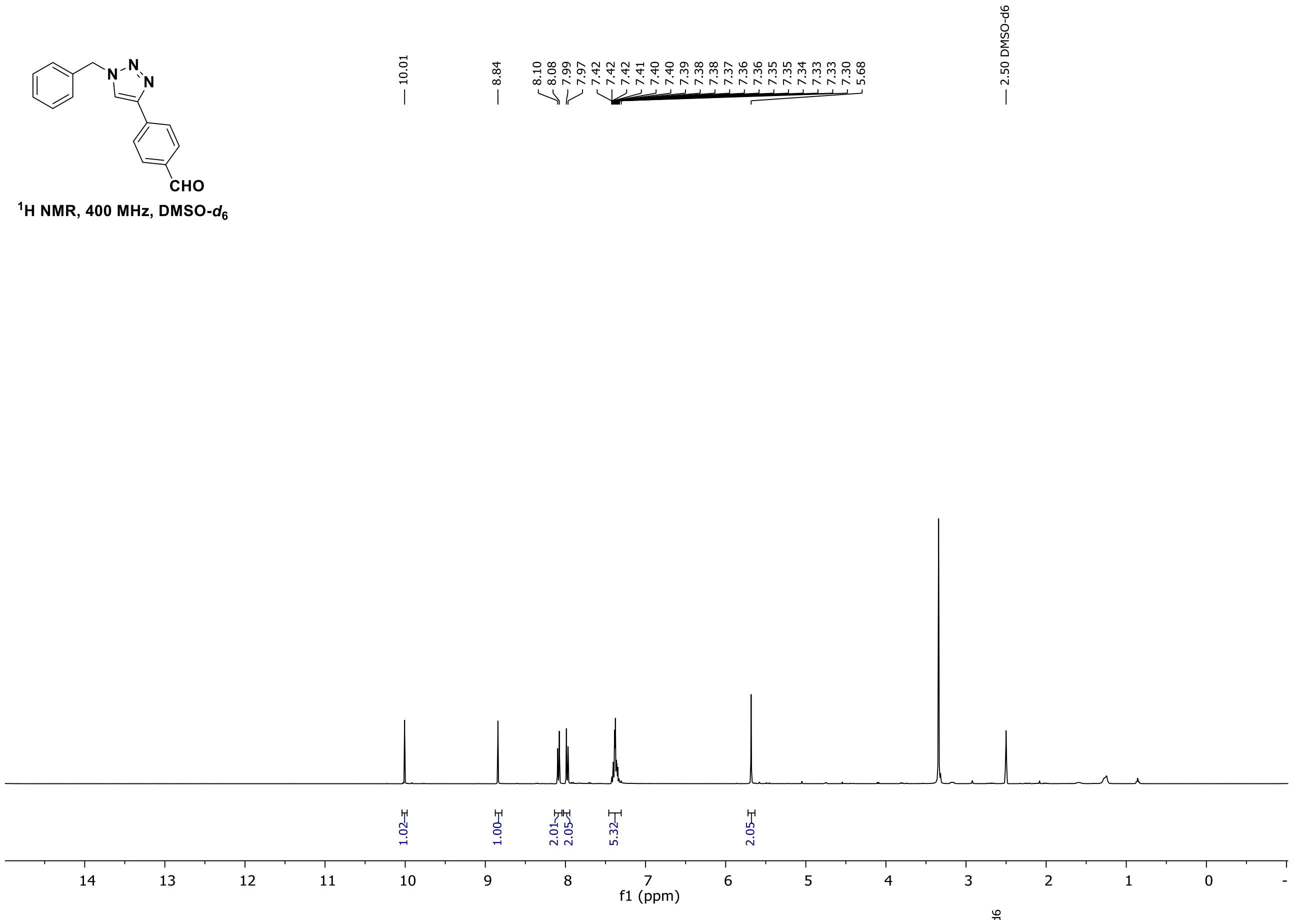

^{1}H NMR, 400 MHz, DMSO-d_6

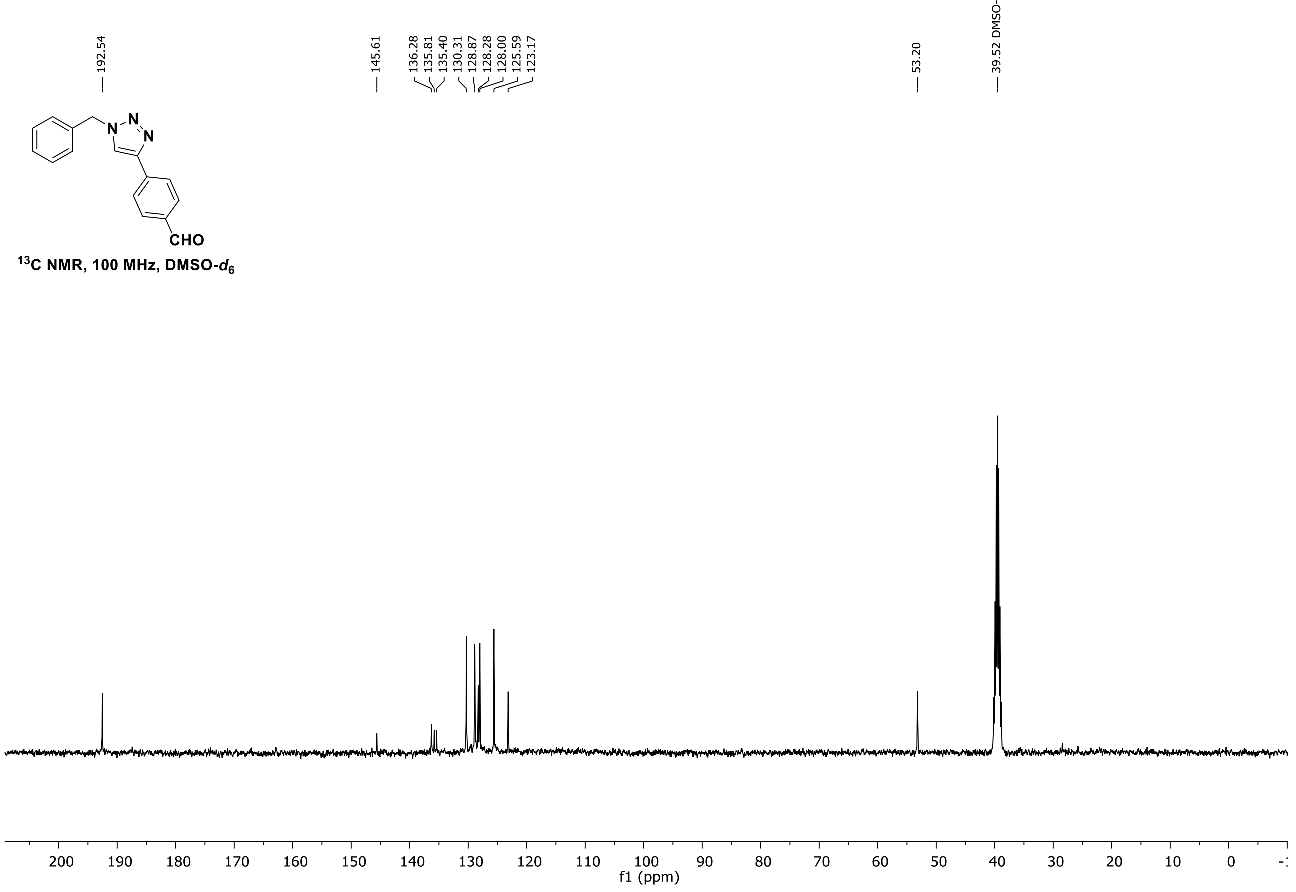

^{13}C NMR, 100 MHz, DMSO-d_6

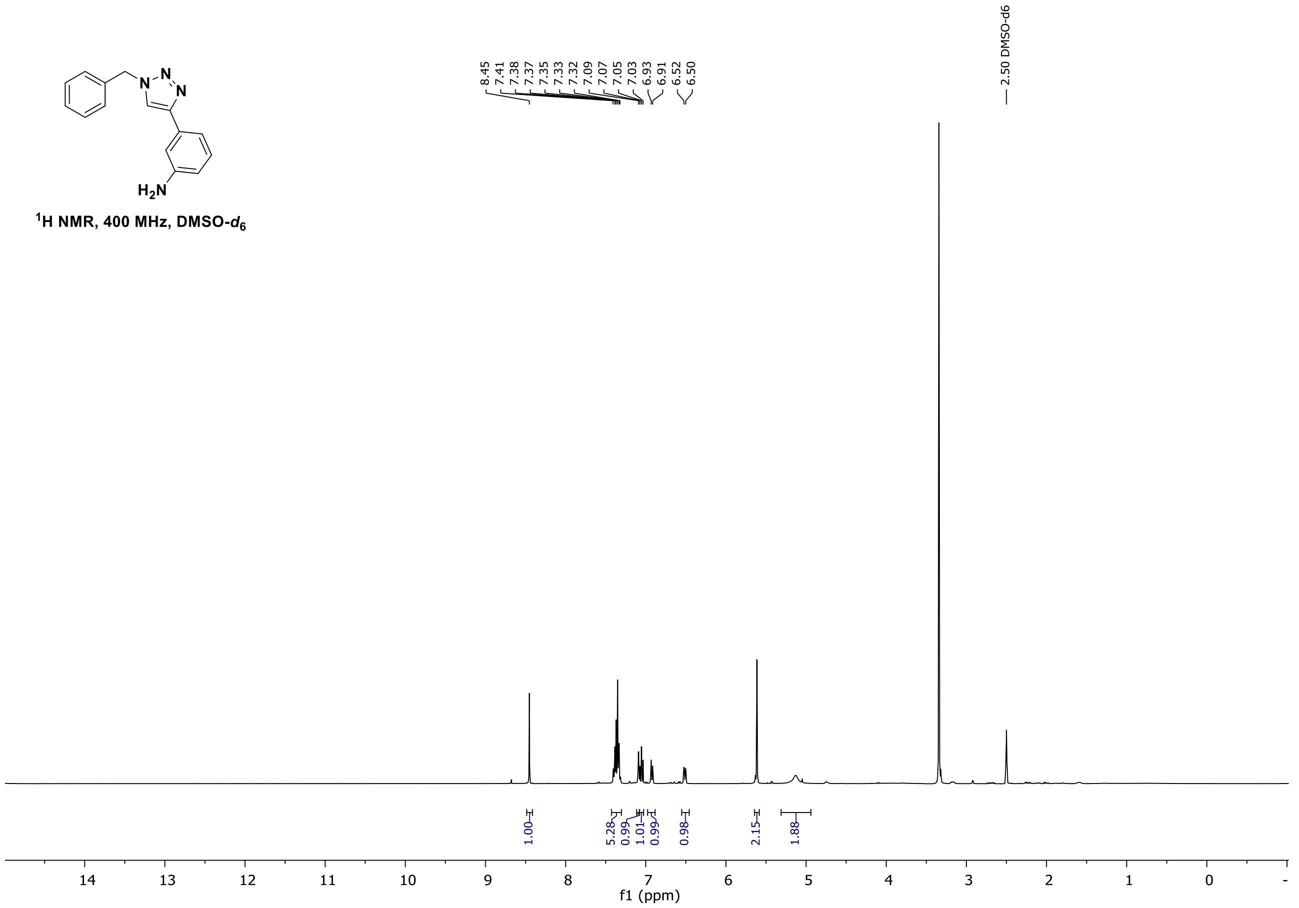

H2N
N
N
N
1H NMR, 400 MHz, DMSO-d6
8.45
7.41
7.38
7.37
7.35
7.33
7.32
7.09
7.07
7.05
7.03
6.93
6.91
6.52
6.50
2.50 DMSO-d6
1.00
5.28
0.99
1.01
0.99
0.98
2.15
1.88
14
13
12
11
10
9
8
7
6
5
4
3
2
1
0
f1 (ppm)

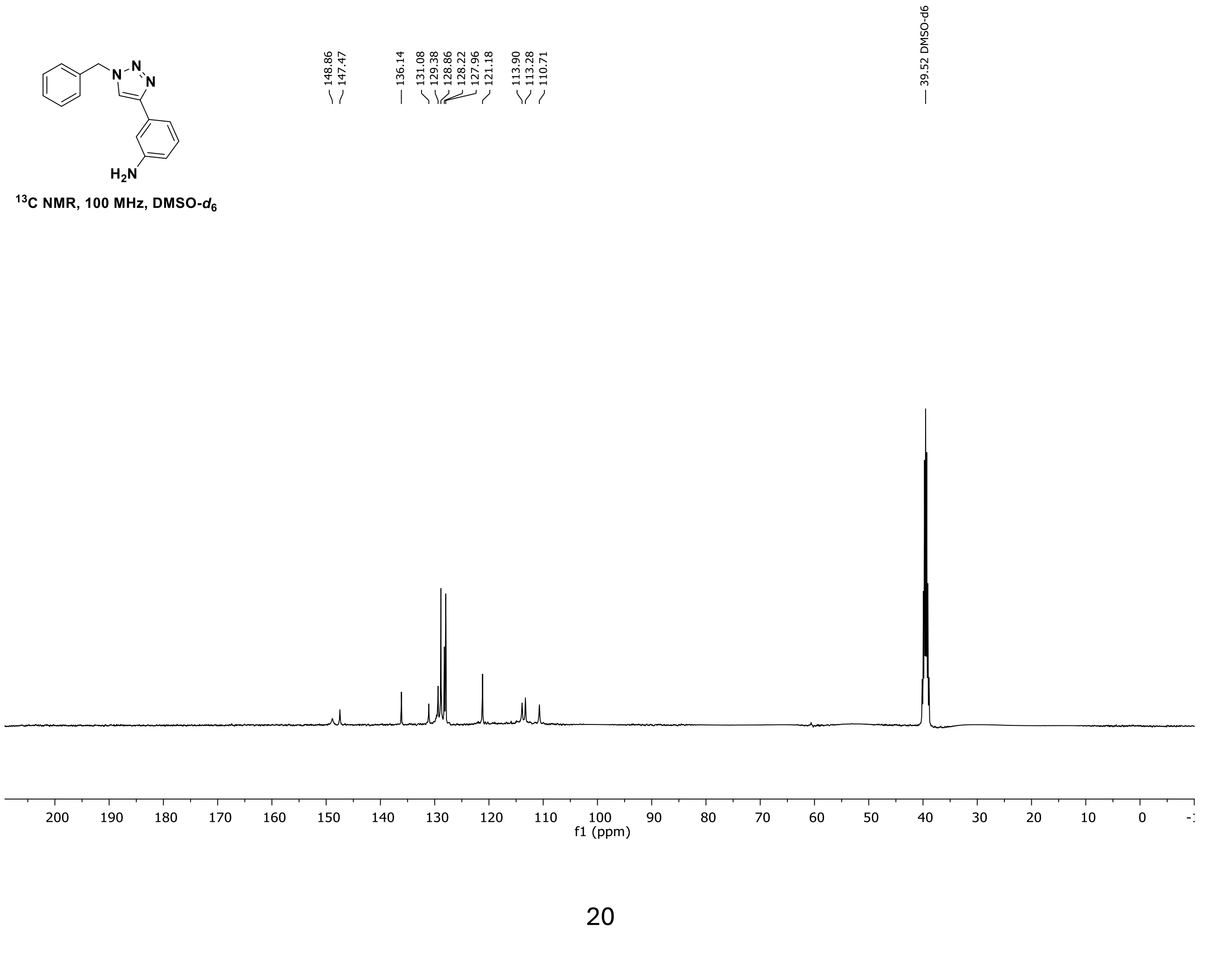

H2N
N
N
N
13C NMR, 100 MHz, DMSO-d6
148.86
147.47
136.14
131.08
129.38
128.86
128.22
127.96
121.18
113.90
113.28
110.71
39.52 DMSO-d6
200
190
180
170
160
150
140
130
120
110
100
90
80
70
60
50
40
30
20
10
0
f1 (ppm)

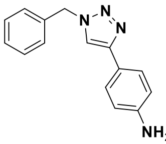

^{1}H NMR, 400 MHz, DMSO-d_6

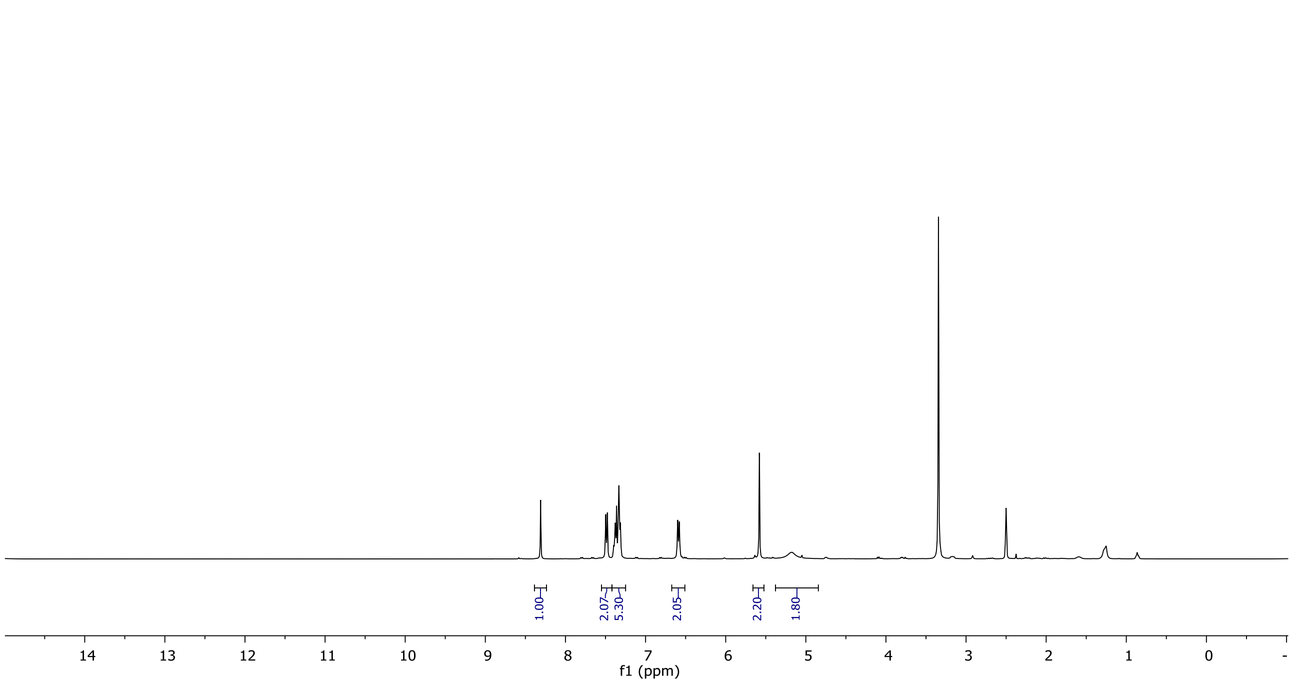

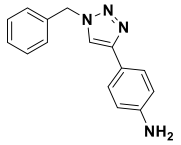

^{13}C NMR, 100 MHz, DMSO-d_6

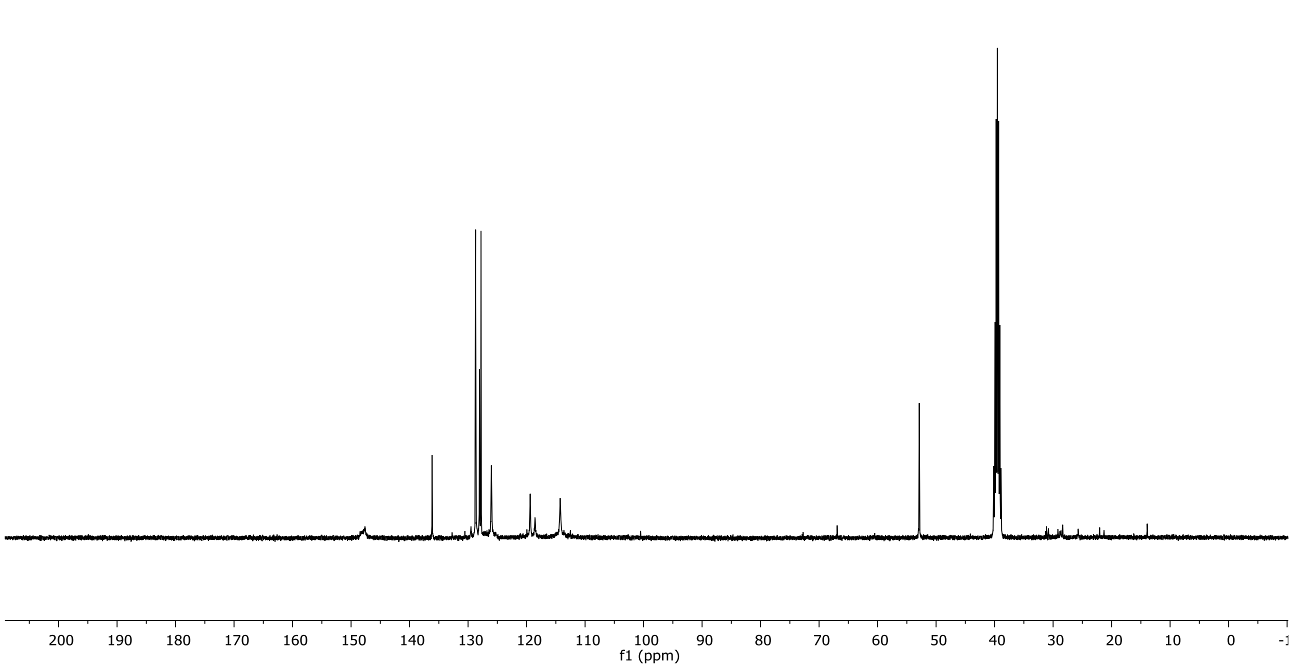

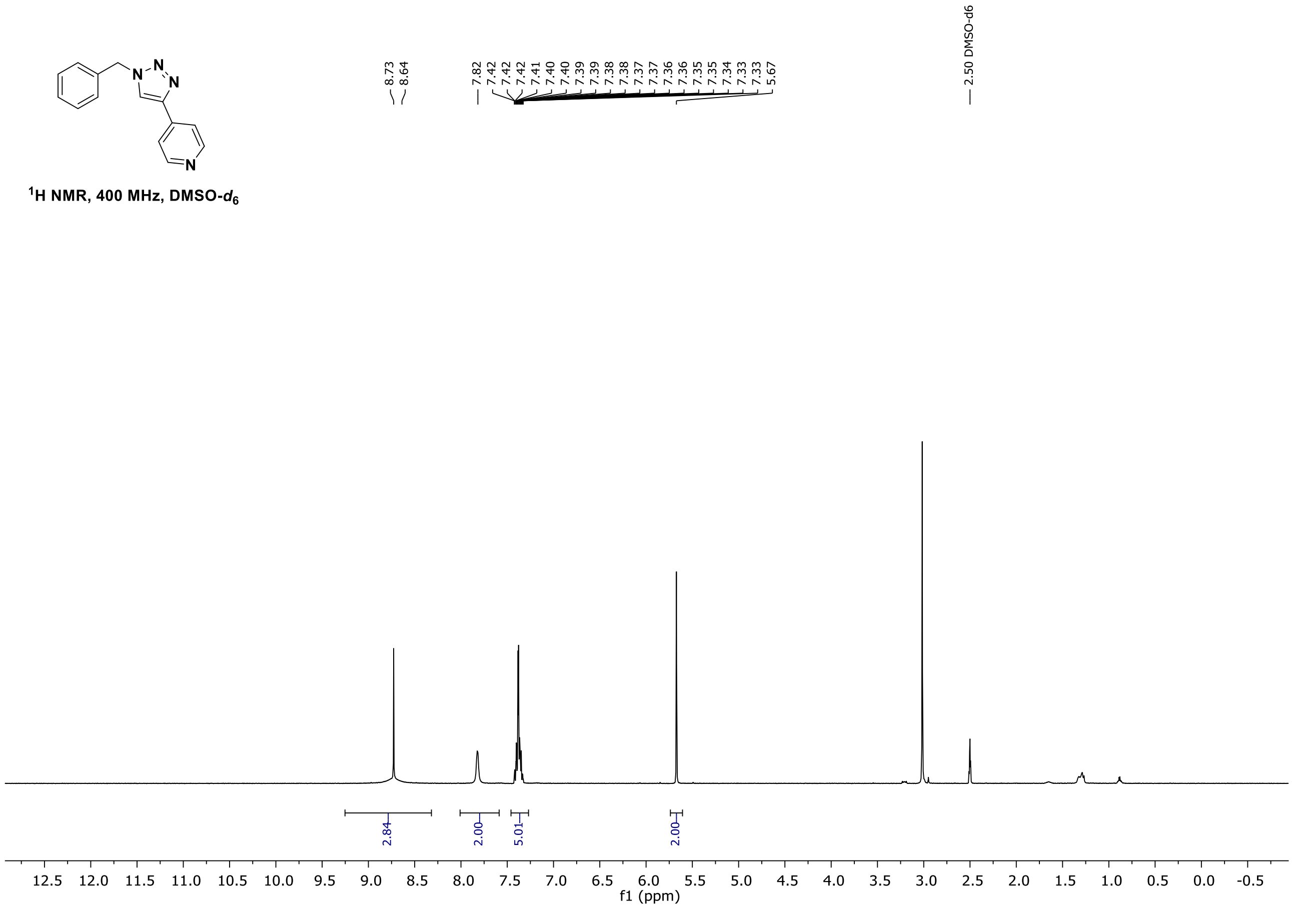

^{1}H NMR, 400 MHz, DMSO-d_6

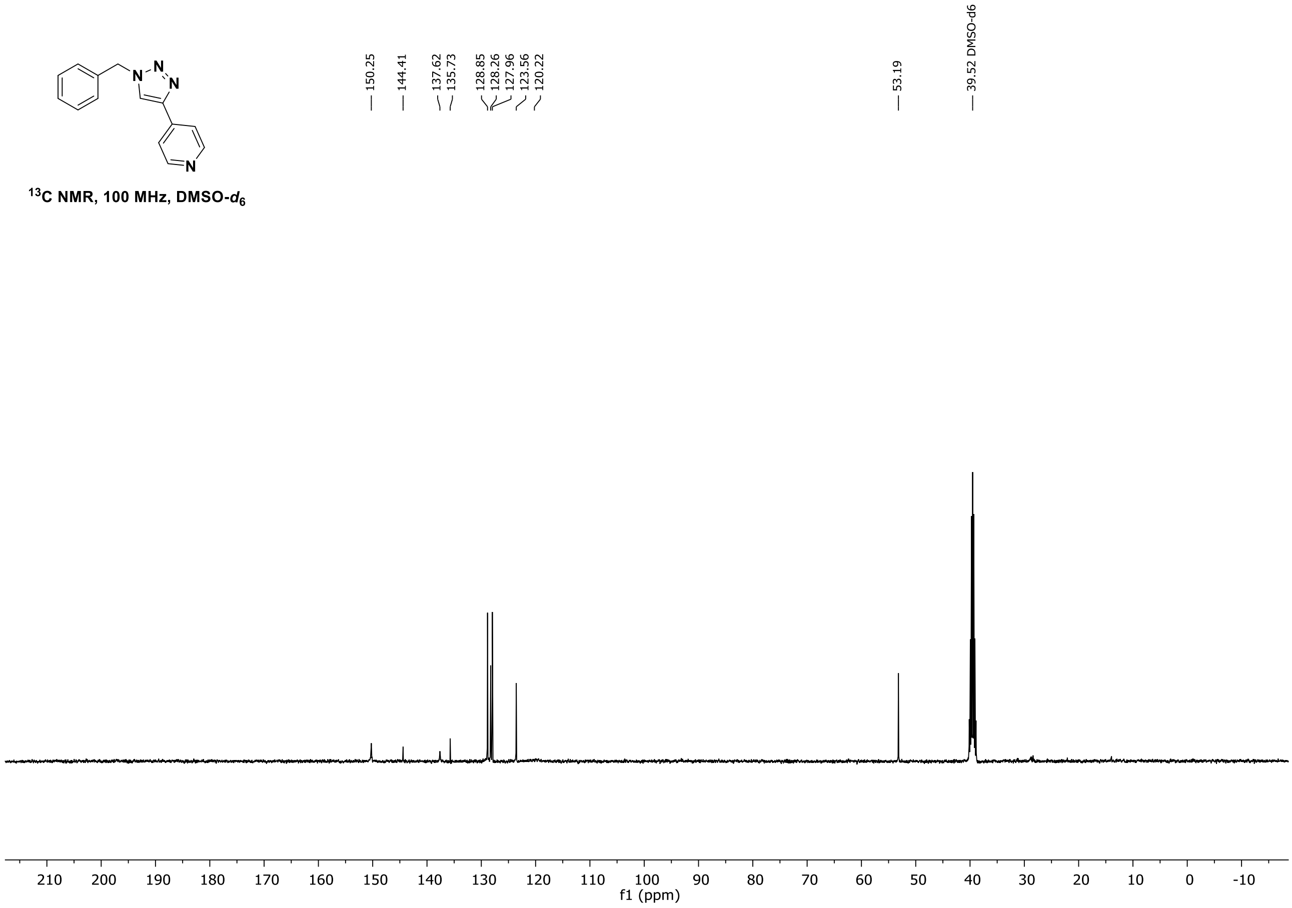

^{13}C NMR, 100 MHz, DMSO-d_6

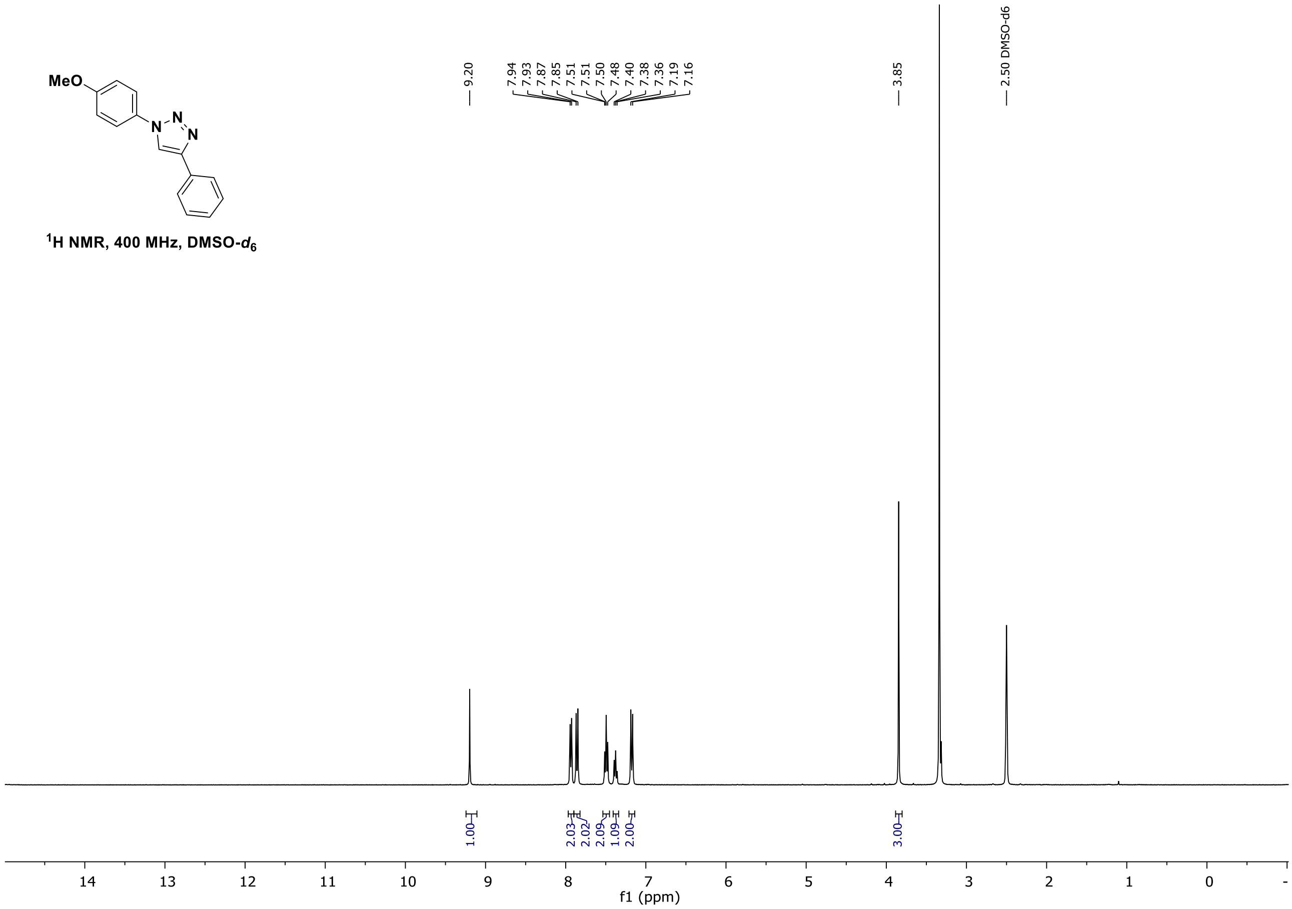
MeO
N
N
N
1H NMR, 400 MHz, DMSO-d6
9.20
7.94
7.93
7.87
7.85
7.51
7.51
7.50
7.48
7.40
7.38
7.36
7.19
7.16
3.85
2.50 DMSO-d6
1.00
2.03
2.02
2.09
1.09
2.00
3.00
f1 (ppm)

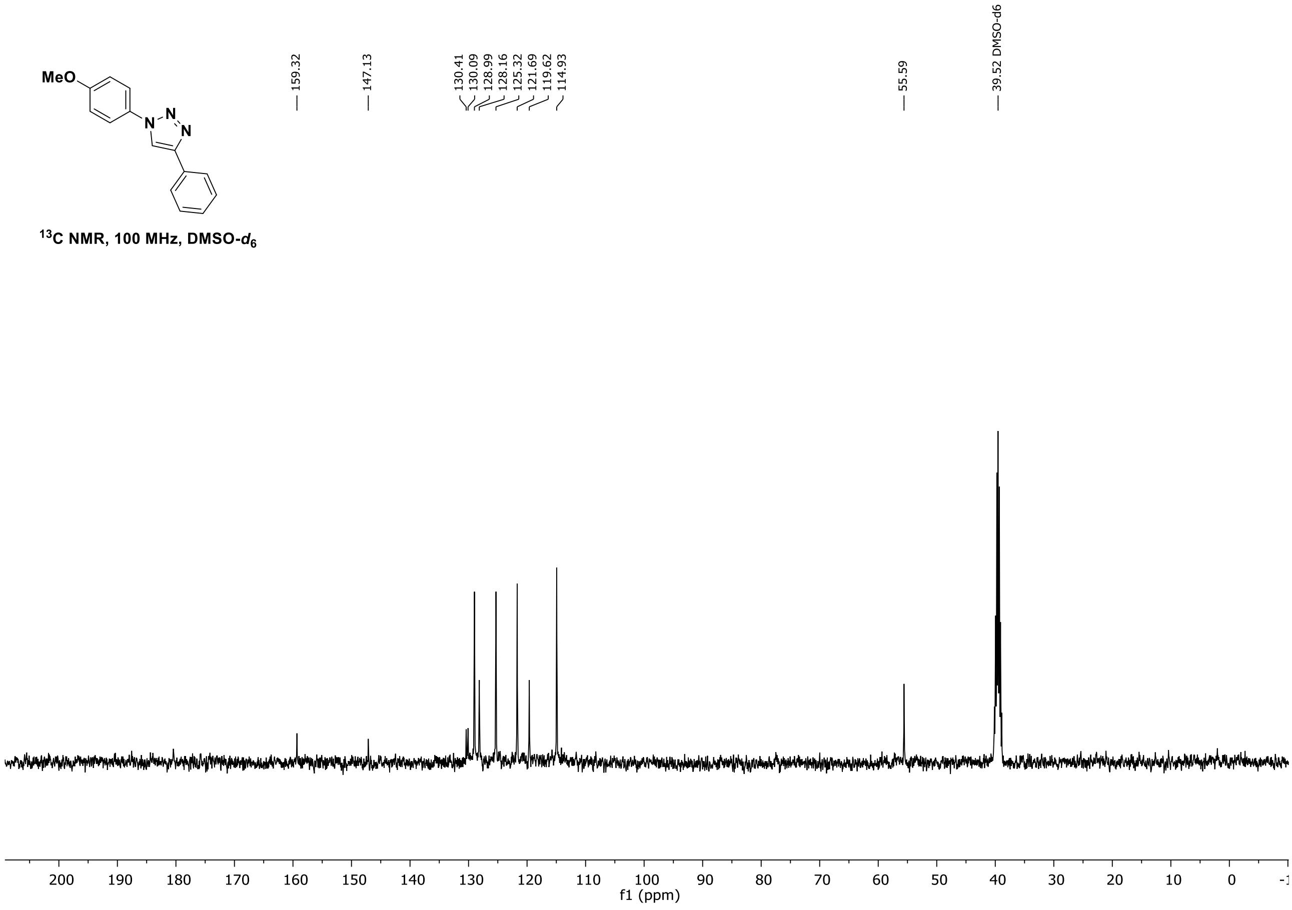
MeO
N
N
N
13C NMR, 100 MHz, DMSO-d6
159.32
147.13
130.41
130.09
128.99
128.16
125.32
121.69
119.62
114.93
55.59
39.52 DMSO-d6
f1 (ppm)

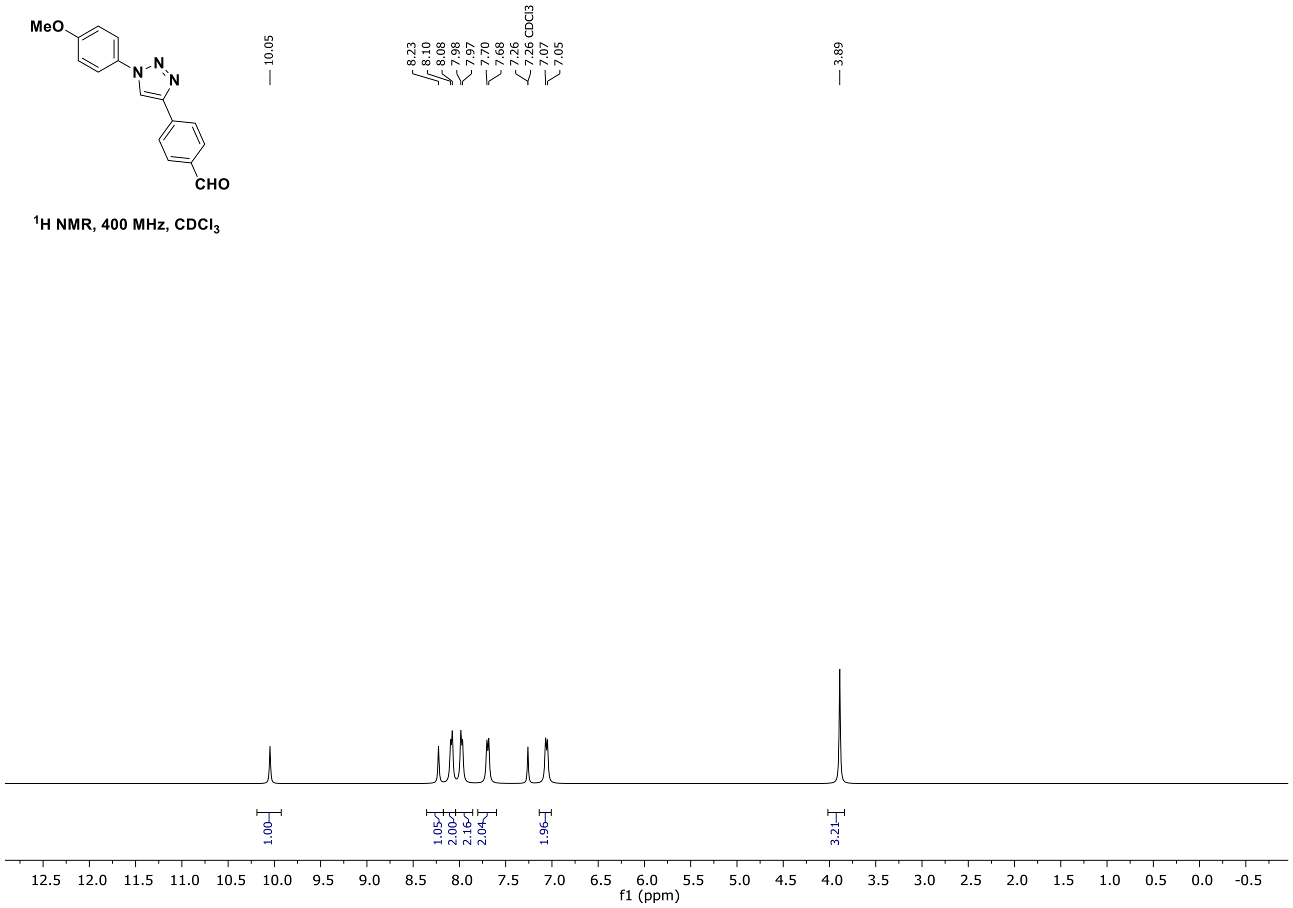
MeO
N
N
N
CHO
1H NMR, 400 MHz, CDCl3
10.05
8.23
8.10
8.08
7.98
7.97
7.70
7.68
7.26 CDCl3
7.07
7.05
3.89
1.00
1.05
2.00
2.16
2.04
1.96
3.21
f1 (ppm)

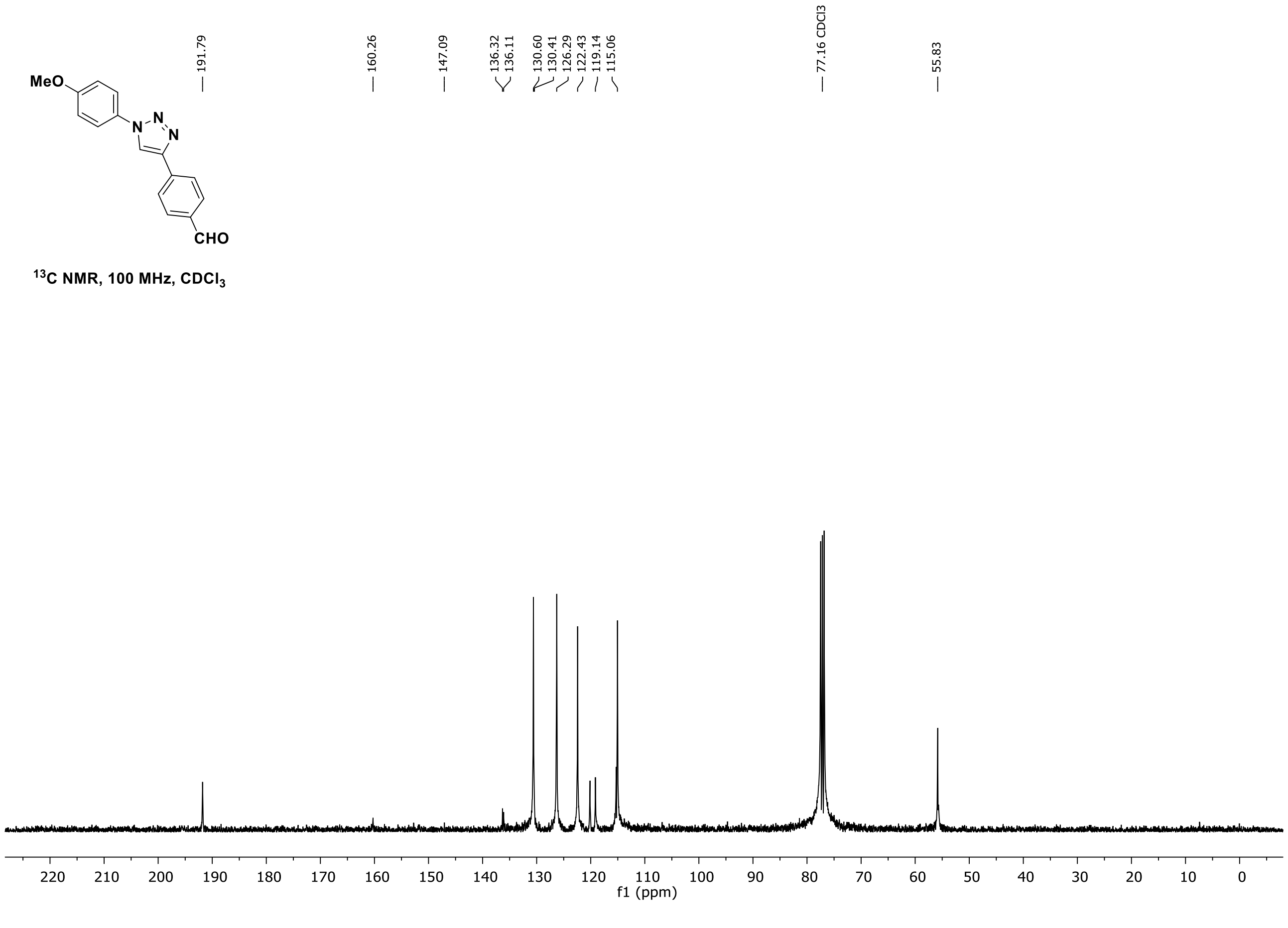
MeO
N
N
N
CHO
13C NMR, 100 MHz, CDCl3
191.79
160.26
147.09
136.32
136.11
130.60
130.41
126.29
122.43
119.14
115.06
77.16 CDCl3
55.83
f1 (ppm)

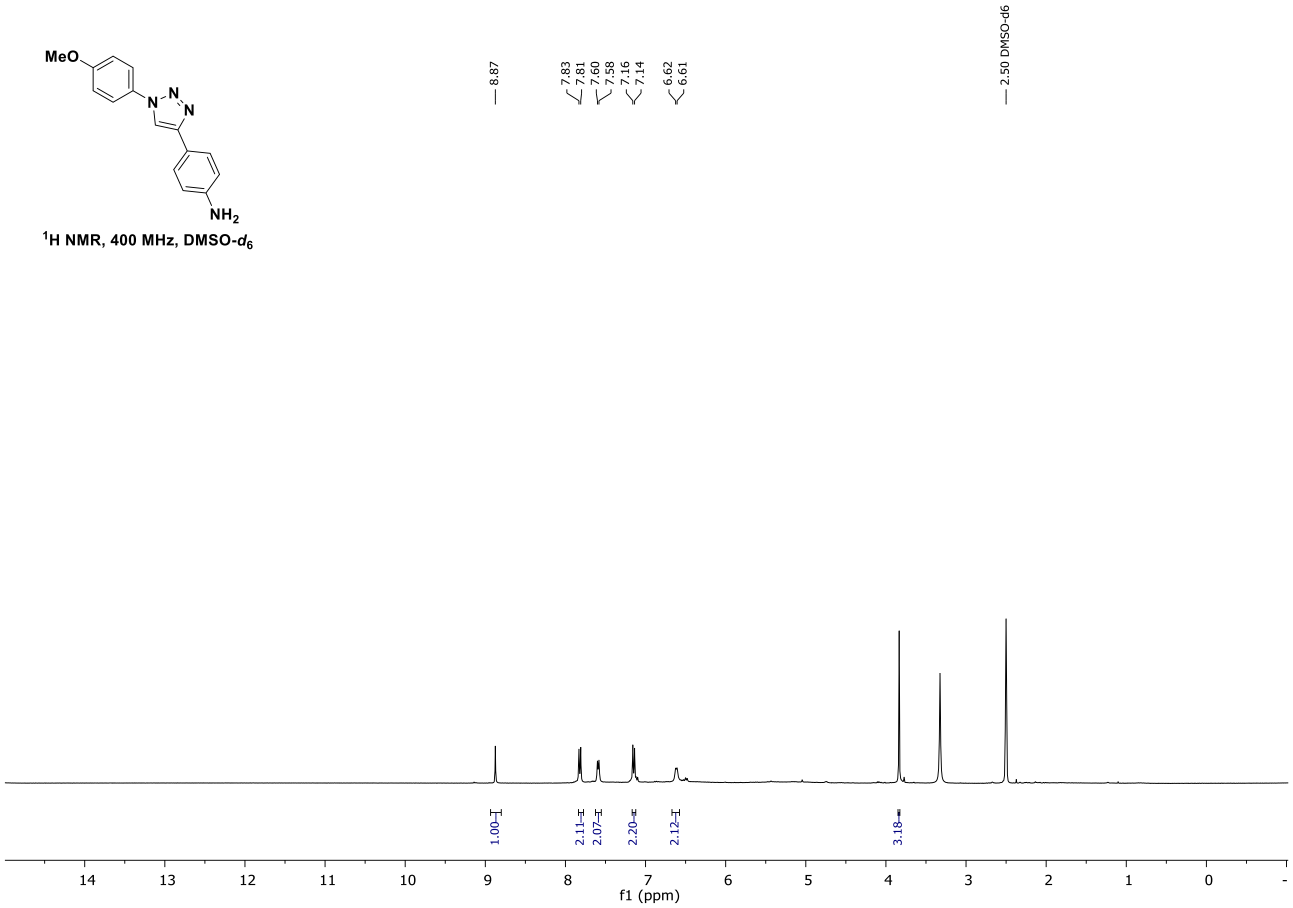
MeO
N
N
N
NH2
1H NMR, 400 MHz, DMSO-d6
8.87
7.83
7.81
7.60
7.58
7.16
7.14
6.62
6.61
2.50 DMSO-d6
1.00
2.11
2.07
2.20
2.12
3.18
14 13 12 11 10 9 8 7 6 5 4 3 2 1 0
f1 (ppm)

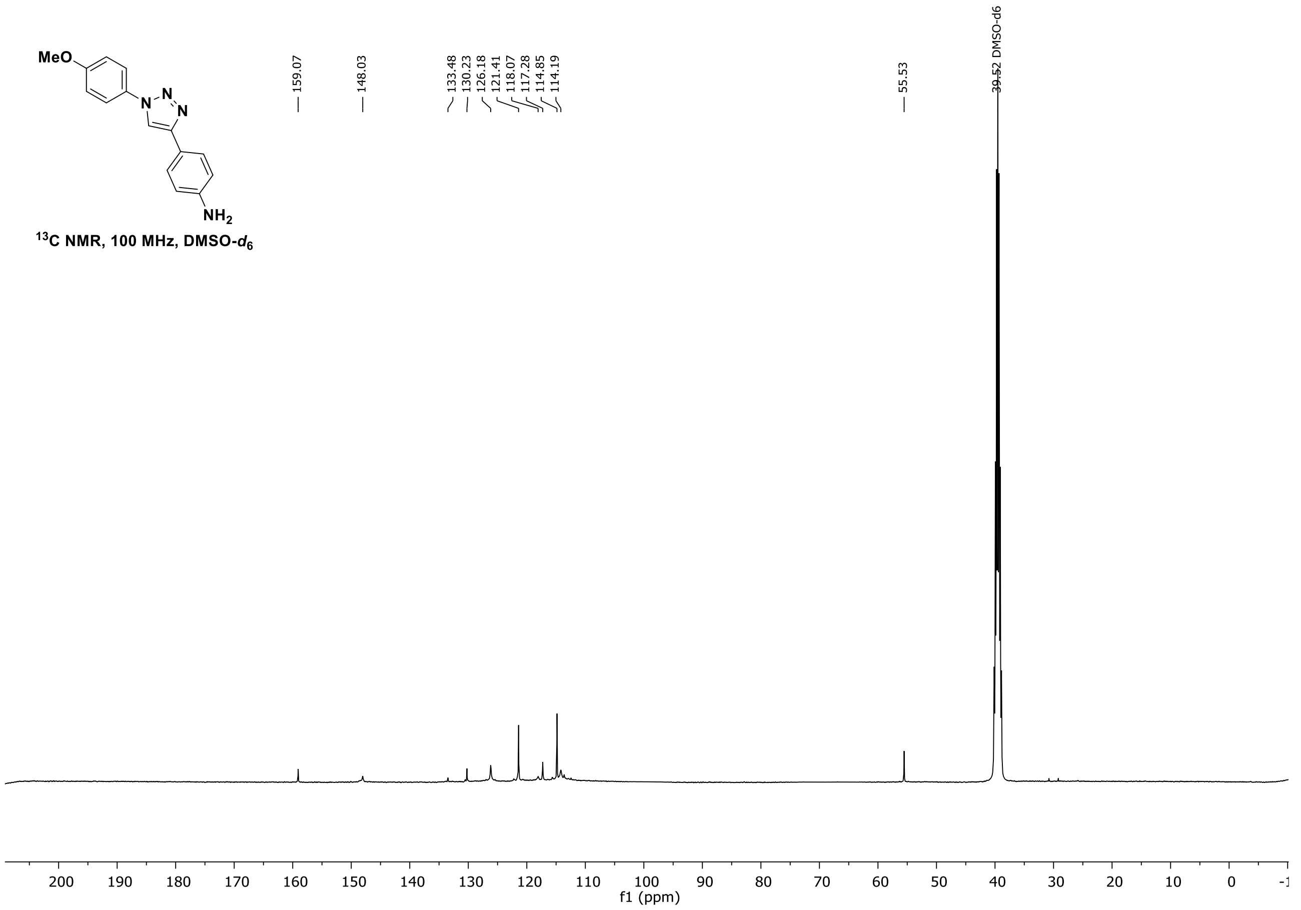
MeO
N
N
N
NH2
13C NMR, 100 MHz, DMSO-d6
159.07
148.03
133.48
130.23
126.18
121.41
118.07
117.28
114.85
114.19
55.53
39.52 DMSO-d6
200 190 180 170 160 150 140 130 120 110 100 90 80 70 60 50 40 30 20 10 0
f1 (ppm)

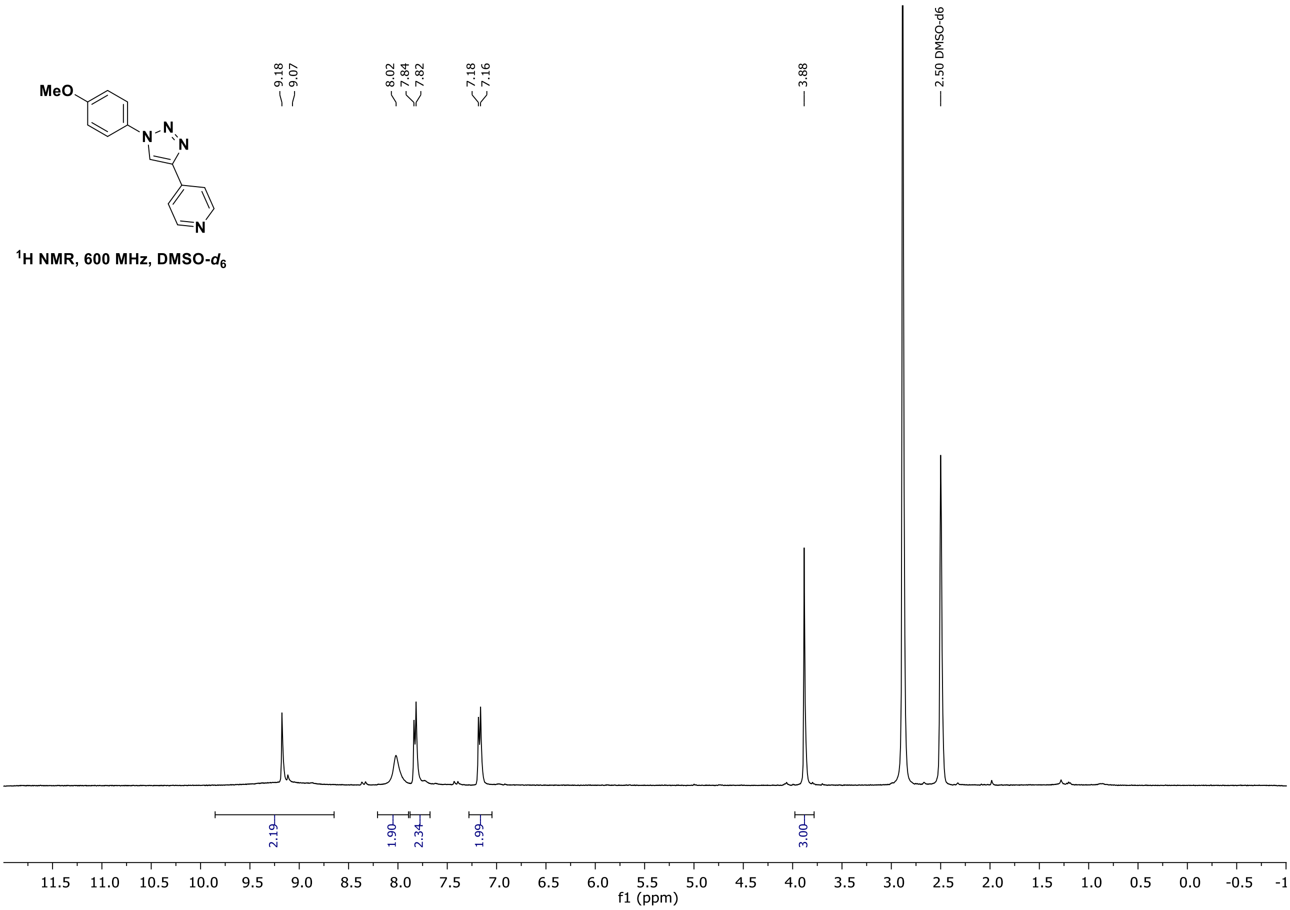

MeO
N
N
N
N
¹H NMR, 600 MHz, DMSO-d6
9.18
9.07
8.02
7.84
7.82
7.18
7.16
3.88
2.50 DMSO-d6
2.19
1.90
2.34
1.99
3.00
11.5 11.0 10.5 10.0 9.5 9.0 8.5 8.0 7.5 7.0 6.5 6.0 5.5 5.0 4.5 4.0 3.5 3.0 2.5 2.0 1.5 1.0 0.5 0.0 -0.5 -1
f1 (ppm)

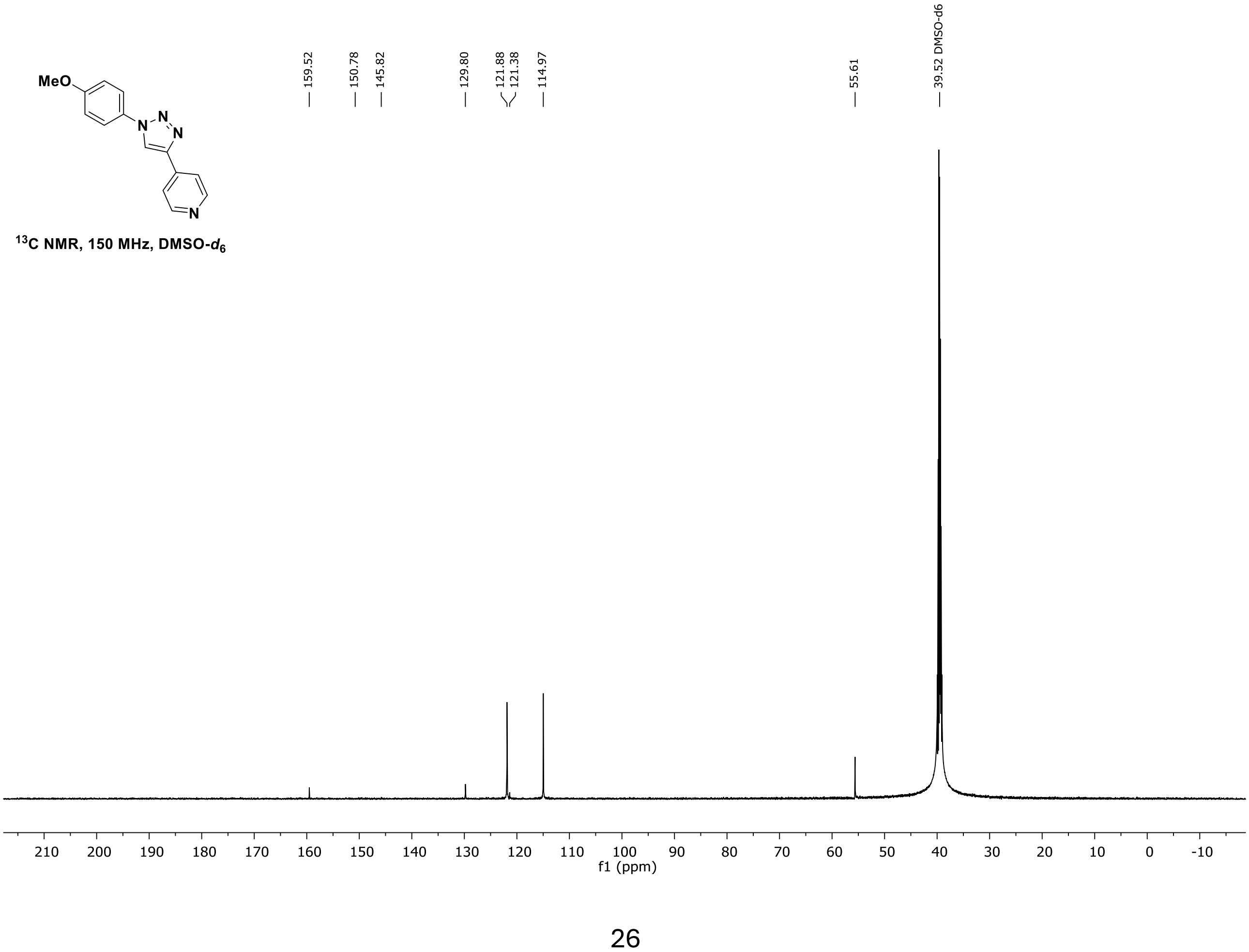

MeO
N
N
N
N
¹³C NMR, 150 MHz, DMSO-d6
159.52
150.78
145.82
129.80
121.88
121.38
114.97
55.61
39.52 DMSO-d6
210 200 190 180 170 160 150 140 130 120 110 100 90 80 70 60 50 40 30 20 10 0 -10
f1 (ppm)

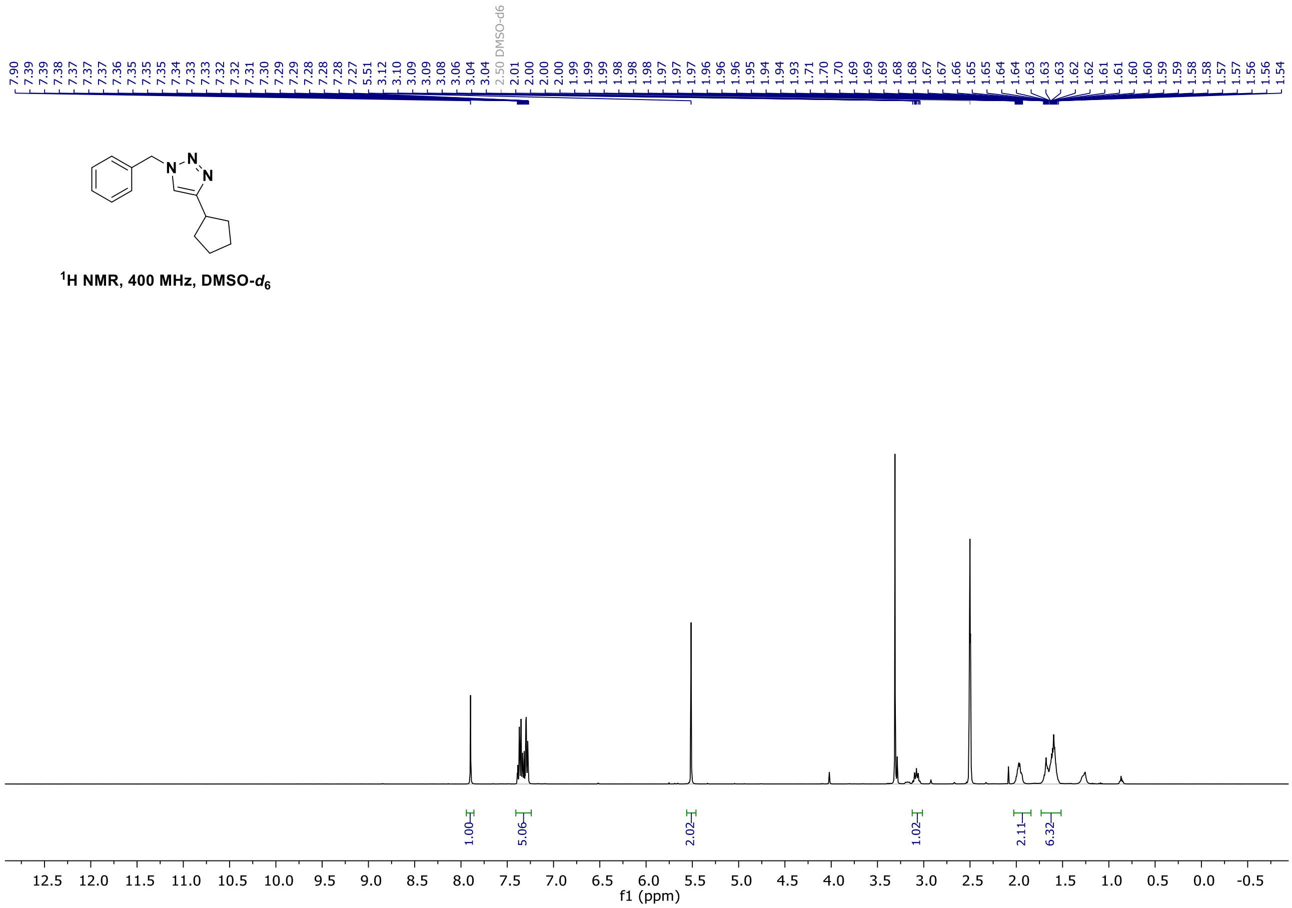

^{1}H NMR, 400 MHz, DMSO-d_6

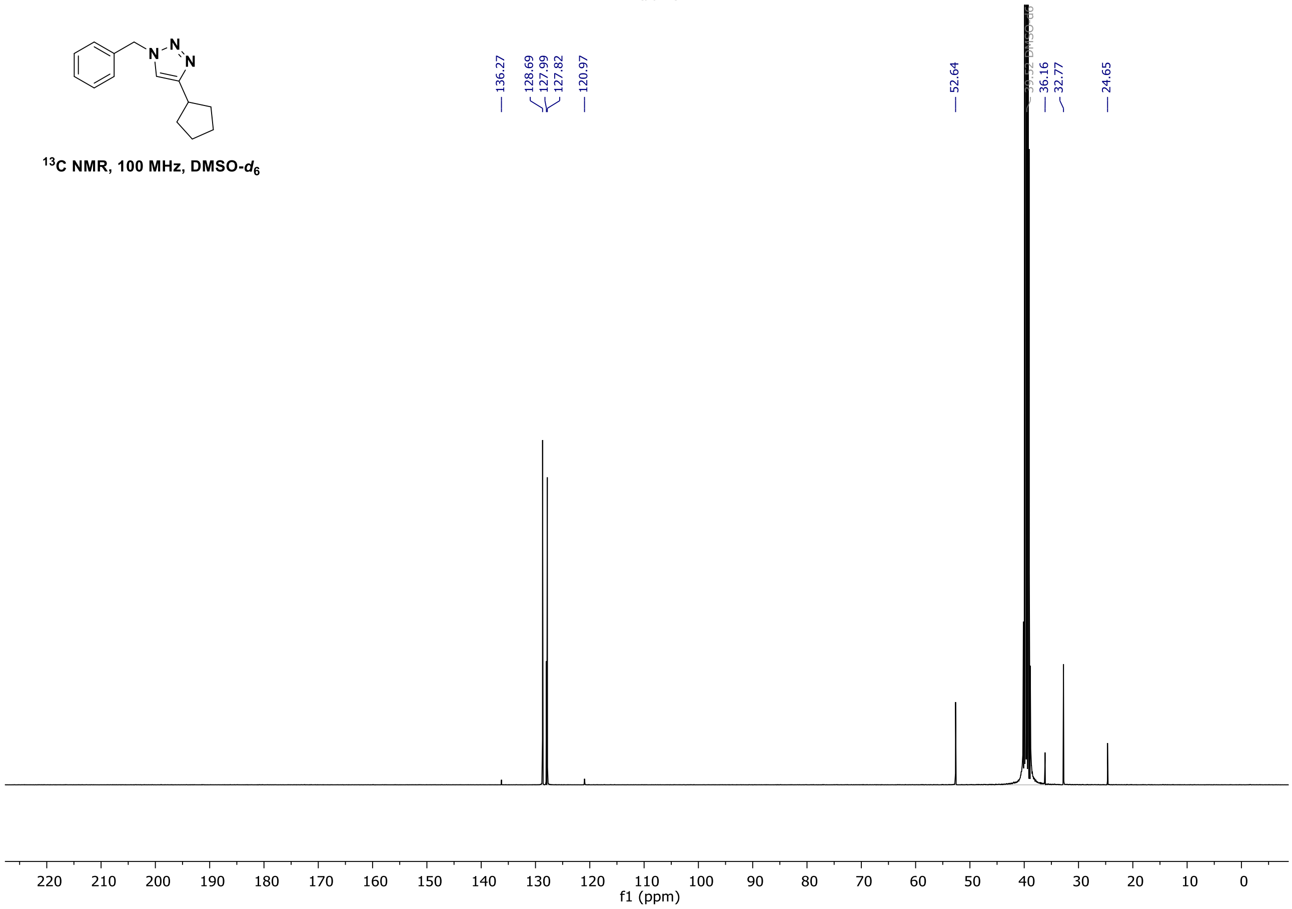

^{13}C NMR, 100 MHz, DMSO-d_6

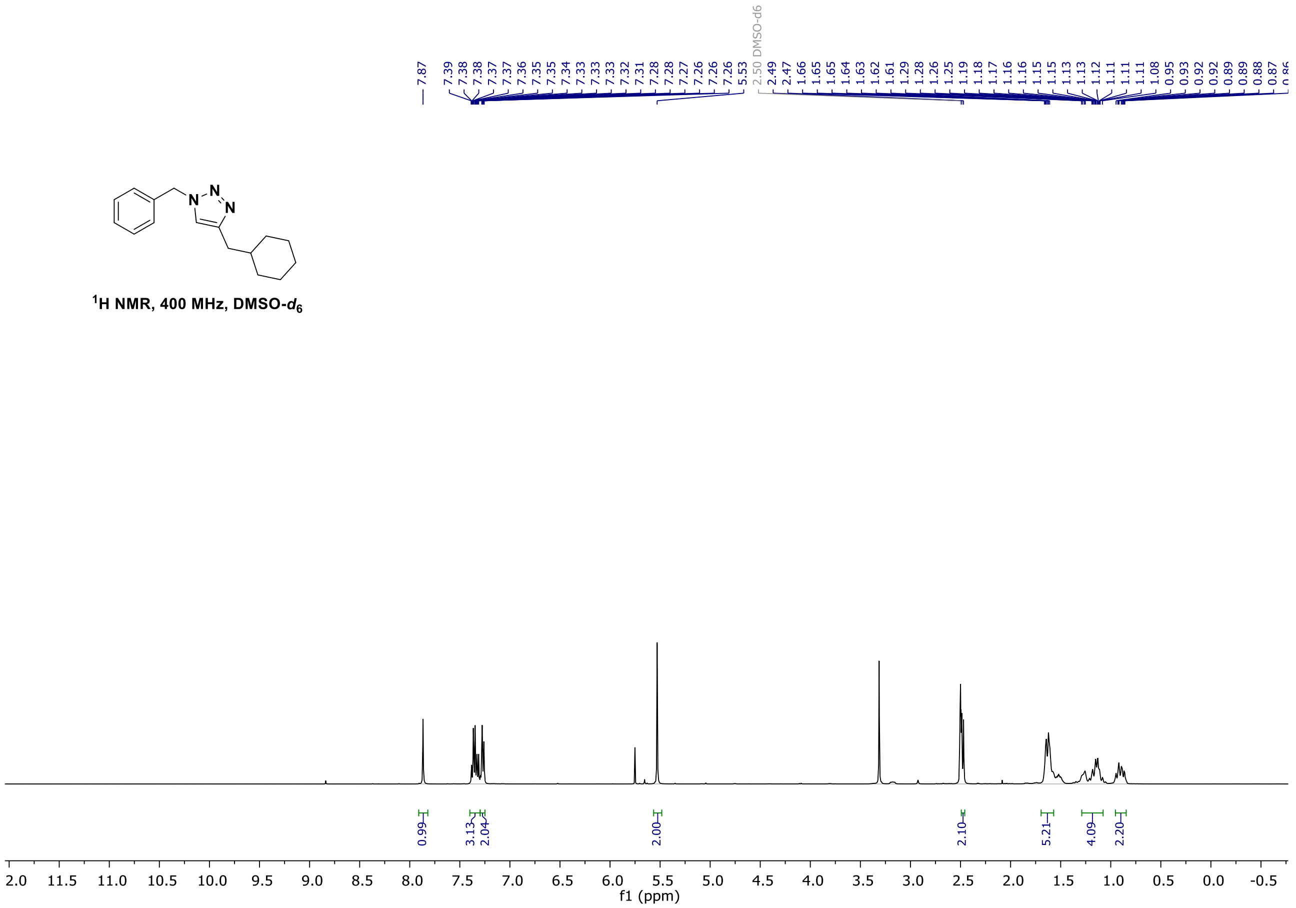

^{1}H NMR, 400 MHz, DMSO-d_6

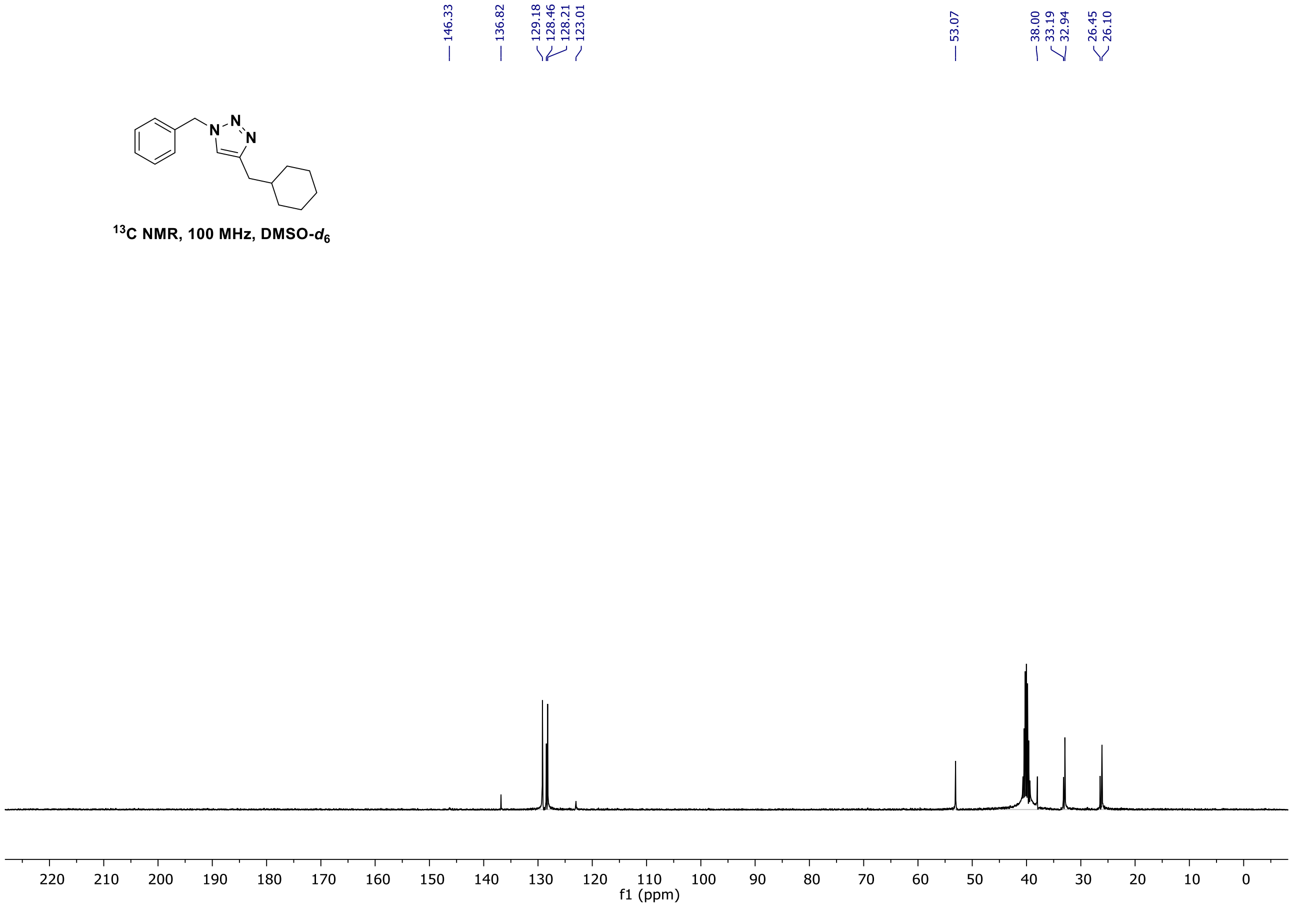

^{13}C NMR, 100 MHz, DMSO-d_6

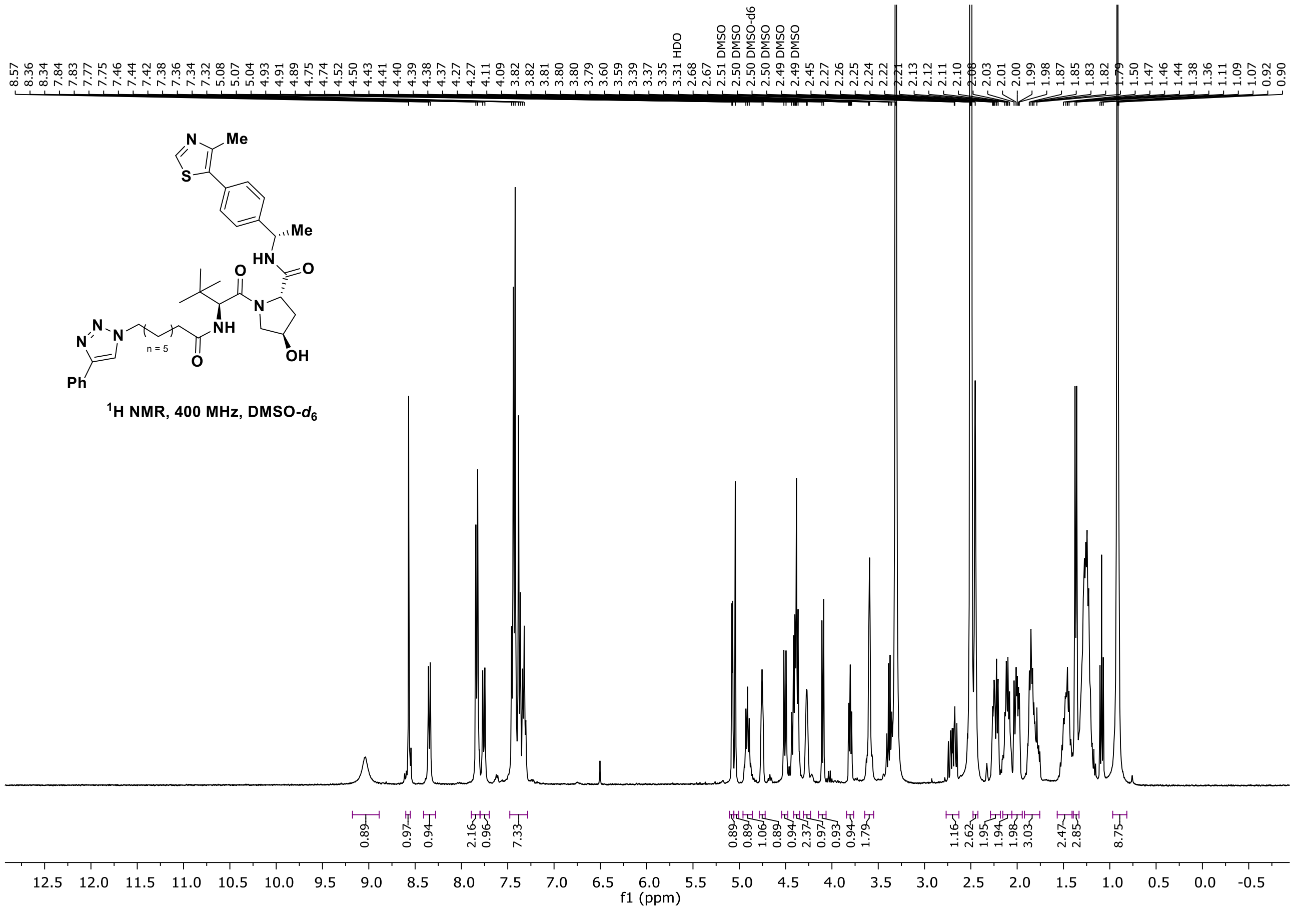
8.57 8.36 8.34 7.84 7.83 7.77 7.75 7.46 7.44 7.42 7.38 7.36 7.34 7.32 5.08 5.07 5.04 4.93 4.91 4.89 4.75 4.74 4.52 4.50 4.43 4.41 4.40 4.39 4.38 4.37 4.27 4.27 4.11 4.09 3.82 3.82 3.81 3.80 3.80 3.79 3.60 3.59 3.39 3.37 3.35 3.31 HDO 2.68 2.67 2.51 DMSO 2.50 DMSO 2.50 DMSO-d6 2.50 DMSO 2.49 DMSO 2.49 DMSO 2.45 2.27 2.26 2.25 2.24 2.22 2.21 2.13 2.12 2.11 2.10 2.08 2.03 2.01 2.00 1.99 1.98 1.87 1.85 1.83 1.82 1.79 1.50 1.47 1.46 1.44 1.38 1.36 1.11 1.09 1.07 0.92 0.90
Me
Me
HN
O
N
O
NH
OH
n = 5
O
Ph
¹H NMR, 400 MHz, DMSO-d6
0.89 0.97 0.94 2.16 0.96 7.33 0.89 0.89 1.06 0.89 0.94 2.37 0.97 0.93 0.94 1.79 1.16 2.62 1.95 1.94 1.98 3.03 2.47 2.85 8.75
12.5 12.0 11.5 11.0 10.5 10.0 9.5 9.0 8.5 8.0 7.5 7.0 6.5 6.0 5.5 5.0 4.5 4.0 3.5 3.0 2.5 2.0 1.5 1.0 0.5 0.0 -0.5
f1 (ppm)

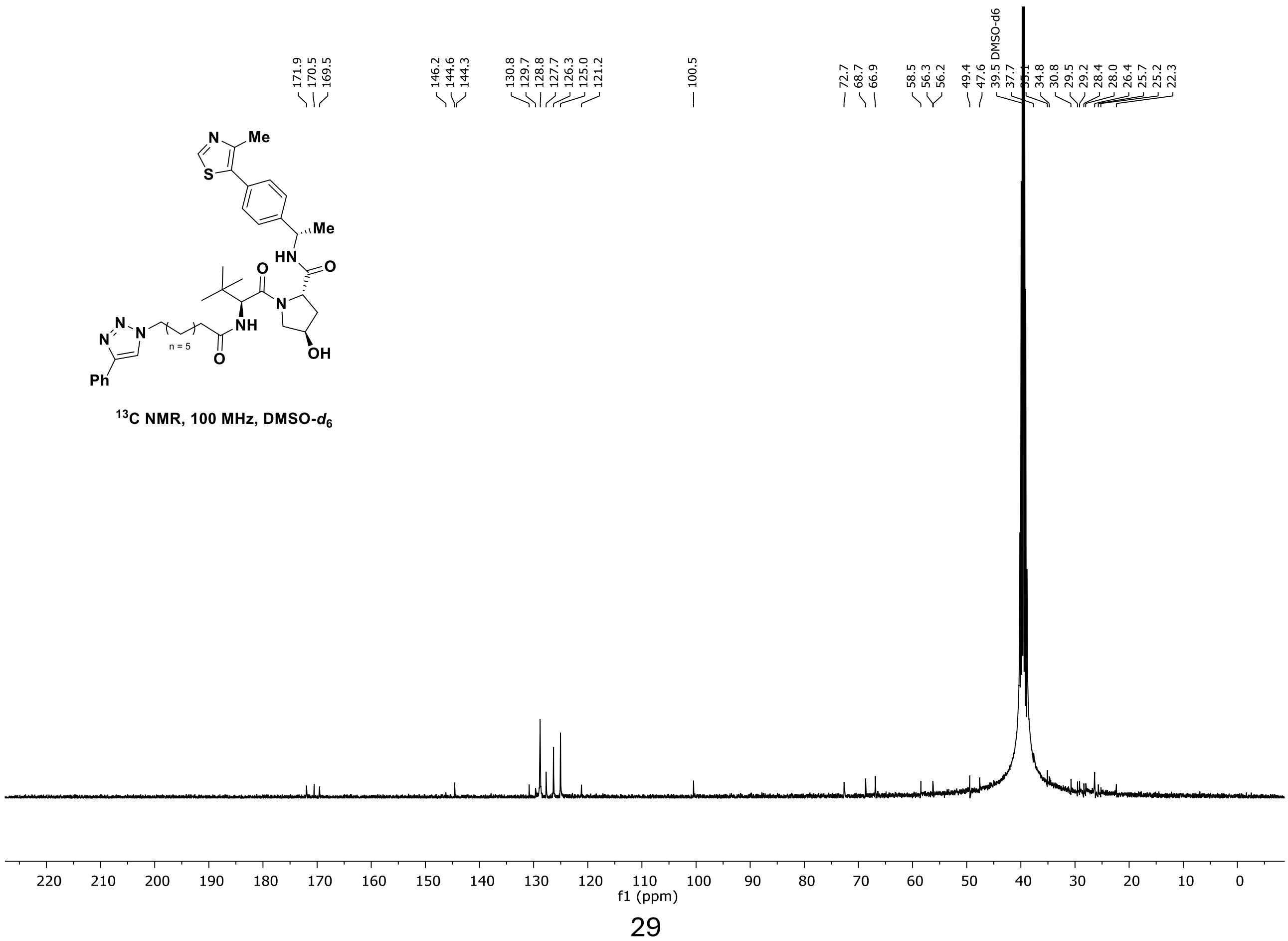
171.9 170.5 169.5 146.2 144.6 144.3 130.8 129.7 128.8 127.7 126.3 125.0 121.2 100.5 72.7 68.7 66.9 58.5 56.3 56.2 49.4 47.6 39.5 DMSO-d6 37.7 35.1 34.8 30.8 29.5 29.2 28.4 28.0 26.4 25.7 25.2 22.3
Me
Me
HN
O
N
O
NH
OH
n = 5
O
Ph
¹³C NMR, 100 MHz, DMSO-d6
220 210 200 190 180 170 160 150 140 130 120 110 100 90 80 70 60 50 40 30 20 10 0
f1 (ppm)

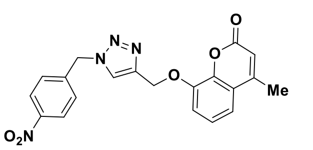

^{1}H NMR, 400 MHz, DMSO-d_6

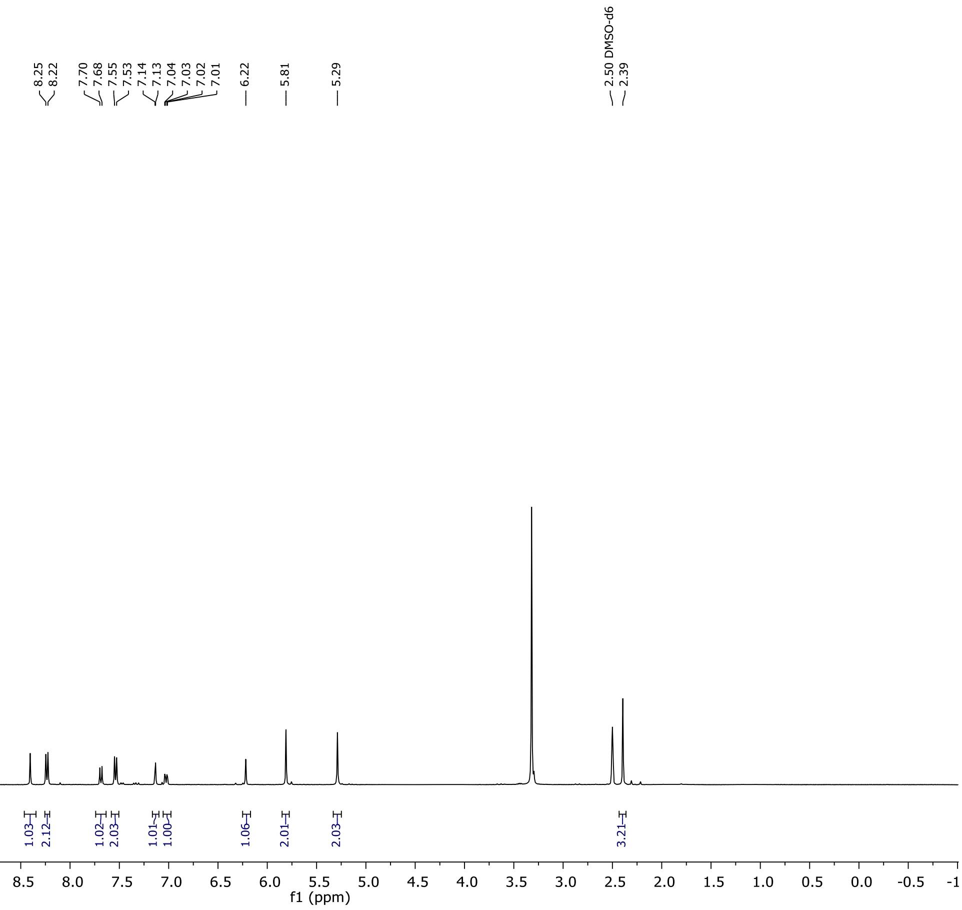

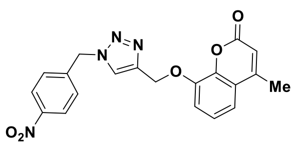

^{13}C NMR, 100 MHz, DMSO-d_6

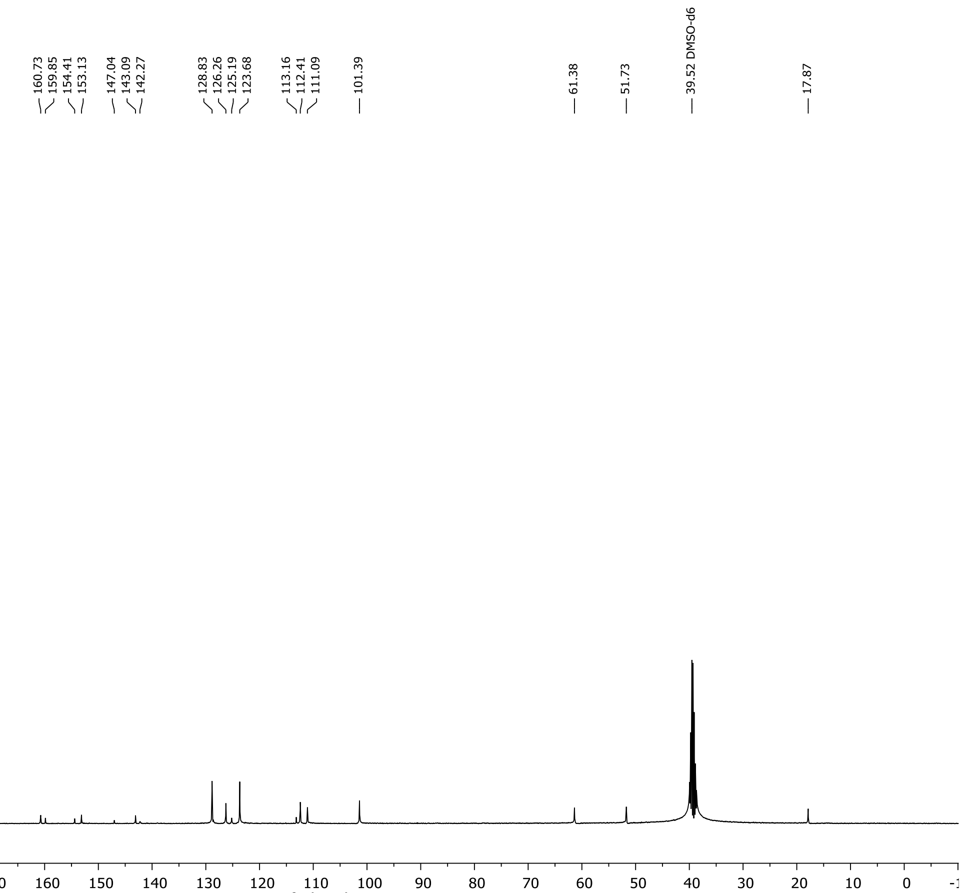

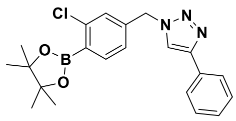

^{1}H NMR, 600 MHz, $CDCl_3$

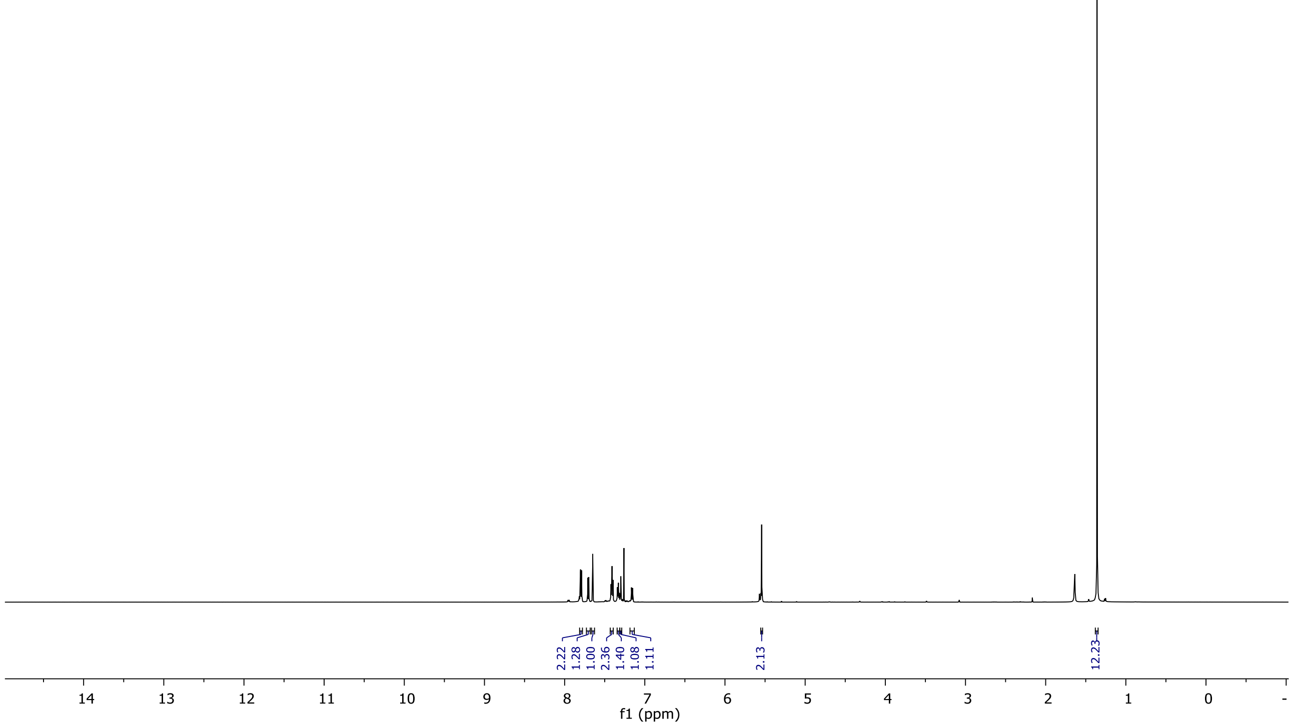

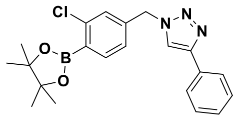

^{1}H NMR, 150 MHz, $CDCl_3$

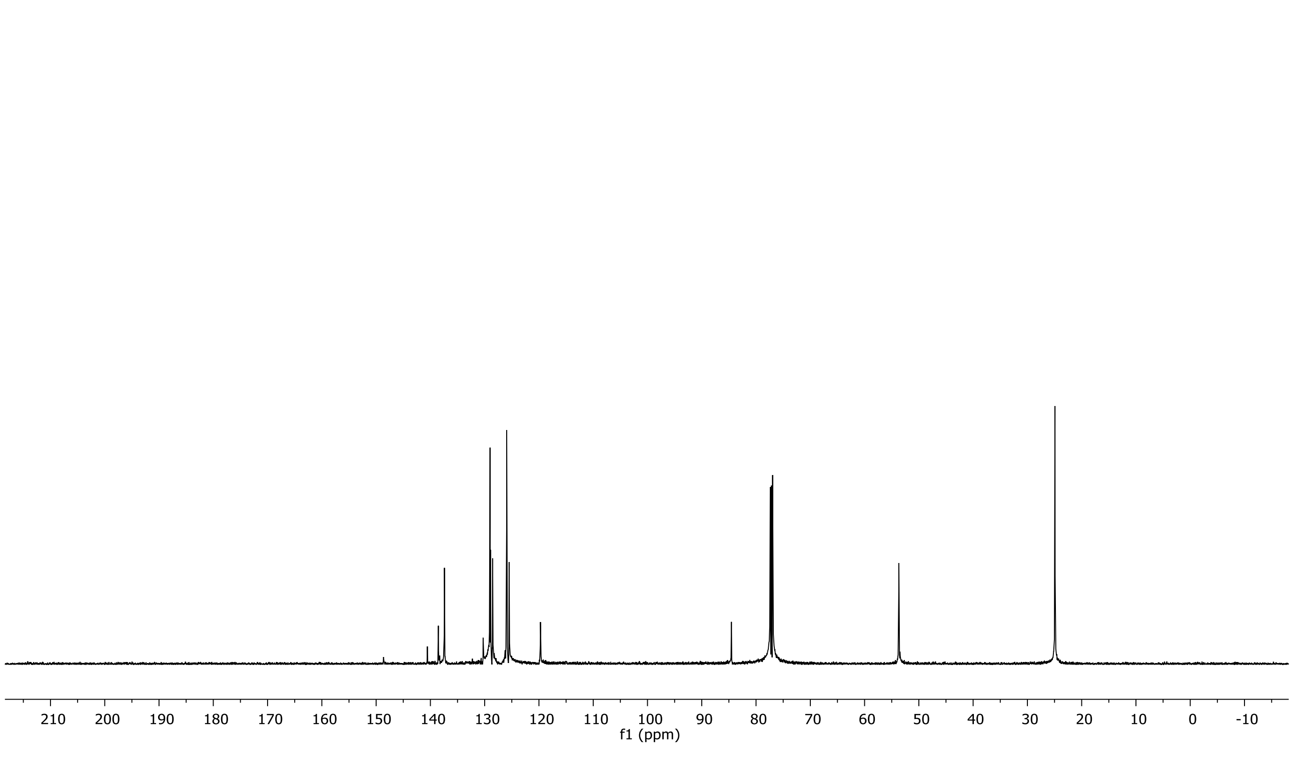

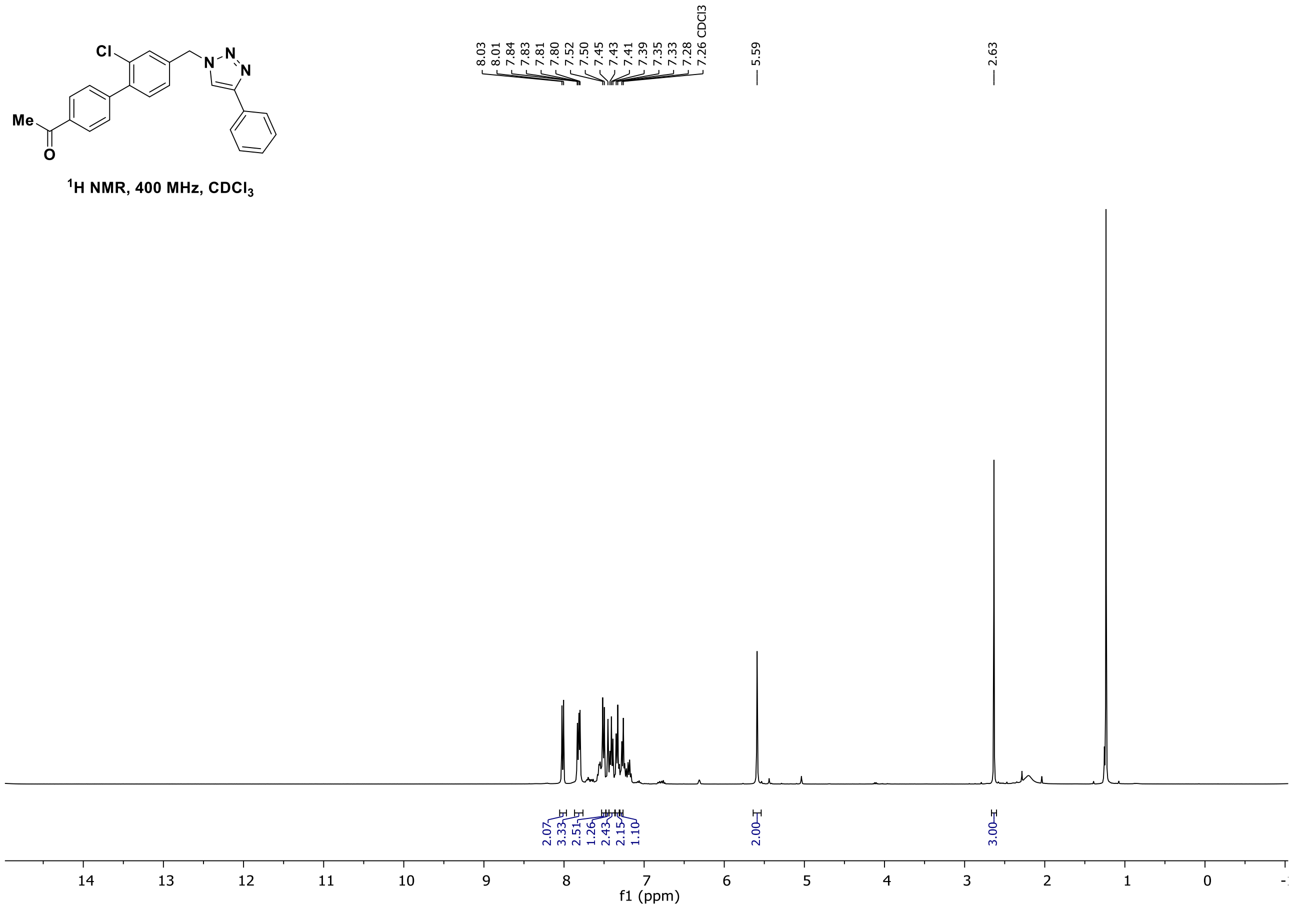

^{1}H NMR, 400 MHz, $CDCl_3$

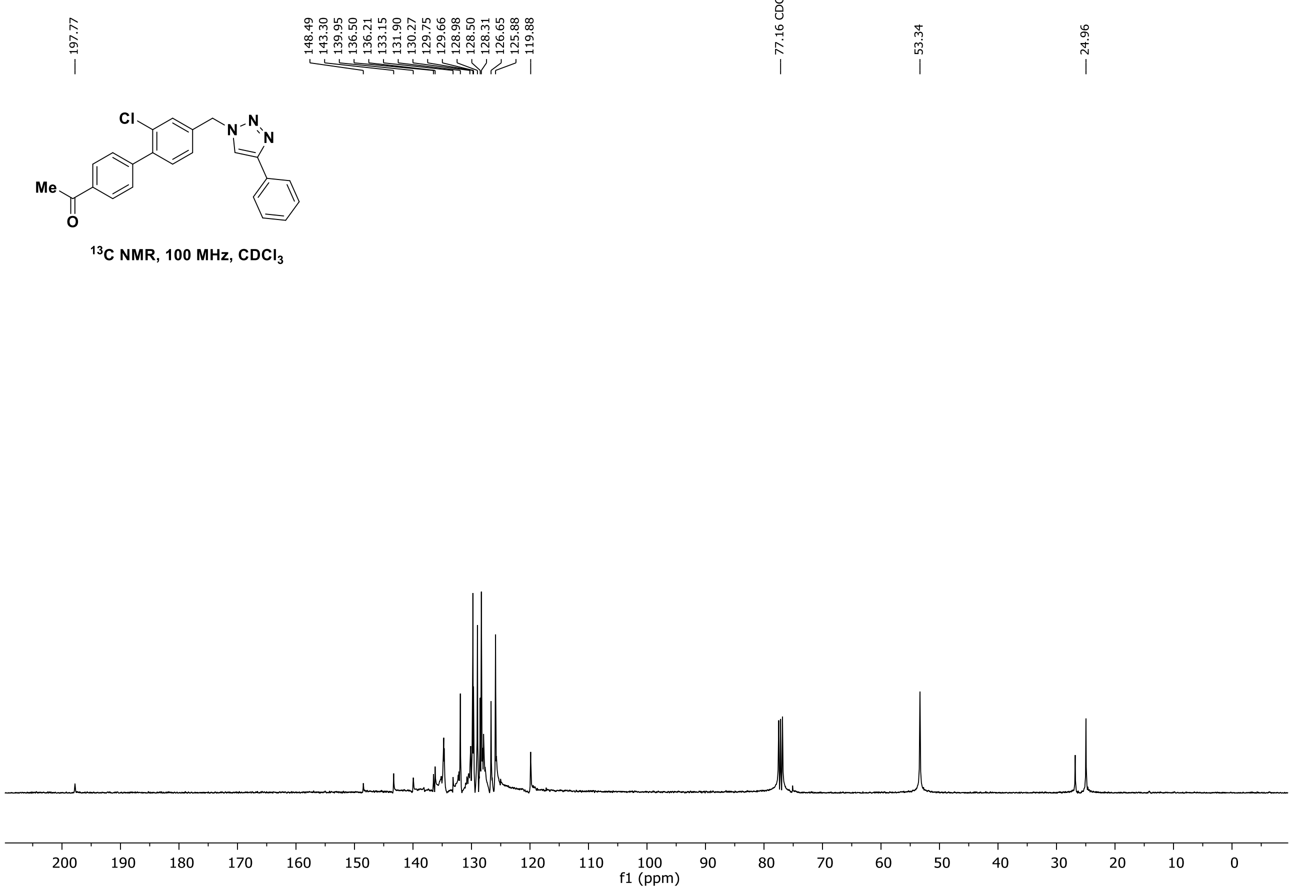

^{13}C NMR, 100 MHz, $CDCl_3$

HPLC traces of representative compounds

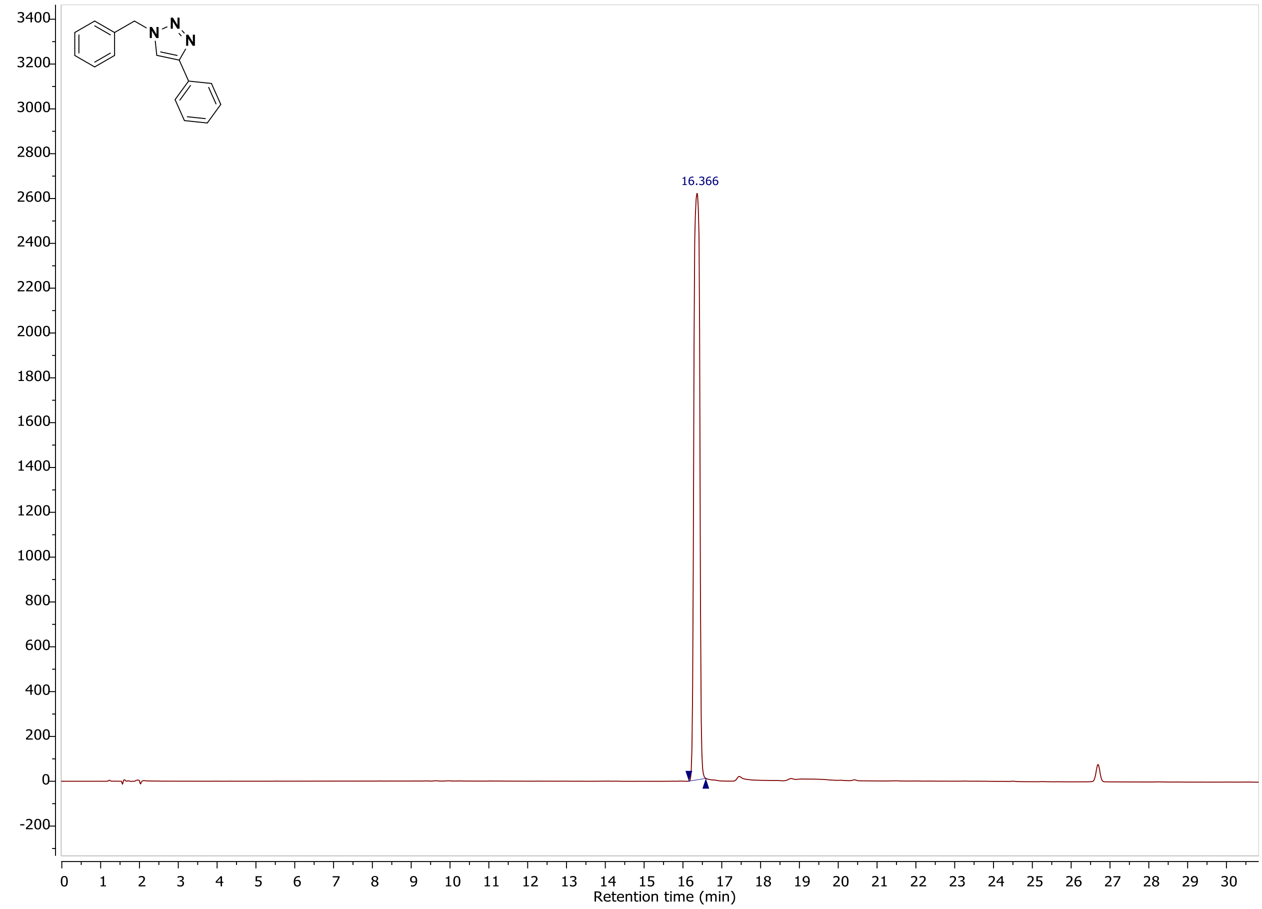

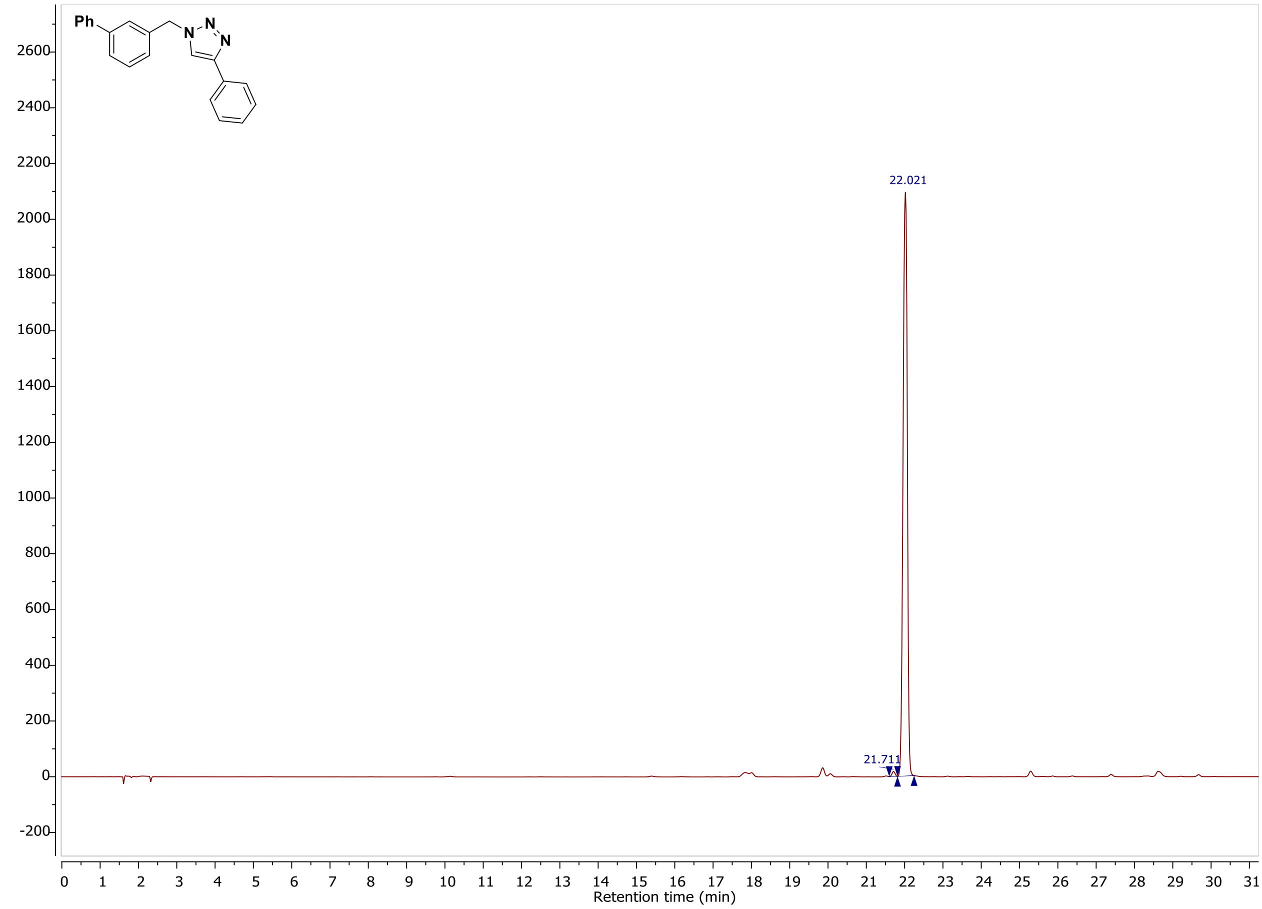

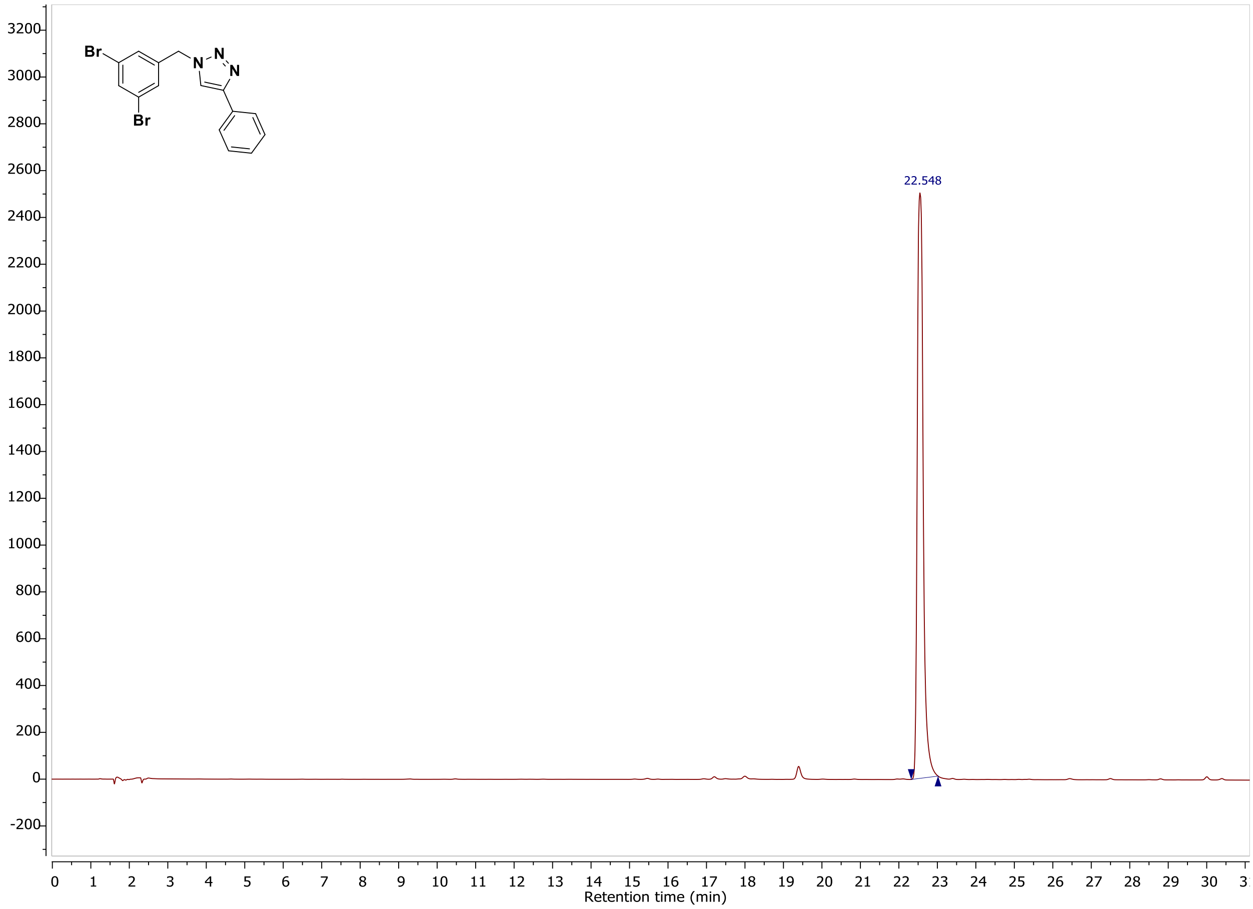
Br
N
N
N
Br
22.548
3200
3000
2800
2600
2400
2200
2000
1800
1600
1400
1200
1000
800
600
400
200
0
-200
Retention time (min)

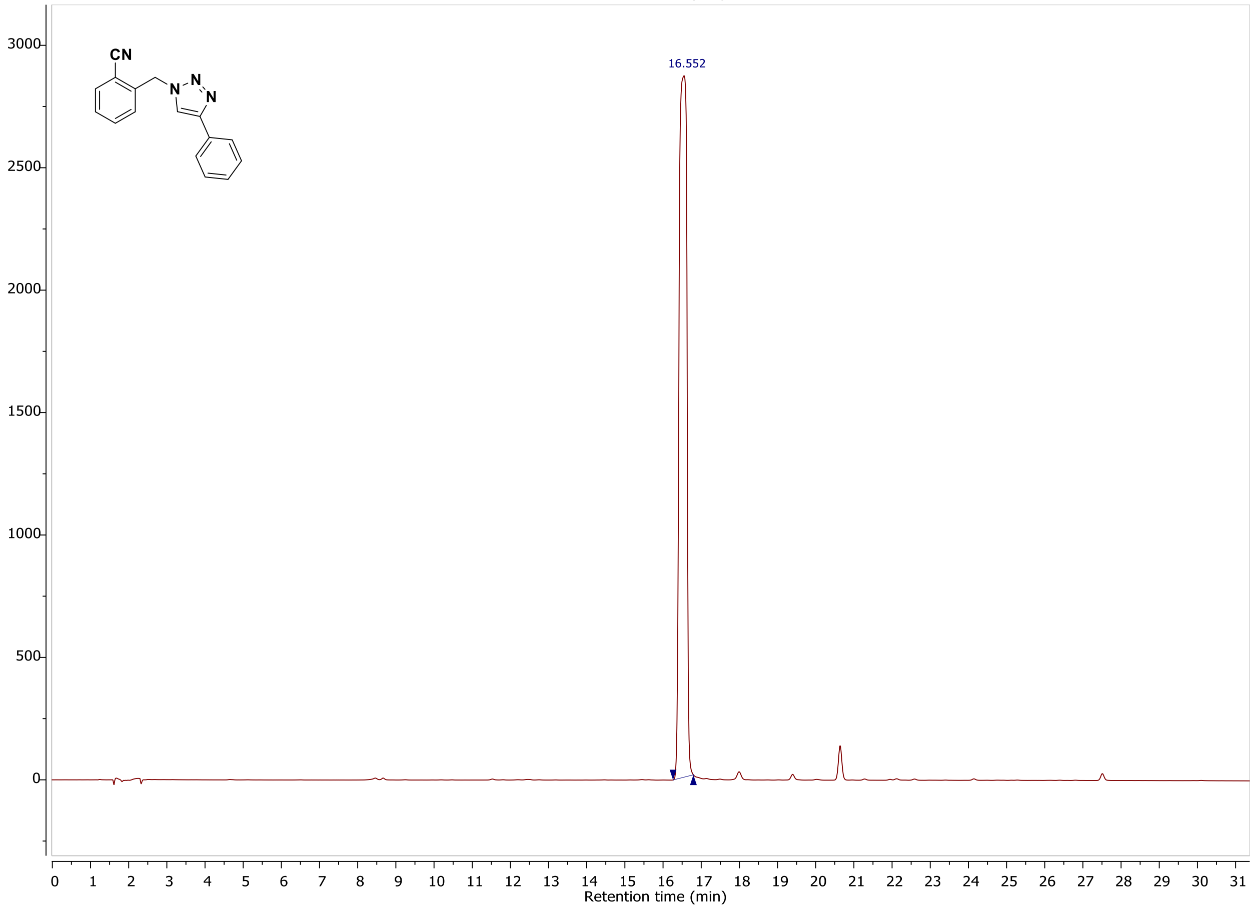
CN
N
N
N
16.552
3000
2500
2000
1500
1000
500
0
Retention time (min)

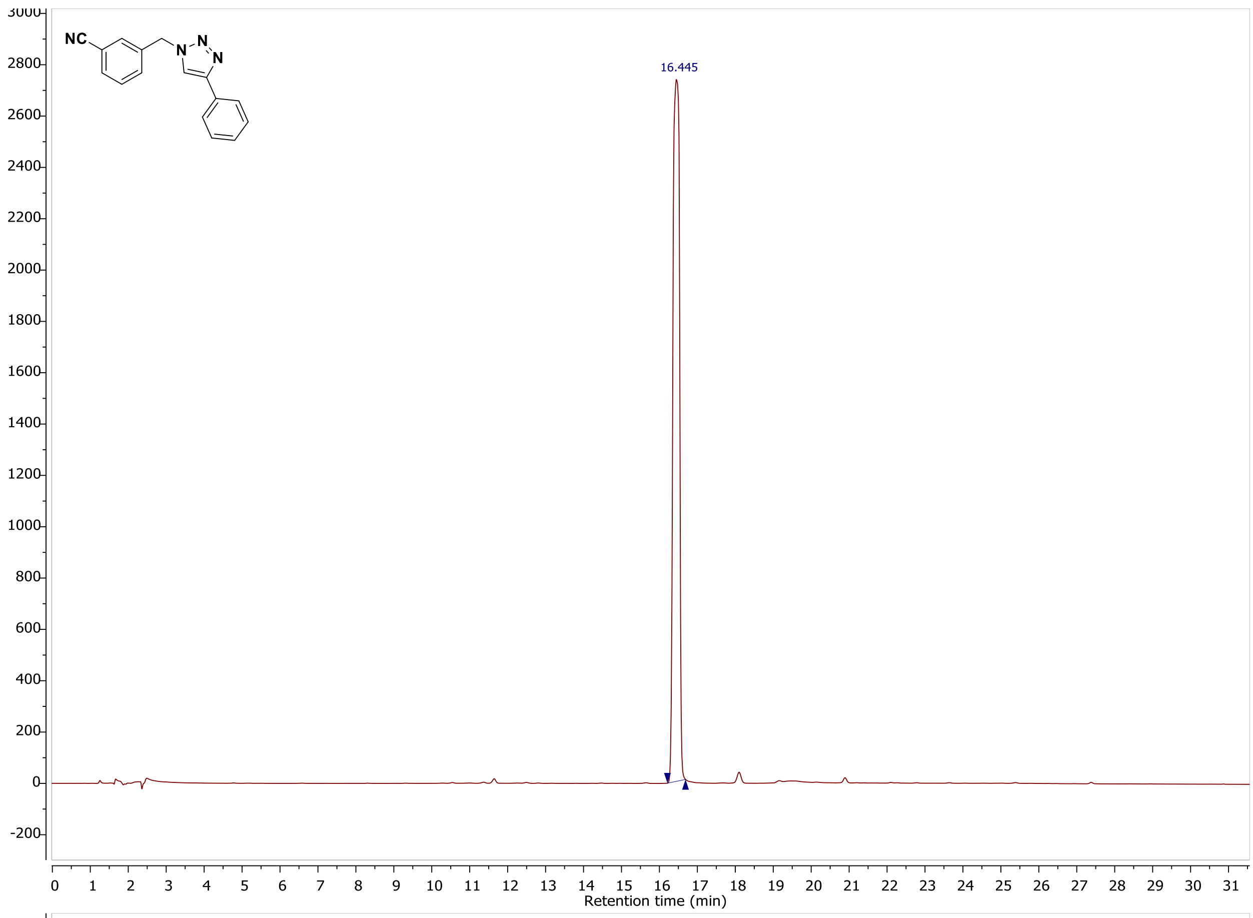
NC
N
N
N
16.445
Retention time (min)

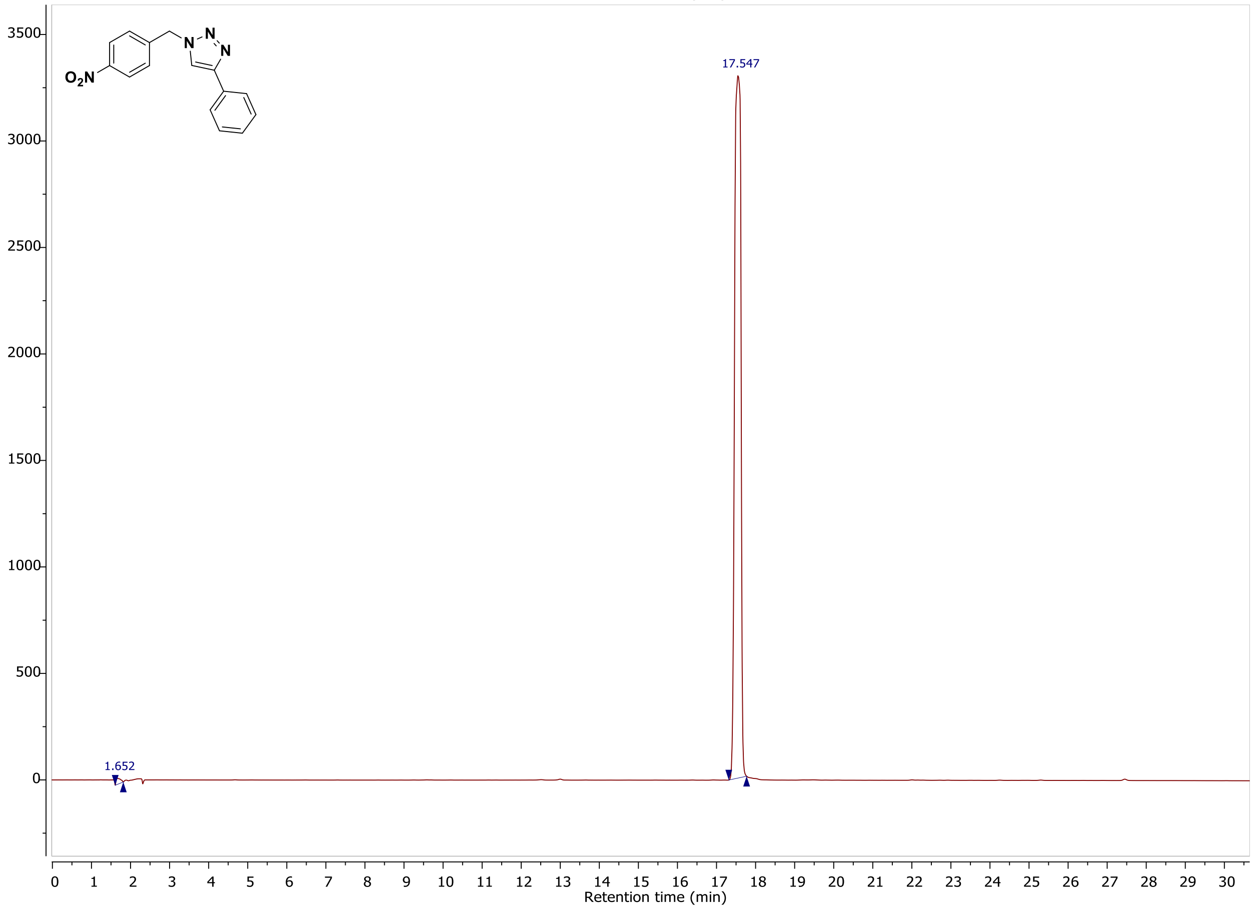
O2N
N
N
N
17.547
1.652
Retention time (min)

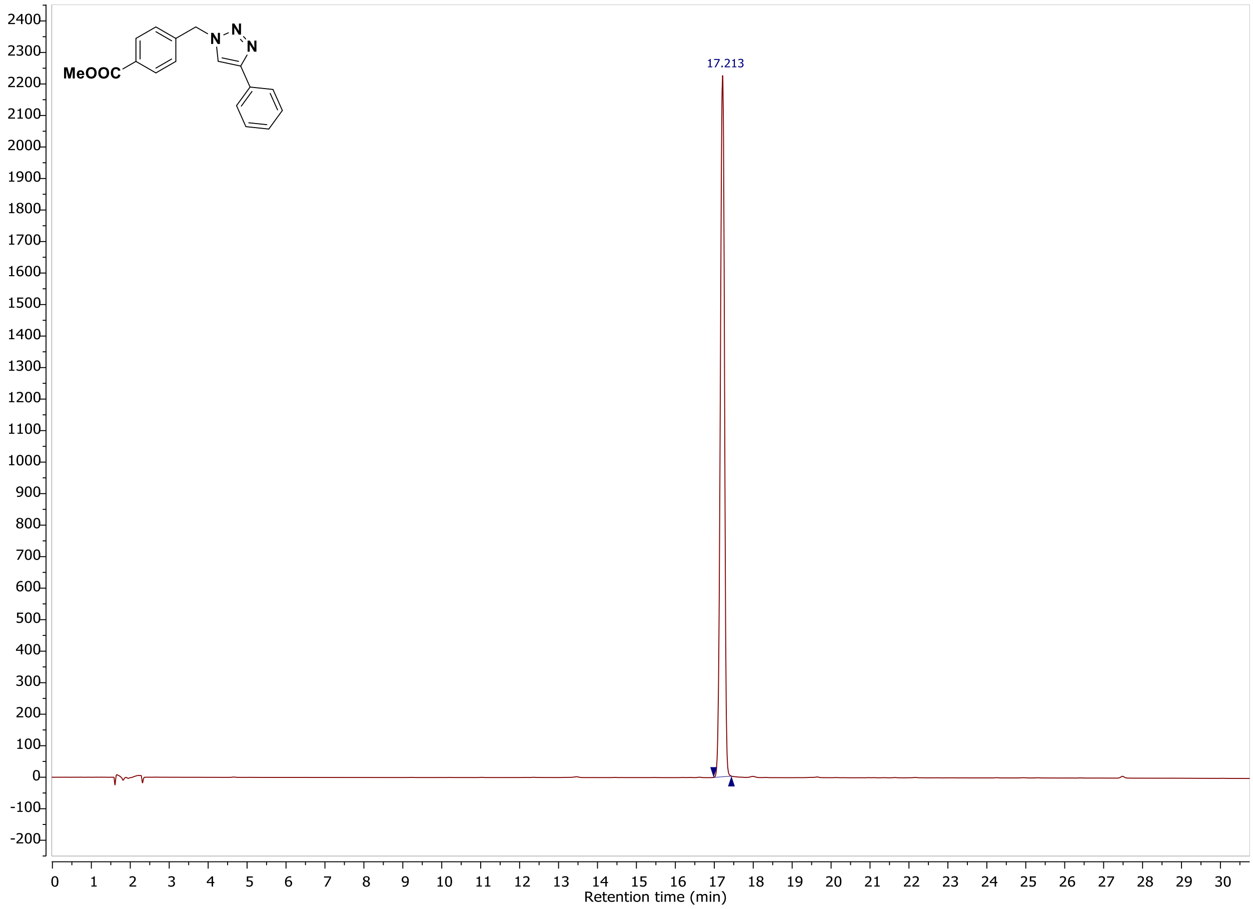
MeOOC
17.213
Retention time (min)

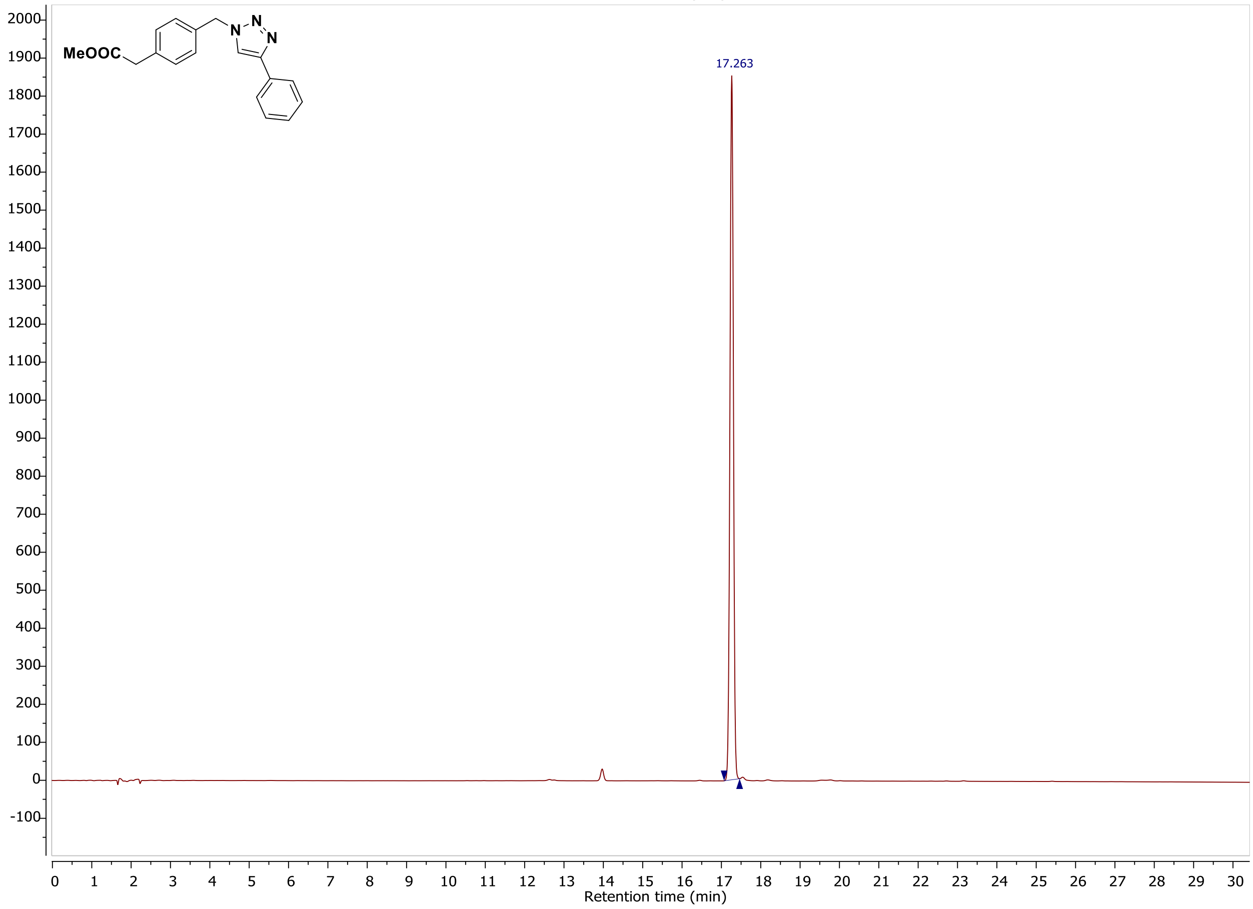
MeOOC
17.263
Retention time (min)

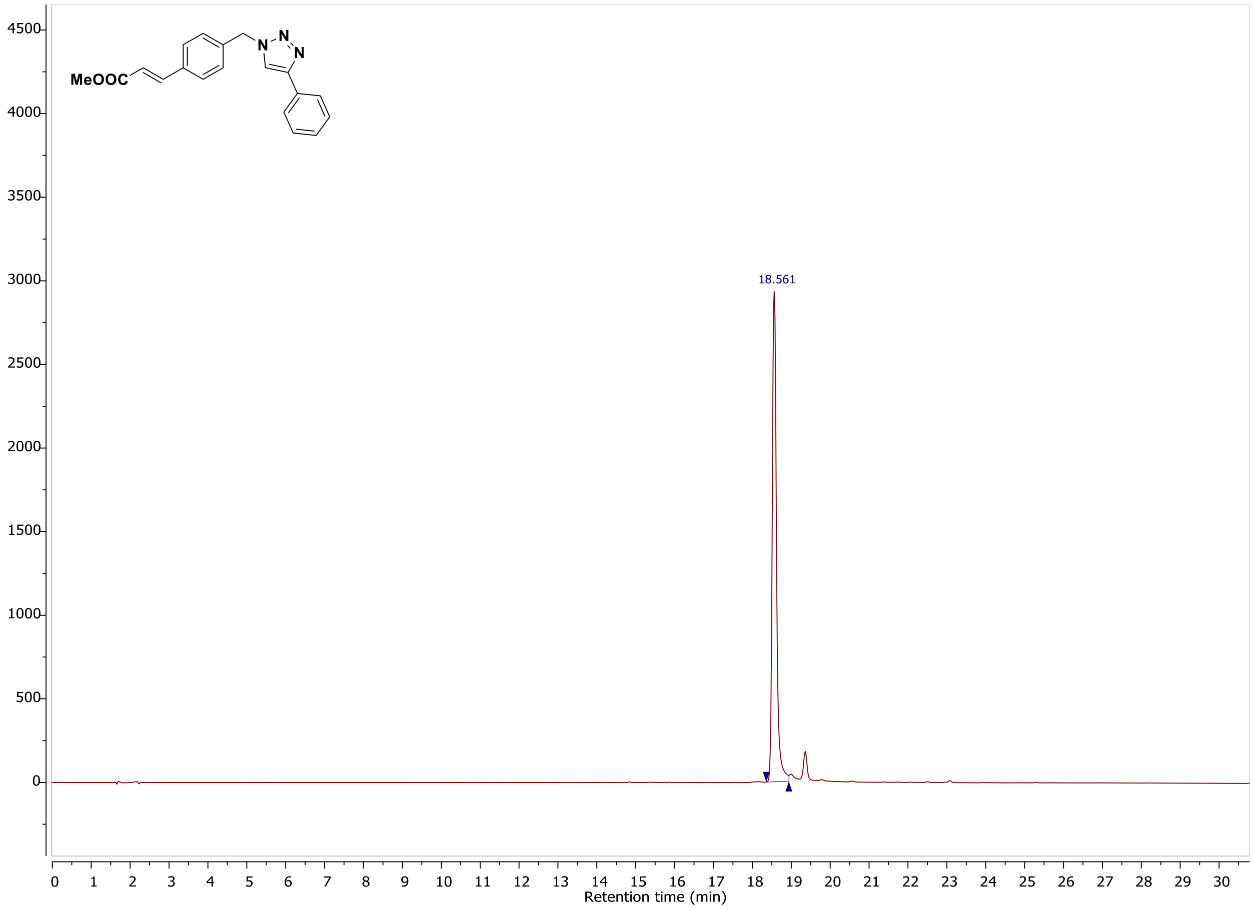
MeOOC
N
N
N
18.561
4500
4000
3500
3000
2500
2000
1500
1000
500
0
0 1 2 3 4 5 6 7 8 9 10 11 12 13 14 15 16 17 18 19 20 21 22 23 24 25 26 27 28 29 30
Retention time (min)

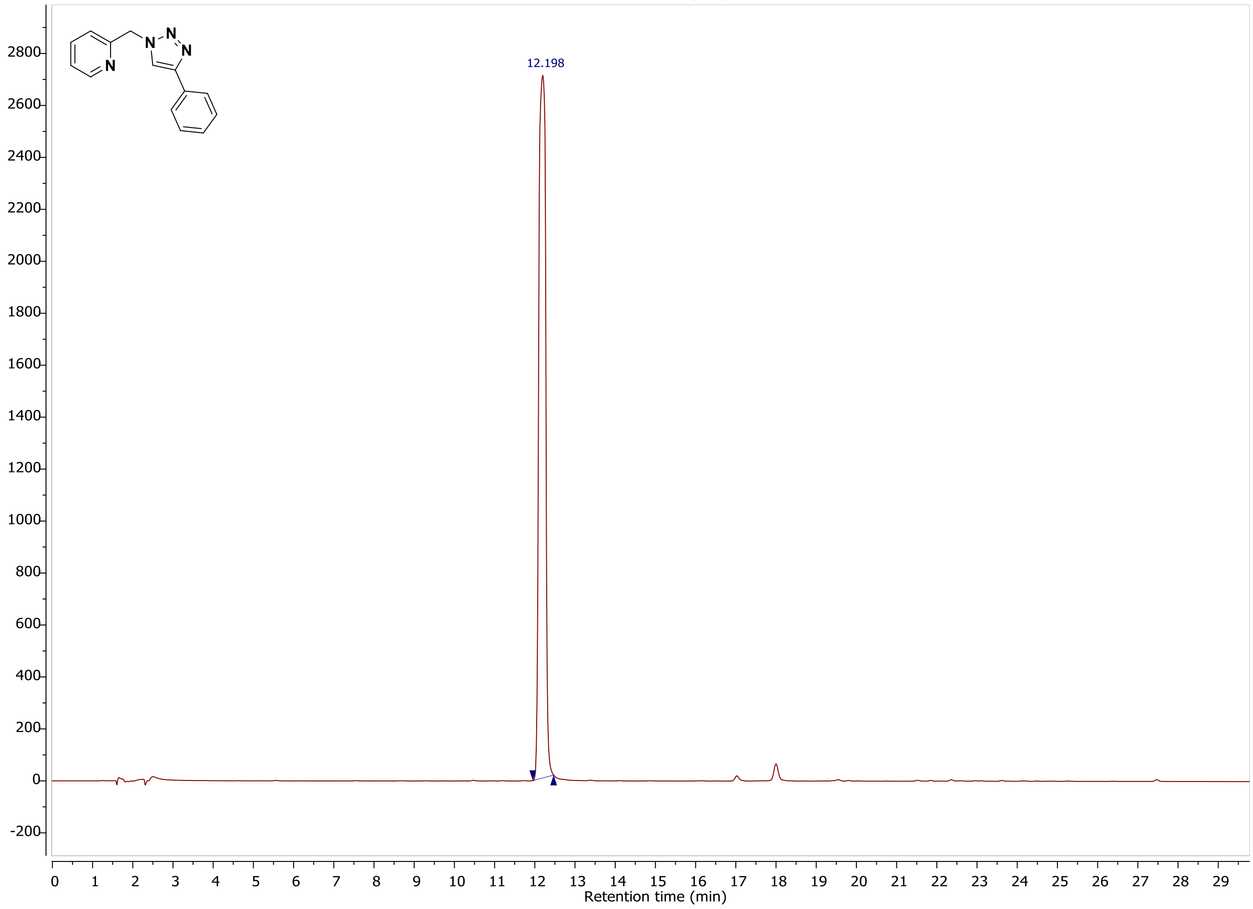
N
N
N
N
12.198
2800
2600
2400
2200
2000
1800
1600
1400
1200
1000
800
600
400
200
0
-200
0 1 2 3 4 5 6 7 8 9 10 11 12 13 14 15 16 17 18 19 20 21 22 23 24 25 26 27 28 29
Retention time (min)

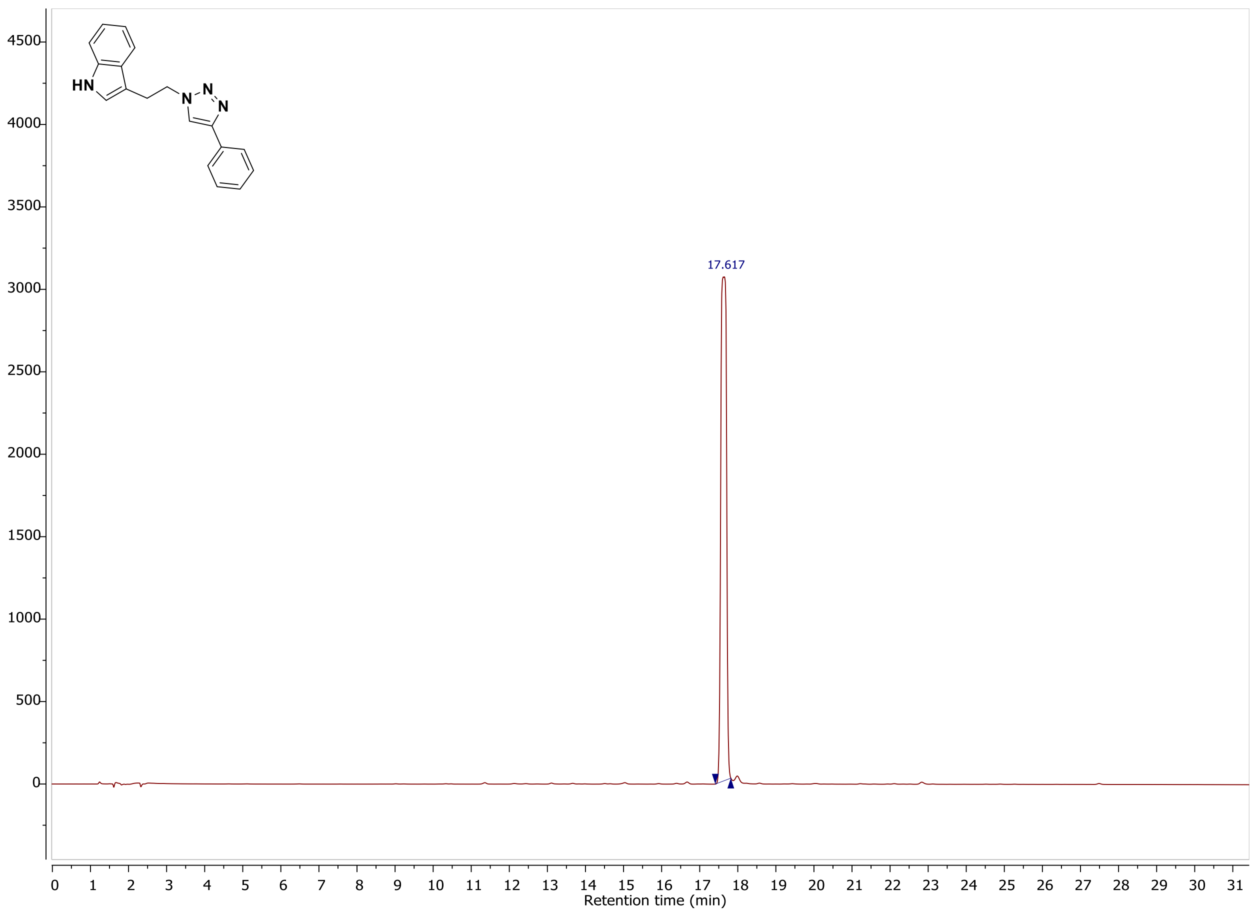
HN
N
N
N
17.617
4500
4000
3500
3000
2500
2000
1500
1000
500
0
0 1 2 3 4 5 6 7 8 9 10 11 12 13 14 15 16 17 18 19 20 21 22 23 24 25 26 27 28 29 30 31
Retention time (min)

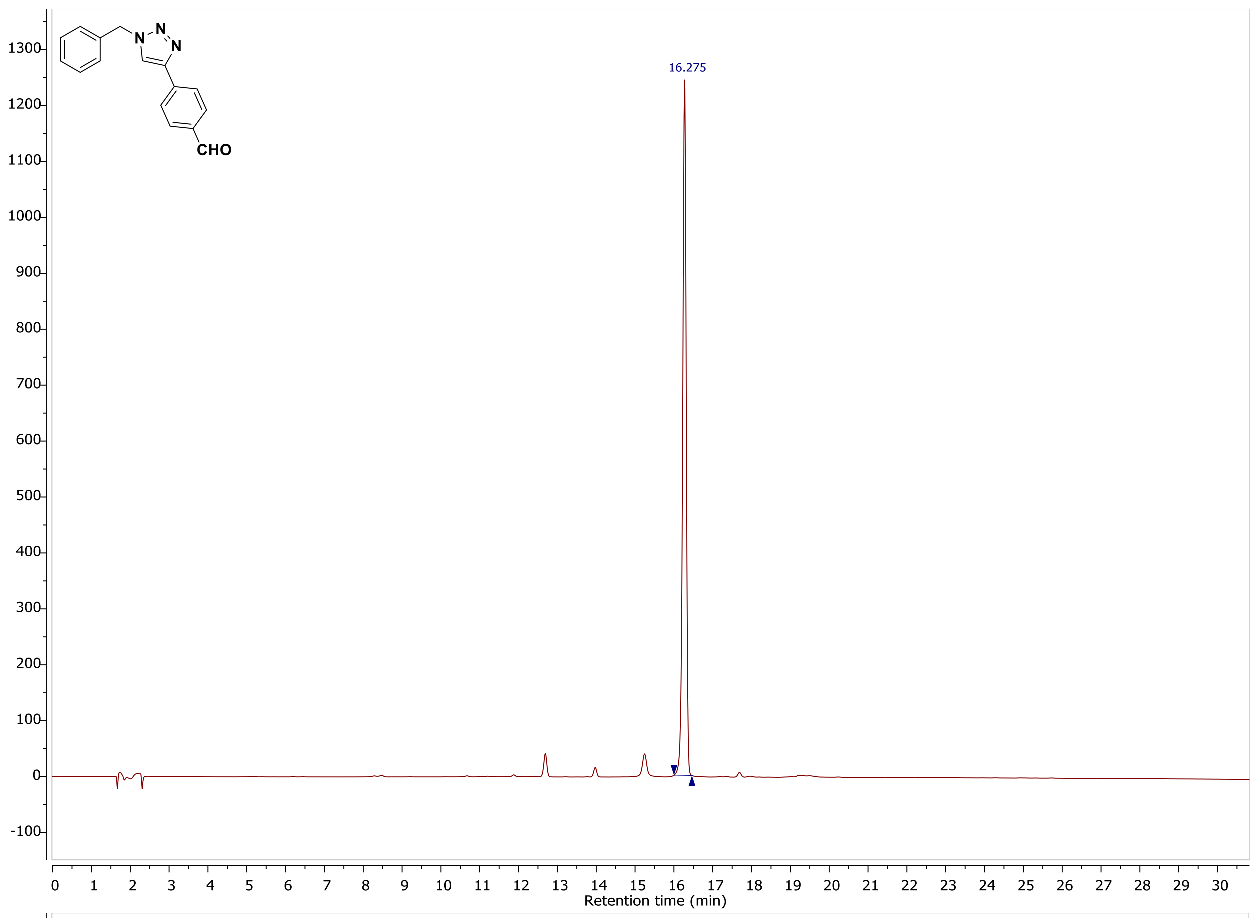
N
N
N
CHO
16.275
Retention time (min)

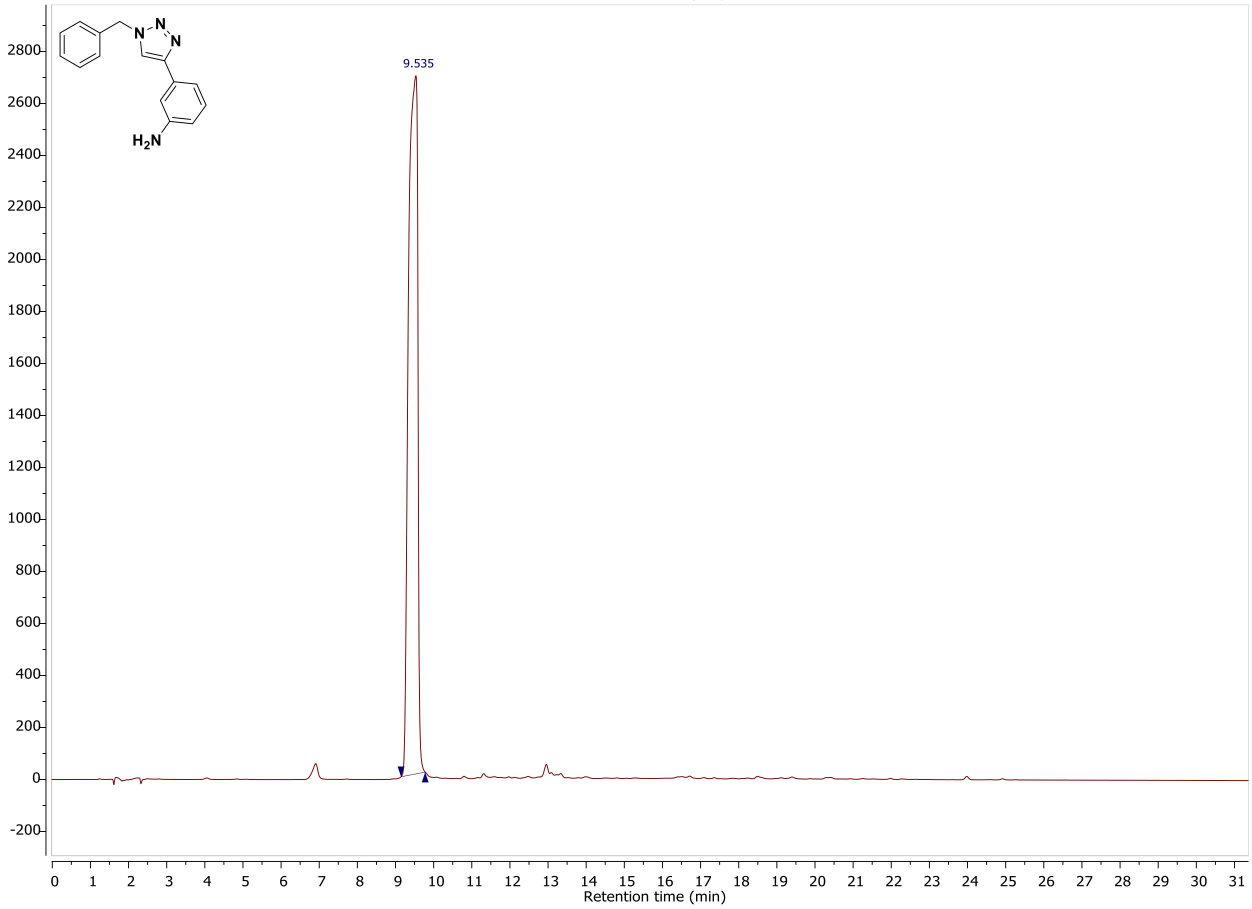
N
N
N
H2N
9.535
Retention time (min)

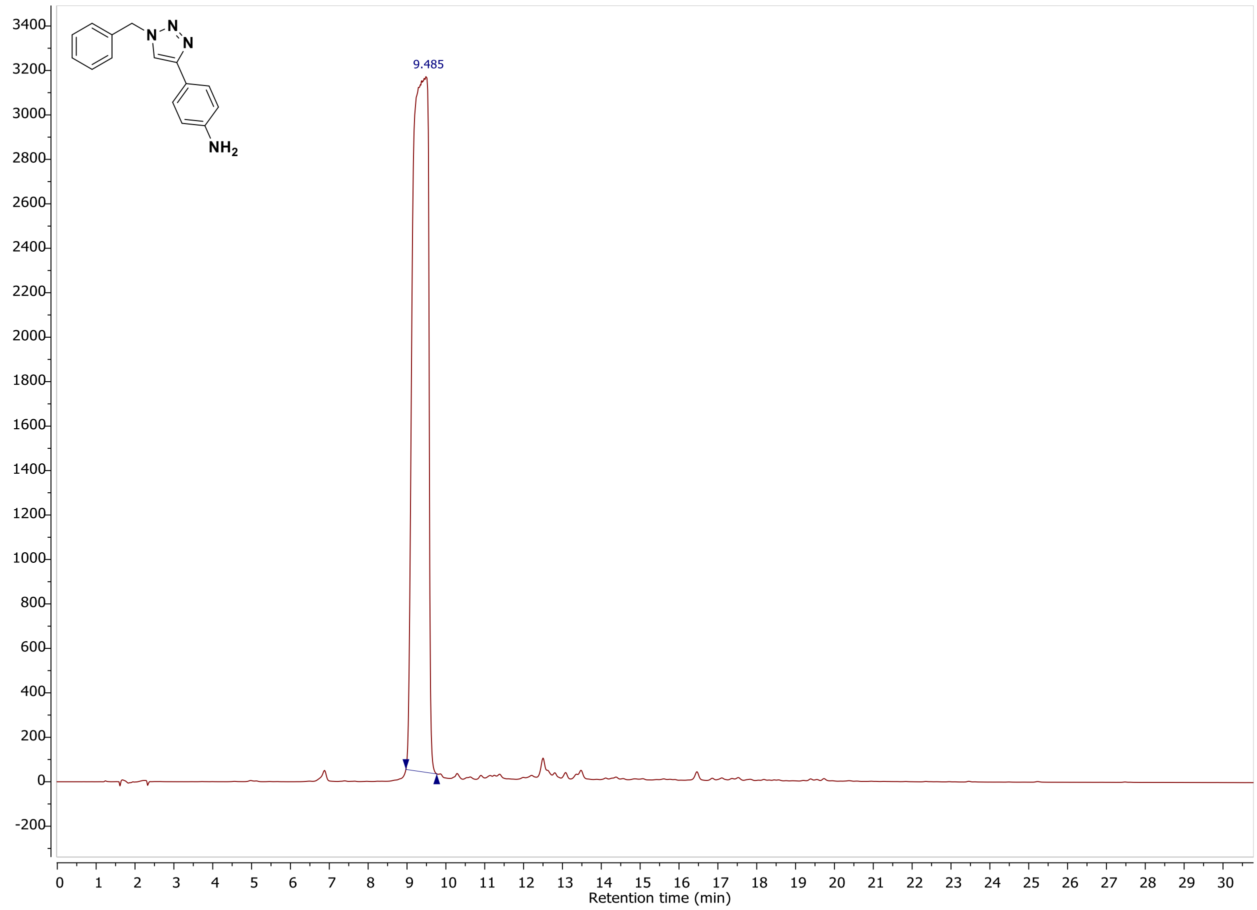
N
N
N
NH2
9.485
3400
3200
3000
2800
2600
2400
2200
2000
1800
1600
1400
1200
1000
800
600
400
200
0
-200
0 1 2 3 4 5 6 7 8 9 10 11 12 13 14 15 16 17 18 19 20 21 22 23 24 25 26 27 28 29 30
Retention time (min)

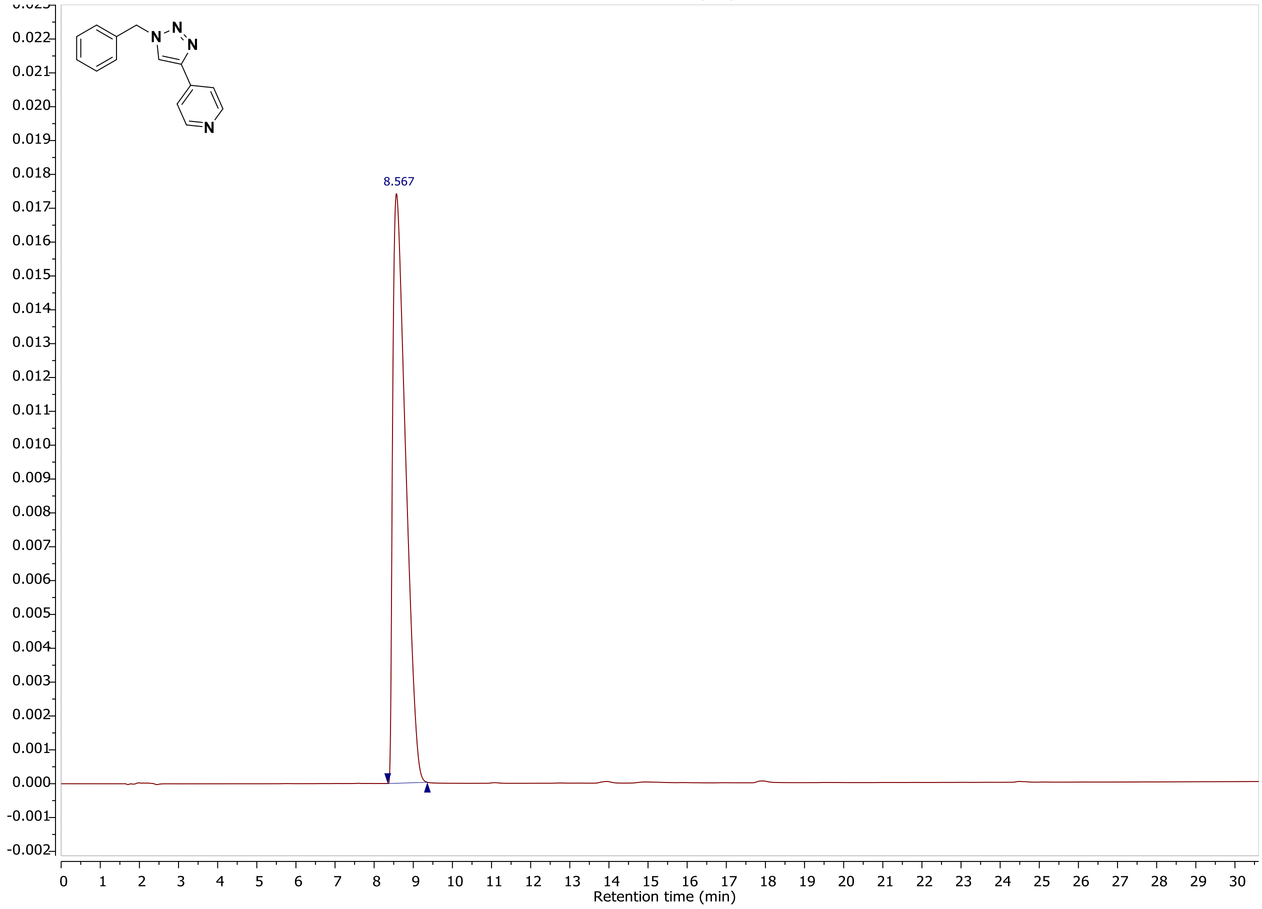
N
N
N
N
8.567
0.022
0.021
0.020
0.019
0.018
0.017
0.016
0.015
0.014
0.013
0.012
0.011
0.010
0.009
0.008
0.007
0.006
0.005
0.004
0.003
0.002
0.001
0.000
-0.001
-0.002
0 1 2 3 4 5 6 7 8 9 10 11 12 13 14 15 16 17 18 19 20 21 22 23 24 25 26 27 28 29 30
Retention time (min)

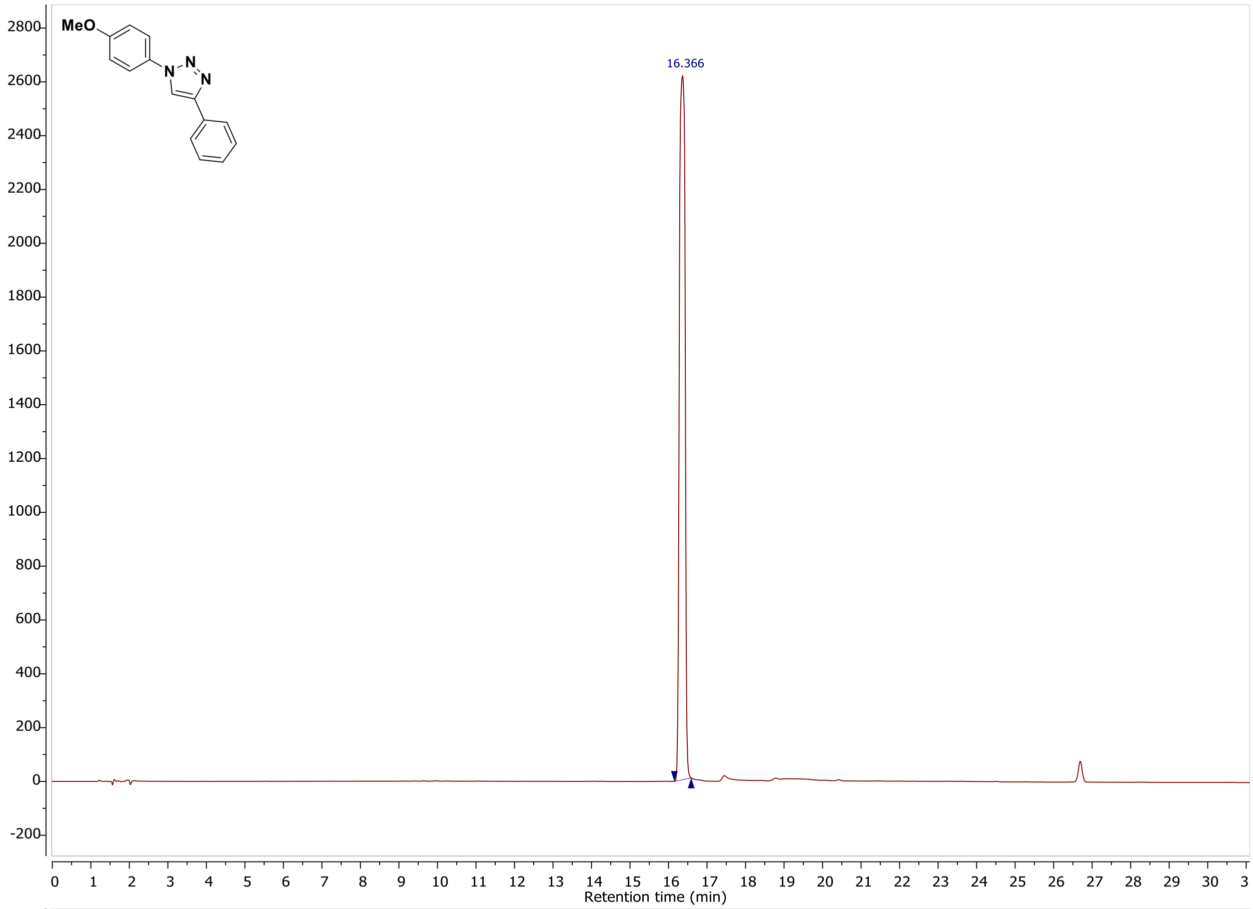
MeO
N
N
N
16.366
2800
2600
2400
2200
2000
1800
1600
1400
1200
1000
800
600
400
200
0
-200
0 1 2 3 4 5 6 7 8 9 10 11 12 13 14 15 16 17 18 19 20 21 22 23 24 25 26 27 28 29 30 3
Retention time (min)

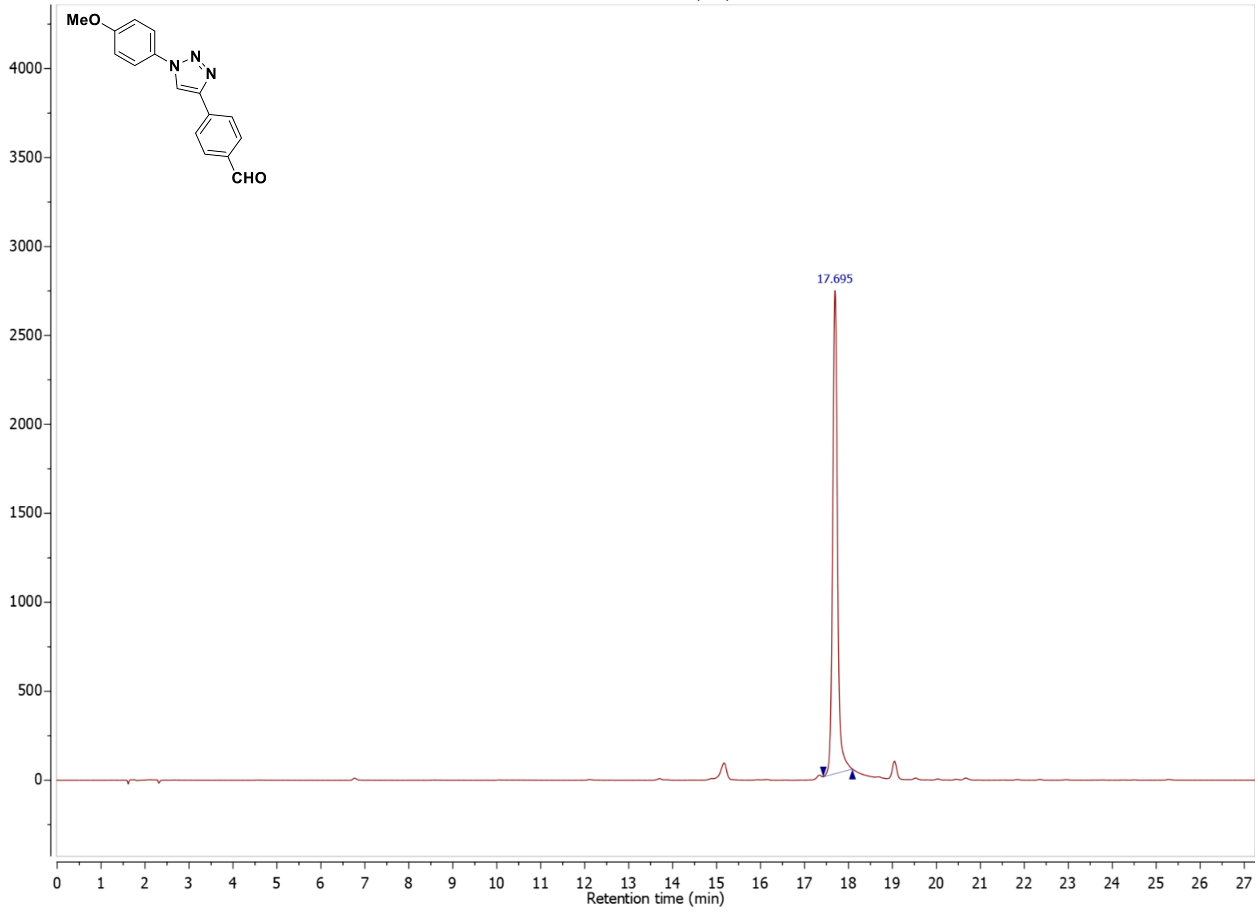
MeO
N
N
N
CHO
17.695
4000
3500
3000
2500
2000
1500
1000
500
0
0 1 2 3 4 5 6 7 8 9 10 11 12 13 14 15 16 17 18 19 20 21 22 23 24 25 26 27
Retention time (min)

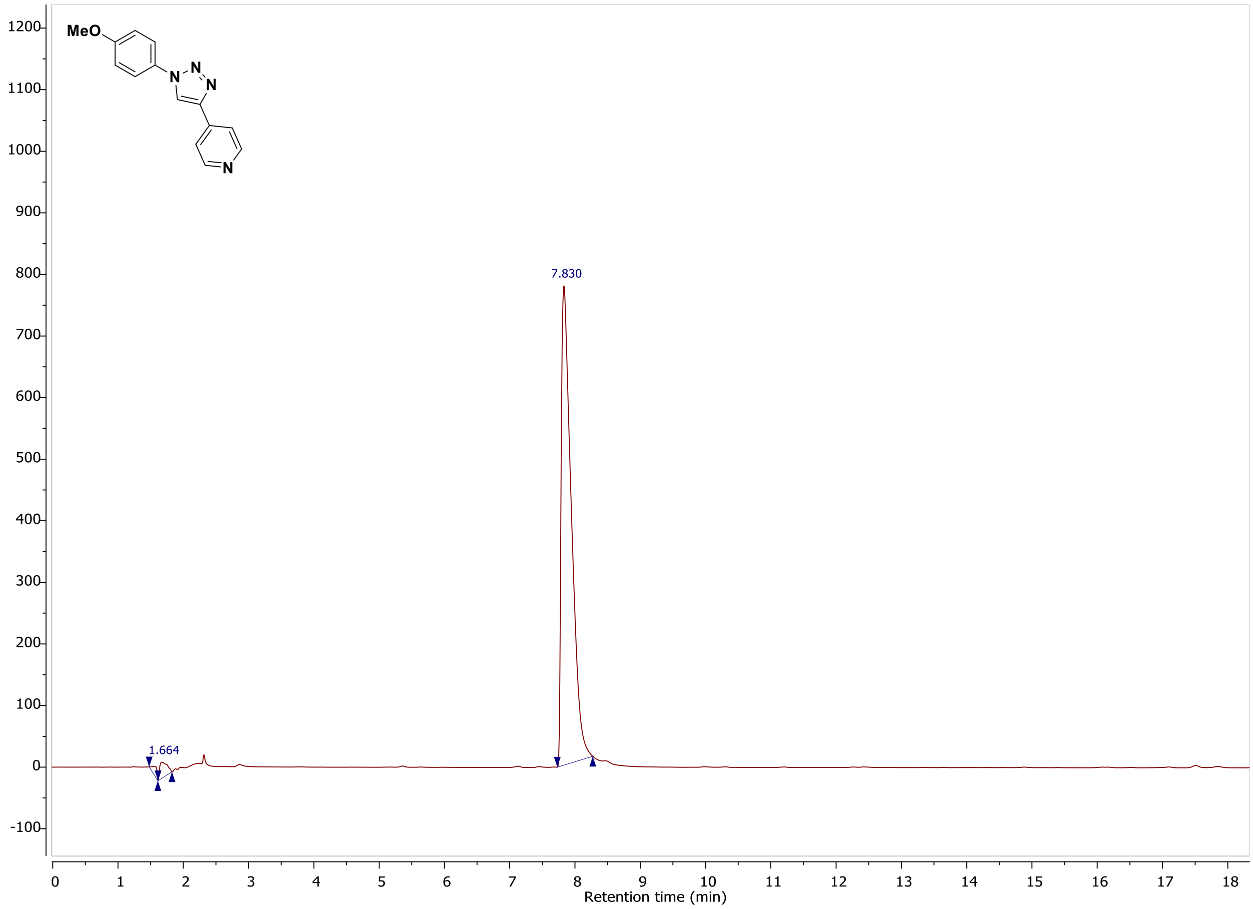
MeO
N
N
N
N
7.830
1.664
Retention time (min)

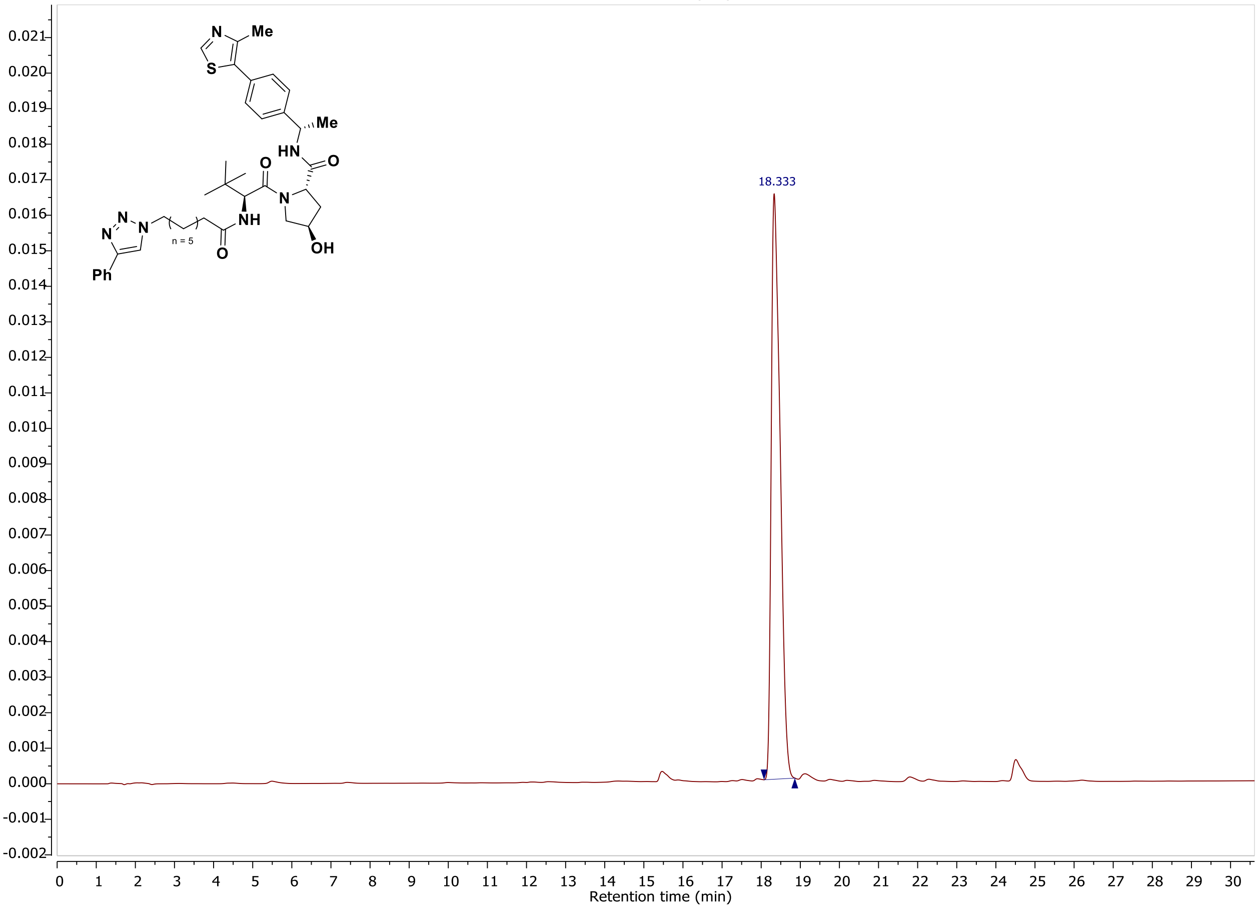
N
Me
S
Me
HN
O
O
N
NH
N
N
N
n = 5
O
OH
Ph
18.333
Retention time (min)

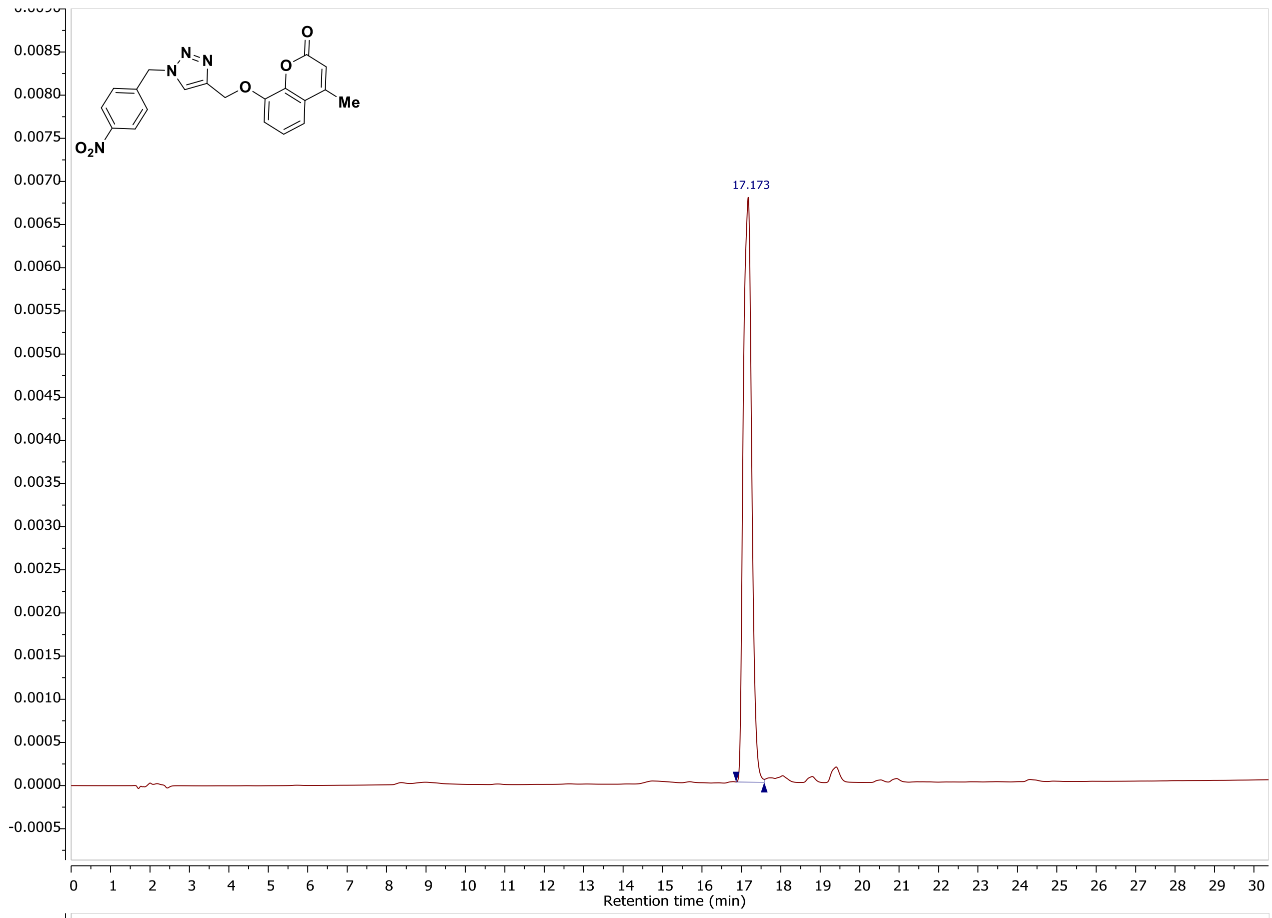
O
N
N
N
O
O
Me
O_2N
17.173
Retention time (min)

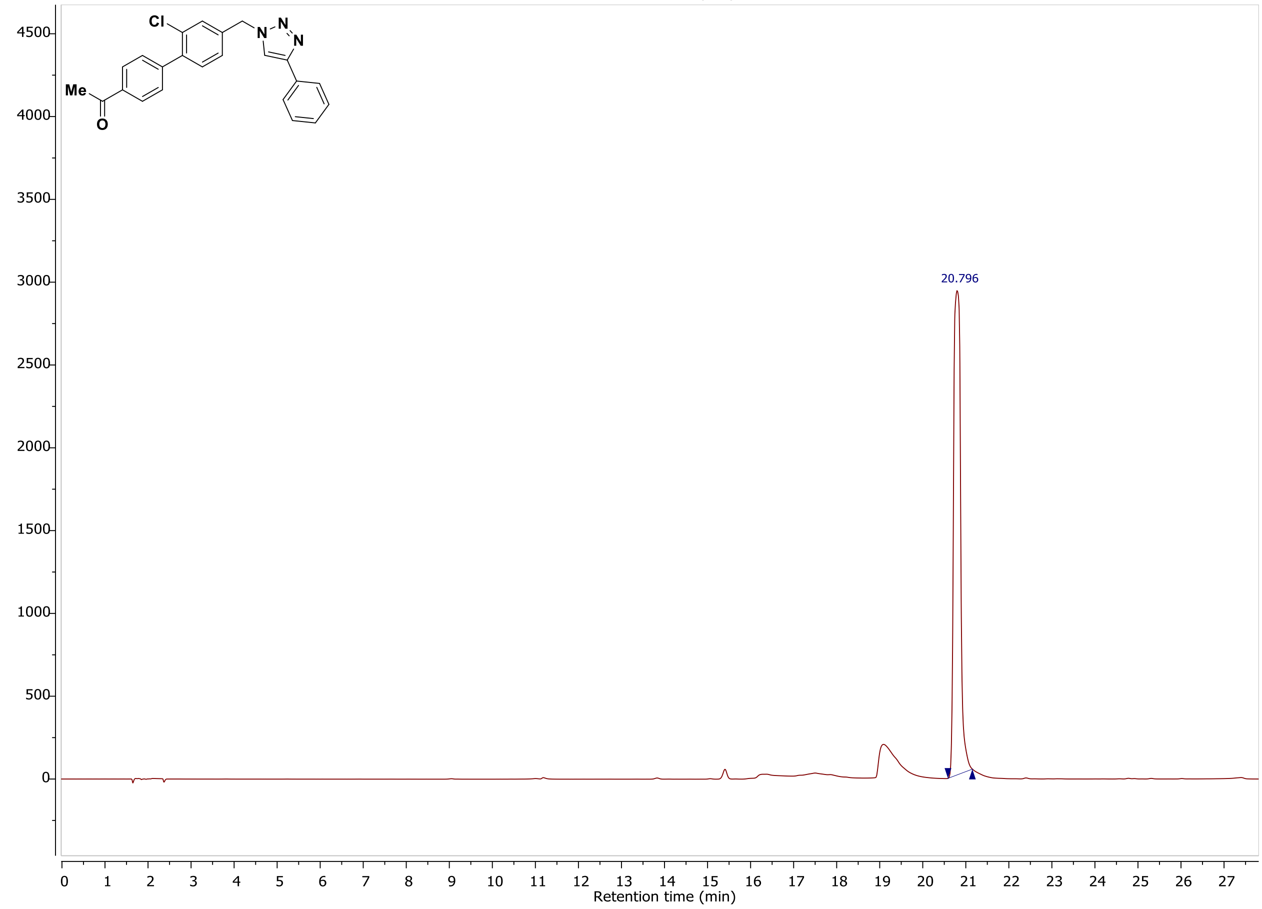
Cl
N
N
N
Me
O
20.796
Retention time (min)

E-factor Calculation

To assess the environmental impact of the developed protocol, E-factor were obtained basing on a procedure reported in the literature.[17] The E-factor calculation was performed on the model reaction (Scheme 1S) involving phenylacetylene 1 and benzyl azide 2, in the presence of $CuSO_4$ and sodium ascorbate, using two different solvents: DMSO (a non-green solvent) and Cyrene (a green solvent). 2 g scale was employed and 10% mol of $CuSO_4$ and sodium ascorbate were used.

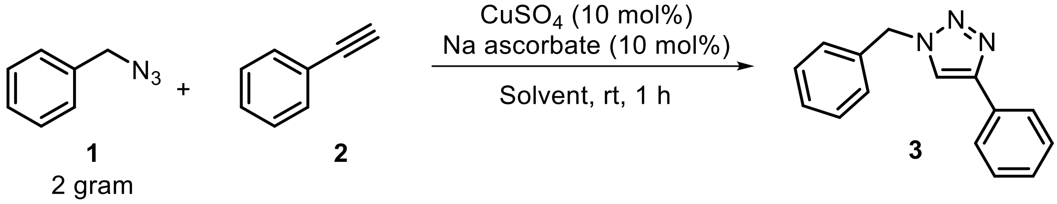

Scheme 1S. Model reaction for E-factor calculation.

Procedure with DMSO. A mixture of benzyl azide (2 g, 15 mmol, 1.0 equiv), phenylacetylene (1.78 g, 16.5 mmol, 1.1 equiv), $CuSO_4 \cdot 5H_2O$ (375 mg, 1.5 mmol, 0.1 equiv) sodium ascorbate (297 mg, 1.5 mmol, 0.1 equiv) in DMSO (10 mL) was left stirring at room temperature for 1 h. The crude reaction mixture was diluted with ethyl acetate and the organic phase was washed five times with brine, dried with anhydrous Na_2SO_4, filtered and concentrated under *vacuum* to afford the pure product (3.55 g, 96% yield).

Procedure with Cyrene. A mixture of benzyl azide (2 g, 15 mmol, 1.0 equiv), phenylacetylene (1.78 g, 16.5 mmol, 1.1 equiv), $CuSO_4 \cdot 5H_2O$ (375 mg, 1.5 mmol, 0.1 equiv) sodium ascorbate (297 mg, 1.5 mmol, 0.1 equiv) in Cyrene (10 mL) was left stirring at room temperature for 1 h. The reaction mixture was poured into ice-water and the precipitate was collected by filtration and washed with water to afford the pure product (3.5 g, 94% yield).

Waste calculation with DMSO. Following the protocol with DMSO, waste is determined by:

- DMSO that cannot be recovered (9.1 g)
- $CuSO_4$ (0.37 g)
- sodium ascorbate (0.3 g)
- Aqueous phases after work-up operation (70 g) containing unreacted reagents
- Organic solvent used for the extraction (ethyl acetate, 282 g)
- Sodium sulfate (8 g)

TOT WASTE: 370 g

Waste calculation with Cyrene. Following the protocol with Cyrene, waste is determined by:

- Cyrene that cannot be recovered (8 g)
- $CuSO_4$ (0.37 g)
- sodium ascorbate (0.3 g)
- Aqueous phases after filtration and washing procedure (74 g) containing unreacted reagents

TOT WASTE: 83 g

The mass of the desired product is 3.55 g (reaction in DMSO) and 3.5 (reaction in Cyrene).

Thus, the E-factor is calculated as:

E-factor = mass of waste generated / mass of desired product

$E\text{-factor}_{DMSO}$ = 104

$E\text{-factor}_{Cyrene}$ = 24

A confrontation of the two E-factors includes water as waste and provides a more comprehensive picture of the environmental impact of the reaction. While water is generally considered less problematic, its management can be significant in specific industrial contexts. In this case, water contains unreacted reagents (that could be toxic) and catalysts, so it is considered a waste. A lower E-factor in the Cyrene-based (24 versus 104) process highlights its significantly reduced environmental impact. This improvement is primarily attributed to the elimination of a work-up step, which, in the case of DMSO, generates a larger amount of waste and requires the use of an organic extraction solvent, sodium sulfate and subsequent use of a rotavapor—resulting in additional energy consumption. Using Cyrene reduces the E-factor and makes the reaction more sustainable compared to non-green solvents, even when including water as waste. This demonstrates how the use of biodegradable and recyclable solvents can improve the environmental profile of a process.

References

1. Rodionov, V. O.; Presolski, S. I.; Gardinier, S.; Lim, Y. H.; Finn, M. G., Benzimidazole and related ligands for Cu-catalyzed azide-alkyne cycloaddition. *J. Am. Chem. Soc.* **2007,** *129* (42), 12696-704.
2. Díaz-Marta, A. S.; Tubío, C. R.; Carbajales, C.; Fernández, C.; Escalante, L.; Sotelo, E.; Guitián, F.; Barrio, V. L.; Gil, A.; Coelho, A., Three-Dimensional Printing in Catalysis: Combining 3D Heterogeneous Copper and Palladium Catalysts for Multicatalytic Multicomponent Reactions. *ACS Catalysis* **2018,** *8* (1), 392-404.
3. Wang, S.; Jia, K.; Cheng, J.; Chen, Y.; Yuan, Y., Dual roles of substituted thiourea as reductant and ligand in CuAAC reaction. *Tetrahedron Lett.* **2017,** *58* (38), 3717-3721.
4. Drelinkiewicz, D.; Whitby, R. J., A practical flow synthesis of 1,2,3-triazoles. *RSC Adv.* **2022,** *12* (45), 28910-28915.
5. Katam, S.; Ganesan, P., Large Cu chalcogenone cubic cages with non-interacting counter ions. *Dalton Trans.* **2017,** *46* (47), 16615-16622.
6. Tahir, M. N.; Qamar, R.-u.; Adnan, A.; Cho, E.; Jung, S., Continuous process for click reactions using glass micro-reactor functionalized with β-cyclodextrin. *Tetrahedron Lett.* **2013,** *54* (25), 3268-3273.
7. Zhang, Q.; Li, Z.; Zhang, J.; Li, Y.; Pan, X.; Qu, J.; Zhang, J., Novel multi-target angiogenesis inhibitors as potential anticancer agents: Design, synthesis and preliminary activity evaluation. *Bioorg. Chem.* **2024,** *145*, 107211.
8. Chu, W. K.; Rono, C. K.; Makhubela, B. C. E., Synthesis, Structural Elucidation, and Cytotoxicity Studies of New Triazolyl Half-Sandwich RuII, OsII, RhIII and IrIII Complexes. *Eur. J. Inorg. Chem.* **2024,** *27* (2), e202300541.
9. Baig, R. B. N.; Varma, R. S., Copper on chitosan: a recyclable heterogeneous catalyst for azide–alkyne cycloaddition reactions in water. *Green Chem.* **2013,** *15* (7), 1839-1843.
10. Jiang, Y.; He, X.; Zhang, W.; Li, X.; Guo, N.; Zhao, Y.; Xu, G.; Li, W., Metallic copper wire: a simple, clear and reusable catalyst for the CuAAC reaction in supercritical carbon dioxide. *RSC Adv.* **2015,** *5* (90), 73340-73345.
11. Mansano Willig, J. C.; Granetto, G.; Reginato, D.; Dutra, F. R.; Poruczinski, É. F.; de Oliveira, I. M.; Stefani, H. A.; de Campos, S. D.; de Campos, É. A.; Manarin, F.; Botteselle, G. V., A comparative study between Cu(INA)2-MOF and [Cu(INA)2(H2O)4] complex for a click reaction and the Biginelli reaction under solvent-free conditions. *RSC Adv.* **2020,** *10* (6), 3407-3415.
12. Irie, T.; Fujii, I.; Sawa, M., Design and combinatorial synthesis of a novel kinase-focused library using click chemistry-based fragment assembly. *Bioorg. Med. Chem. Lett.* **2012,** *22* (1), 591-596.
13. Zahmatkesh, S.; Esmaeilpour, M.; Javidi, J., 1,4-Dihydroxyanthraquinone–copper(ii) supported on superparamagnetic Fe3O4@SiO2: an efficient catalyst for N-arylation of nitrogen heterocycles and alkylamines with aryl halides and click synthesis of 1-aryl-1,2,3-triazole derivatives. *RSC Adv.* **2016,** *6* (93), 90154-90164.
14. Pagliai, F.; Pirali, T.; Del Grosso, E.; Di Brisco, R.; Tron, G. C.; Sorba, G.; Genazzani, A. A., Rapid synthesis of triazole-modified resveratrol analogues via click chemistry. *J. Med. Chem.* **2006,** *49* (2), 467-70.
15. Shi, J.; Liu, L.; He, J.; Meng, X.; Guo, Q., Facile Derivatization of Pyridyloxazole-type Fluorophore via Click Chemistry. *Chemistry Lett.* **2007,** *36* (9), 1142-1143.
16. Yadav, N.; Agarwal, D.; Kumar, S.; Dixit, A. K.; Gupta, R. D.; Awasthi, S. K., In vitro antiplasmodial efficacy of synthetic coumarin-triazole analogs. *Eur. J. Med. Chem.* **2018,** *145*, 735-745.
17. Petrucci, C.; Strappaveccia, G.; Giacalone, F.; Gruttadauria, M.; Pizzo, F.; Vaccaro, L., An E-Factor Minimized Protocol for a Sustainable and Efficient Heck Reaction in Flow. *ACS Sustainable Chemistry & Engineering* **2014,** *2* (12), 2813-2819.
